# Neuroendocrine Carcinomas of the Gastroenteropancreatic System: A Comprehensive Review

**DOI:** 10.3390/diagnostics5020119

**Published:** 2015-04-08

**Authors:** Emma Elizabeth Ilett, Seppo W. Langer, Ingrid Holst Olsen, Birgitte Federspiel, Andreas Kjær, Ulrich Knigge

**Affiliations:** 1 Neuroendocrine Tumor Centre of Excellence, Rigshospitalet, University of Copenhagen, 2100 Copenhagen, Denmark; E-Mails: seppo.langer@regionh.dk (S.W.L.); ingridho@sund.ku.dk (I.H.O.); birgitte.federspiel@regionh.dk (B.F.); andreas.kjaer@regionh.dk (A.K.); ulrich.peter.knigge@regionh.dk (U.K.); 2 Department of Gastrointestinal Surgery C, Rigshospitalet, University of Copenhagen, 2100 Copenhagen, Denmark; 3 Department of Oncology, Rigshospitalet, University of Copenhagen, 2100 Copenhagen, Denmark; 4 Cluster for Molecular Imaging, Faculty of Health Sciences, University of Copenhagen, 2100 Copenhagen, Denmark; 5 Department of Pathology, Rigshospitalet, University of Copenhagen, 2100 Copenhagen, Denmark; 6 Department of Clinical Physiology, Nuclear Medicine and PET, Rigshospitalet, University of Copenhagen, 2100 Copenhagen, Denmark; 7 Deparment of Endocrinology, Rigshospitalet, University of Copenhagen, 2100 Copenhagen, Denmark

**Keywords:** neuroendocrine carcinomas, neuroendocrine tumours, neuroendocrine neoplasms, small cell carcinomas, large cell carcinomas, Ki-67 index, oesophagus, stomach, pancreas, colo-rectal

## Abstract

To date, empirical literature has generally been considered lacking in relation to neuroendocrine carcinomas (NECs), the highly malignant subgroup of neuroendocrine neoplasms. NECs are often found in the lungs or the gastroenteropancreatic (GEP) system and can be of small or large cell type. Concentrating on GEP-NECs, we can conclude that survival times are poor, with a median of only 4–16 months depending on disease stage and primary site. Further, this aggressive disease appears to be on the rise, with incidence numbers increasing while survival times are stagnant. Treatment strategies concerning surgery are often undecided and second-line chemotherapy is not yet established. After an analysis of over 2600 articles, we can conclude that there is indeed more empirical literature concerning GEP-NECs available than previously assumed. This unique review is based on 333 selected articles and contains detailed information concerning all aspects of GEP-NECs. Namely, the classification, histology, genetic abnormalities, epidemiology, origin, biochemistry, imaging, treatment and survival of GEP-NECs are described. Also, organ-specific summaries with more detail in relation to disease presentation, diagnosis, treatment and survival are presented. Finally, key points are discussed with directions for future research priorities.

## 1. Introduction

Neuroendocrine carcinomas (NECs) are a rare, highly malignant subgroup of neuroendocrine neoplasms (NENs) that can arise in almost any organ of the body. NENs are defined as epithelial neoplasms with predominant neuroendocrine differentiation. These tumours originate from the peripheral neuroendocrine cell system dispersed in all organs. The most common location for NENs is the lungs and organs of the gastroenteropancreatic (GEP) system. Some clinical and pathological features of NECs are characteristic of the organ of origin, whereas other features are shared by the neuroendocrine carcinoma group as a whole, irrespective of anatomic site.

This review concentrates solely on NECs of the GEP-system. A rise in the incidence of these carcinomas has been observed during the past few decades [1,2,3,4,5]. More worrisome however, is the failure to increase survival over the same time period [1,3]. Patients with NECs have a dire life expectancy, with a median survival rate of 4–16 months, reported in studies with both advanced stage and limited staged NEC, and varying in relation to the primary GEP-site [4,6,7,8,9,10,11,12,13,14,15,16,17,18,19]. The literature available for GEP-NECs has grown and developed during the past 20–30 years, with changes in classifications, grading systems, treatments and understanding of different entities within the group. Though an increase of knowledge is positive, the changes within classification systems and staging have led to confusion and a failure to standardize groups in relation to research. Also, due to the rarity of GEP-NECs, the majority of the literature consists of small studies focusing on a few single aspects of the disease or larger studies where only a few cases of NECs are included in a larger NEN-cohort. Large studies specific for GEP-NECs, describing their epidemiology, clinical presentation, genetic background, histopathology, and treatment are rare.

This article provides a comprehensive review of the literature concerning all aspects of GEP-NECs. First, a general understanding of the basic aspects of GEP-NECs in relation to classification systems, epidemiology, biochemistry, imaging, treatment and survival will be explained. Hereafter, organ-specific summaries providing information on epidemiology, presentation, diagnosis, imagery, histology, treatment and survival will be described. Finally, key points and new research will be discussed. The cohorts used to form the basis of this article can also be found in the supplementary tables.

## 2. Method

References for this review were identified through searches of PubMed and Medline.

In PubMed the following search terms were used: “Neuroendocrine Tumors”, “Carcinoma, Neuroendocrine”, “Carcinoma, Small Cell” and “Carcinoma, Large Cell”. All terms were used in combination with the following specifications: “classification”, “diagnosis”, “drug therapy”, “epidemiology”, “mortality”, “pathology”, “radiography”, “radionuclide imaging”, “radiotherapy”, “secretion”, “surgery”, “therapy” and “urine”. As a group, the mentioned search terms were combined with AND to “Antineoplastic Protocols”, “Gastrointestinal Neoplasms” or “Pancreatic Neoplasms”. The search term “Lung Neoplasms” was excluded.

In Medline, the following search terms were used: “Neuroendocrine Tumor/Tumours/Tumors/Tumours/”, “Neuroendocrine Cancer”, “Poorly Differentiated Neuroendocrine Tumor/Tumour/Tumors/Tumours”, “G3 Tumor/Tumour/Tumors/Tumours”, “Neuroendocrine Carcinoma/s”, “G3 Neuroendocrine Carcinoma”, “Small Cell Carcinoma” and “Large Cell Carcinoma”. All terms were used in combination with the following specifications: “incidence”, “prevalence”, “epidemiology”, “WHO Classification”, “morphology”, “mitotic count”, “Ki-67”, “histology”, “pathology”, “diagnosis”, “location”, “octreotide scintigraphy”, “FDG-PET”, “survival”, “treatment” and “chemotherapy”. As a group, the mentioned search terms were combined with AND to “Extra-pulmonary”, “Gastroenteropancreatic Neoplasm/s”, “Gastroenteropancreatic Cancer”, Gastrointestinal Neoplasm/s”, Gastrointestinal Cancer” or “Endocrine Pancreatic Tumor/Tumour”.

Searches in both databases were limited to humans and the languages English, Danish, Norwegian and Swedish.

**Figure 1:**
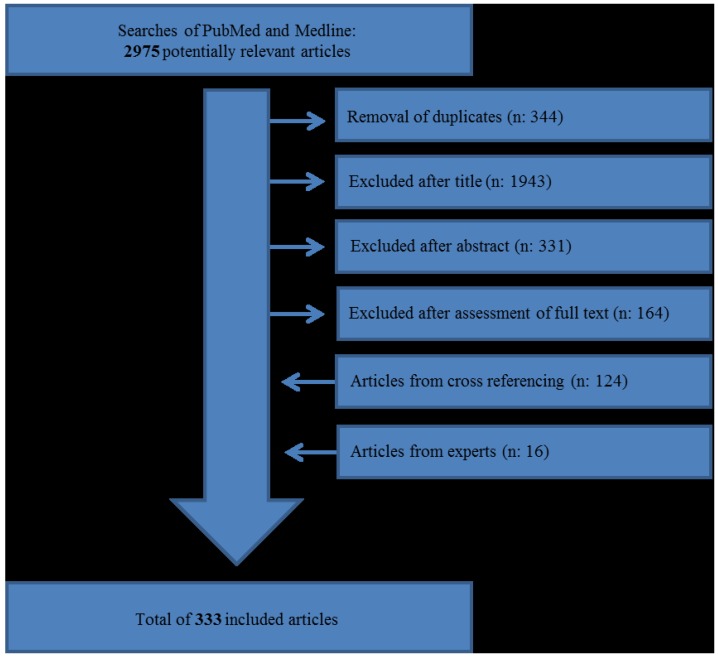
Selection process of relevant articles.

Final searches were conducted on 12 December 2014 and resulted in 2631 articles after the removal of duplicates. Articles were excluded after title (*n* = 1943), after abstract (*n* = 331) and after evaluation of the full text (*n* = 164), resulting in 193 chosen articles. For inclusion, articles were to be specific for GEP-NECs or specific for GEP-NENs with relevance to GEP-NECs. Exclusion criteria were articles without relevance to GEP-NECs, articles not specific to humans, articles written in languages other than those mentioned, case studies with *n* ≤ 5, *in vitro* studies, reviews published before 2010, editorials, letters, commentary, abstracts, highlights from conferences and articles specific for hereditary diseases (MEN-1 *etc.*). Chosen articles were cross-referenced and articles from experts within the field were also evaluated for inclusion, which resulted in a final total of 333 articles, creating the basis of this review (as shown in Figure 1). The final reference list was produced based on the originality and relevance of articles.

## 3. Classification and Histology

The classification of NENs is based on histology. NENs are neoplasms with neuroendocrine differentiation, meaning their cells express various specific neuroendocrine markers such as chromogranin A (CgA) and synaptophysin. Other less specific markers include CD56 and neuron specific enolase (NSE).

Throughout the past 20 years, various classification systems have sub-grouped NENs after their differentiation and grade. Tumour differentiation refers to the extent as to which the neoplastic cells resemble their non-neoplastic counterparts. Tumour grade refers to the tumour’s degree of biological aggressiveness, measured by proliferation.

The World Health Organization (WHO) 2010 classification describes GEP-NEC as a poorly differentiated, high-grade malignant neoplasm, composed of small cells or large to intermediate cells. The grading system currently used for the classification of all GEP-NENs (shown in Table 1) is based on the Ki-67 proliferation index or mitotic count [20], where GEP-NECs should have a Ki-67 index or mitotic count of >20%. If the mitotic count and Ki-67 index should differ, the higher of the two is used.

Grading may hereafter be combined with site-specific tumour-node-metastasis (TNM) staging, referring to the extent of tumour spread. The final diagnosis of a NEN should contain the classification (NET or NEC), the grade (G1, G2, G3), the relevant TNM-stage, and upon request, cell type and functional activity [20].

**Table 1:** World Health Organization (WHO) 2010 Classification.

Neuroendocrine Neoplasm Type	Grade	Ki-67 Index *	Mitotic Count (per 10 HPF **)
Neuroendocrine Tumour (Carcinoid)	G1	≤2%	<2
Neuroendocrine Tumour	G2	3%–20%	2–20
Neuroendocrine Carcinoma	G3	>20%	>20
Mixed adenoneuroendocrine carcinoma (MANEC)	G1–G3 (mostly G3 component)	All ranges	All ranges

* Ki-67 index: % of 500–2000 cells in “hot spot areas” stained positive for MIB-1 antibody; ** 10 HPF: high power field = 2 mm^2^, based on measurement in at least 50 HPFs in hot spot areas.

Though the WHO 2010 classification is widely used, it is still of importance to recognise the earlier classifications of NENs. Due to the large amount of studies prior to 2010, knowledge of previous classifications is required to understand the results of earlier studies and to enable comparisons with more recent literature.

Originally, NENs were classified according to their embryological organ of origin. This separated NENs into the following groups: foregut (thymus, respiratory tract, oesophageal, stomach, pancreatic, duodenal and ovarian), midgut (jejunal, ileal, appendiceal, caecal, ascending colon, and from Meckel’s diverticulum) and hindgut (transverse, descending and sigmoid colon) tumours [21].

In the year 2000, the WHO classification of endocrine tumours was published. NENs were subgrouped as well-differentiated tumours, well-differentiated carcinomas, poorly differentiated carcinomas (PDECs), mixed exocrine-endocrine tumours, and tumour-like lesions [22]. PDECs were described as small cell carcinomas with metastases, angio- and perineural invasion, and proliferation rates of >10 mitoses/10 HPF or a Ki-67 index of >15%. This classification combined both grading and staging parameters. Due to the difference in proliferation levels and the omission of large cell NECs, the WHO 2000 definition of PDECs is not synonymous with the WHO 2010 version of NECs. However, in the WHO 2000 classification of tumours of the digestive system, large cell carcinomas were mentioned under the stomach, colon and rectum sections, but no other classification criteria were described [23].

The classification of pancreatic NENs was reassessed in 2004. PDECs of the pancreas were defined as poorly differentiated carcinomas with small to intermediate sized cells showing neuroendocrine differentiation. The proliferation rate was >10 mitoses/10 HPFs and the Ki-67 index was described as consistently above 10% [24]. Again, large cell carcinomas were not included and the proliferation rate was lower than in the latest classification. The 2004 version of pancreatic PDECs is therefore not synonymous with the WHO 2010 definition of pancreatic NECs.

In 2006, the European Neuroendocrine Tumour Society (ENETS) proposed a combined TNM staging and grading system for NENs based on the Ki-67 index and mitotic count. This grading system is now incorporated in the latest WHO classification system and has been mentioned earlier in the text. It is important to remember that previous classification systems are not synonymous with the current WHO 2010 classification. A study from Lee *et al.* [25] based on colorectal NECs found that when NENs were re-evaluated after the WHO 2010 classification, several NENs received a different diagnosis in relation to grading (e.g., several well-differentiated endocrine carcinomas became NECs). Also, the 2010 classification had a better prognostic value than the WHO 2000 classification. However, another study by Ozkara *et al.* [26] found no difference in the 2004 and 2010 classifications.

The 2010 WHO classification, which is widely accepted, mentions both differentiation (poor differentiation) and grade (high grade, G3) when defining GEP-NECs [20]. However, there is controversy as to whether all high grade neoplasms are also poorly differentiated or not. Some would argue that it is possible for a well-differentiated tumour to have a Ki-67 index over 20%, whereas others would not, or find this unimportant [27,28]. A number of pathologists find the term “poorly differentiated” to be inadequately defined and use only the Ki-67 index for classification if the basic neuroendocrine immunochemistry is correct.

Different nomenclature has been used to describe NECs with a non-neuroendocrine component. Several studies chose to use the term “combined NEC” [29] or “composite NEC”; “combined” when the components are topographically separated and “composite” when there is an intimate admixture of the two.

### 3.1. Small and Large Cell NECs

NECs can be of small or large/intermediate cell type. Both may resemble a poorly differentiated tumour of any kind, which is why testing for cytokeratin is important to establish if the tumour is a carcinoma.

Large cell NECs may show an organoid pattern with solid nests, rosette formations or acinar structures, focal necrosis and high mitotic rate. They have a low nucleus-to-cytoplasm ratio, nuclei with evident nucleoli and vesicular chromatin, and often abundant eosinophilic cytoplasm. As a rule, synaptophysin is diffusely positive while CgA can be frequently negative. Large cell NECs often closely resemble poorly differentiated (adeno)-carcinomas, which is why testing for neuroendocrine markers is of great importance [30].

Small cell NECs are indistinguishable from their counterpart in the lung. Cells are most often small with dark nuclei of round or oval shape and scanty cytoplasm but may be slightly larger with more cytoplasm, forming solid sheets and nests. Staining for synaptophysin is positive in small cell NECs; however, staining for CgA can be negative. According to some, neuroendocrine staining is not obligatory for the diagnosis of small cell NECs because of their typical morphology [31]. However, small cell NECs can mimic malignant lymphoma, synovial sarcoma, PNET and other rare tumours. It is therefore wise to always perform a cytokeratin and synaptophysin staining to be certain of their diagnosis.

Shia *et al.* [31], in a study of 65 GEP-NECs, found no difference in survival between small cell and large cell NECs. However, a large study by Korse *et al.* [3] found the five-year relative survival to be only 6% for small cell NECs (95% confidence interval (CI) 4%–9%) in comparison to 32% in large cell NECs (95% CI 28%–37%). Shia *et al.* [31] emphasize that there is still insufficient data on this topic, and there may be differences between small and large cell NECs in response to treatment. In the same study, Shia *et al.* [31] found the relative frequency of small cell NECs to be higher in the oesophagus and anal canal, whereas the frequency of large cell NECs was higher in glandular mucosa-lined sites of the GI-tract. This finding is supported by Korse *et al.* [3], who found a greater incidence of large cell NEC in the pancreas, as well as the large and small bowel, but a greater incidence of small cell NEC in oesophageal NECs. Also, the Nordic NEC study observed 75% and 65% of oesophageal and rectal NECs to have small cell morphology respectively while only 30% of colonic NEC had small cell morphology [4].

Both small cell and large cell NECs can have a non-neuroendocrine component. Small cell NECs often have an undifferentiated squamous carcinoma component and large cell NECs often have an adenoma or adenocarcinoma component [31]. If both the adenocarcinoma and the neuroendocrine component exceed 30% of the tumour, it is then classified as a mixed adenoneuroendocrine carcinoma (MANEC) [20]. If however, the neuroendocrine component is less than 30% of the tumour, the tumour is then defined as an adenocarcinoma with neuroendocrine differentiation.

### 3.2. Immunohistochemistry (IHC)

For the diagnosis of NEC it is obligatory to test for neuroendocrine markers. CgA and synaptophysin are the most specific neuroendocrine markers [32]. However, owing to the often poor CgA immunoreactivity of GEP-NECs and the ideal situation of immunoreactivity for two neuroendocrine markers, the use of less specific general neuroendocrine markers (NSE, protein-gene-product 9.5 (PGP 9.5) or CD56 N-CAM) may be necessary in combination with testing for CgA and synaptophysin [33]. Some pathologists alternatively diagnose small cell NECs based solely on their morphology. This is however not recommended due to the risk of misdiagnosis of other rare tumours.

CgA is a part of the membrane of neurosecretory hormone granules; therefore, evidence of CgA is dependent on the number of neurosecretory vesicles per cell and GEP-NECs tend to have very few. Synaptophysin, however, is a peptide of the small synaptic vesicles present in all neuroendocrine cells, independent of the number of large neurosecretory vesicles, and is positive in almost all GEP-NECs.

A study from Li *et al.* [34] found 61.9% of small cell GEP-NECs to stain positive for CgA, whereas all GEP-NECs were positive for synaptophysin, and NSE. Another 90.5% were positive for CD56, and only 21.4% for thyroid transcription factor 1 (TTF-1), though oesophageal NECs had a 45.5% positivity for TTF-1.

Thyroid transcription factor 1 belongs to a family homeodomain transcription factors that shows restricted expression in the thyroid, lung and certain regions of the brain. TTF-1 positivity is frequently found in non-squamous carcinomas, small cell carcinomas, large cell carcinomas, as well as typical and atypical carcinoids of the lung. Cheuk *et al.* [35] showed that cytokeratin 20 (CK-20) and TTF-1 can be used to differentiate between small cell carcinoma and Merkel cell carcinoma, due to Merkel cell carcinomas always being CK-20 positive and TTF-1 negative. However, these immunostainings cannot be used for differentiating between GEP- and pulmonary NECs as approximately 21%–53% of GEP-NECs are positive for TTF-1 [34,35,36], in comparison to 83%–96% of small cell lung cancers (SCLCs) [35,36]. Ordónez *et al.* [36] observed that no small cell GEP-NECs were positive for CK20.

A study from Uccella *et al.* [37] found that a DOPA decarboxylase (a key enzyme in the biosynthesis of dopamine and catecholamines) and vesicular monoamine transporter 2 (v-MAT2; mediates the storage of histamine) could be considered as general endocrine markers. However, GEP-NECs had a lower percentage of expression of these markers (60%–77%) than pulmonary NECs and GEP-NETs (especially gastric ECL-cell NETs).

### 3.3. Genetic Profile

The molecular abnormalities of GEP-NECs are not fully established. Generally, NECs have a high level of chromosome instability [38] and exhibit very complex allelotypes with allelic imbalance involving extensive chromosome regions or entire chromosomes, causing NECs to have a significantly higher percentage of allelic gains or losses in comparison to NETs [39]. These different allelic imbalances result in two distinct molecular profiles, supporting the hypothesis of two different genetic pathways in GEP-NETs’ and GEP-NECs’ tumourigenesis [39].

Various studies of GEP-NECs have found abnormalities in the tumour suppressor protein p53 [38,40,41,42,43], concluding that p53 is involved in the pathogenesis of GEP-NECs, most likely its early stages [43]. The p53 protein, a tumour suppressor, has a crucial role in maintaining genetic stability and preventing cancer formation by cell cycle arrest; inducing cell repair or apoptosis, amongst other mechanisms. The exact mechanism involved in relation to GEP-NECs is not established, though involvement of the protein is almost certain. Abnormalities in p53 can be assessed by genotyping of genomic regions and evaluating allelic imbalance (loss of heterozygosity) or by immunohistochemical staining. Overexpression of p53 seen by immunohistochemical staining is generally regarded as a marker of genetic events [44]. Normal p53 cannot be detected by routine immunohistochemistry due to it being present in such small quantities. Conversely, mutant (abnormal) p53 has a longer half-life and builds up in cells to immunohistochemically detectable levels; meaning overexpression of p53 indicates a large amount of p53 protein that is mutant and could therefore contribute to tumourigenesis. If however, p53 mutations were generated by stop codons or frameshifts, or p53 was inactivated by epigenetic silencing, these abnormalities would probably be missed by immunohistochemical techniques. Abnormalities of p53 may indicate disruption of the p53/BAX apoptotic pathway, making cells more resistant to various death stimuli.

A study from Pizzi *et al.* [38] showed that 13/19 PDECs (“poorly differentiated endocrine carcinomas” from the WHO 2000 classification) had an overexpression of p53, implicating a mutation of the protein. Also included in the study were nine well-differentiated endocrine carcinomas; five of these well-differentiated endocrine carcinomas had a Ki-67-index >20%, which according to many today would make them NECs. Interestingly, only 1/5 of these tumours had an overexpression of p53.

The apoptosis inhibitor survivin is negatively regulated by wild-type p53 and upregulated at the G2/M-phase of the cell cycle. In a study by Grabowski *et al.* [45], all included GEP-NECs had nuclear expression of survivin, whereas no GEP-NETs had nuclear expression. Survivin was shown to be predictive of survival and the authors argued that survivin could be more specific for proliferation than the Ki-67 index. The Ki-67 antigen is expressed in every phase of the cell cycle and if the cycle becomes prolonged by G1/S arrest, more mitoses may be visible without necessarily reflecting an accelerated growth rate.

mTOR (mammalian target of rapamycin) is often expressed in NETs, hence the use of everolimus (a mTOR-inhibitor) in NET treatment. A study from Shida *et al.* [46] found 6/9 NECs to have mTOR expression, with the highest expression in large cell NECs. Similarly, Catena *et al.* [47] found 80% of NECs to have mTOR expression, with no association to Ki-67 index or primary site. This could indicate use of mTOR-inhibitors in selected NEC patients.

Overexpression of the tumour suppressor protein p16 has also been observed in GEP-NECs [41,42]. This can lead to dysfunction in the p16/cyclin D1/Rb pathway, causing more cells to progress from G1 phase to S phase in the cell cycle resulting in increased proliferation. Another tumour suppressor, p27, regulates G0 to S phase transitions and low levels are associated with poor prognosis in a variety of cancers. Grabowski *et al.* [48] have shown that GEP-NECs have low levels of p27.

Thymidylate synthase (TS) is an enzyme that plays an important role in cellular proliferation and DNA synthesis. 5-fluorouracil, a chemotherapeutic agent, works by inhibiting TS and DNA synthesis. Higher levels of TS are associated with poorer prognosis and/or adverse responses to 5-fluorouracil treatment in several cancer types. Ceppi *et al.* [49] found levels of TS to increase from NETs to NECs; however, TS was not associated with survival when neoplasms were grouped after tumour grade. Lee *et al.* [50] also found a higher percentage of positive TS tumours within the NEC group in comparison to the NET group, and TS expression was positively correlated with the KI-67 index and worse patient outcome.

Epithelial-mesenchymal transition is seen during embryonic development or tissue fibrosis, as well as in the carcinogenic process, prior to local invasion. In relation to epithelial-mesenchymal transition, GEP-NECs have been shown to have lowered β-catenin expression and raised Foxc2 and Snail-1 expression in comparison to NETs [51]. Also, weak β-catenin and N-cadherin were more common in small cell NECs than large cell NECs.

Human achaete-scute homologue 1 (hASH1), a transcription factor known to play a role in neuronal/endocrine differentiation, was found in the majority of NECs (9/10) in a study by Shida *et al.* [52]. However, in composite NECs, hASH1 was found only in the neuroendocrine component, indicating that hASH1 is possibly involved in the differentiation of NECs from pluripotent cells [52]. Another transcription factor, NeuroD, was shown to be associated with survival in NECs with better survival in NECs with high NeuroD expression in comparison to those with low NeuroD expression [53].

Other molecular abnormalities found in GEP-NECs are allelic imbalances of the proto-oncogene l-myc [41]. Also, HIF-1α, CA9 and Akt are all correlated with tumour grade or differentiation in a study from O’Toole *et al.* [40]. The stem cell marker CD133 is not expressed in the normal neuroendocrine cells of the GEP-system, however, expression has been found in both GEP-NECs and GEP-NETs [54]. The significance of this is uncertain, as no prognostic difference was found between CD133 positive and CD133 negative NENs [54].

C-kit (also known as CD117) is a proto-oncogene that encodes a transmembrane tyrosine kinase receptor involved in cellular differentiation. C-kit is overexpressed at a relatively high frequency in pulmonary NECs [42]. However, overexpression of c-kit is rarer in GEP-NECs, having been observed in a seldom 0%–30% of GEP-NECs [42,55,56]. This indicates that GEP-NECs are less likely to respond to c-kit tyrosine kinase inhibitors. A Japanese study [56] found that amongst the 26% of GEP-NECs that had an overexpression of c-kit, none had a mutation at exon 9–13; the hot spot of the mutation of c-kit in gastrointestinal stromal tumours. Interestingly, a study from Knösel *et al.* [55] found that the few GEP-NECs with an overexpression of c-kit were associated with a worse survival than those without an overexpression. PDGFRA (alpha-type platelet-derived growth factor receptor) overexpression was also associated with worse survival in the same study.

RASSFIA (Ras-ASSociation domain gene Family 1A) is a tumour suppressor gene known to induce cell cycle arrest in different cancers. However, inactivation of this gene does not play a major role in GEP-NEC tumourigenesis [57].

A study from Furlan *et al.* [43] found allelic imbalances in gastric and colorectal-NECs similar to those known in exocrine tumourigenesis of similar sites. This supports the hypothesis that NECs and exocrine carcinomas share early pathogenic pathways. However, there are several mutations common in exocrine carcinomas that are not found in GEP-NECs. For example, K-ras mutations are rare [41]. Also, transcription factor CDX2 has been suggested to be a marker for colorectal adenocarcinomas and could possibly therefore be a marker for intestinal origin. However, La Rosa *et al.* [58] showed that only 11% of GEP-NECs showed nuclear CDX2 immunoreactivity. CDX2 immunoreactivity seemed to be mainly restricted to GEP-NETs, in particular midgut EC-cell tumours [58].

## 4. Origin

NENs were previously thought to originate from cells of the neural crest. The current interpretation of the origin of NENs is that they derive from multi-potent stem cells, which due to genetic alterations (inherited or acquired mutations) progress into cancer. On the basis of their genetic programme and mutations, these multi-potent cells may differentiate into epithelial, glandular, or neuroendocrine cancer type cells and may stop their distinct differentiation programmes at different stages of maturation [59,60]. This is supported by Brenner *et al.* [7], amongst others, as described in the organ-specific sections. It is important to remember that GEP-NECs and GEP-NETs are genetically [60] and clinically distinct.

Another hypothesis for the origin of GEP-NECs has been that GEP-NETs gradually progress into GEP-NECs. La Rosa *et al.* [61] in a study of gastric NENs have described a progression of G1 gastric NETs to G3 NECs, suggesting that the origin of some high grade NECs is possibly from their low/intermediate grade counterparts. Though this cannot be entirely excluded, the concept of neoplastic progression is probably rare and does not represent the vast majority of NECs [27,60,62,63]. Rindi *et al.* [63] argue that a separate origin for NETs and NECs is most probable, supported by the absence of non-neuroendocrine components in well-differentiated NENs [64].

## 5. Epidemiology

A rise in the incidence of GEP-NENs has been established [19]; however, the question is whether NECs have also risen in incidence during the past few decades. A study from Wong *et al.* [65], found no increase in the incidence of extra-pulmonary small cell carcinomas (33% from the GI-tract) between the years 1970–2004. However, a study based on the Netherlands’ Cancer Registry found a trend of increased incidence of NECs of the stomach, small bowel, large bowel, pancreas and oesophagus between the two periods of 1990–2000 and 2001–2010, especially of large cell NECs [3]. The Nordic NEC study also found far more registered patients in the period of 2005–2009 than 2000–2004 [4]. Correspondingly, a registry study of the population of England and Wales found a 50% increase of small cell GEP-NECs between the years 1986–1999 [1], and Cho *et al.* [2] found that the incidence of NECs in Korean hospitals had increased between year 2000–2009. Interestingly, the study from England and Wales also found the incidence of small cell GEP-NECs to be twice as high in the most deprived social class in comparison to the most affluent. A SEER study has also observed an annual increase of 9.8% in the incidence of colorectal NECs during the period of 1991–2000 [5].

GEP-NECs compromise less than 1% of gastrointestinal cancers [18] (approximately 0.26%–0.28% [1,66]) and 0.017% of invasive cancers [12]. In a study from Niederle *et al.* [66], the yearly incidence of poorly differentiated carcinomas was approximately 0.14/100,000 people. In relation to all GEP-NENs, GEP-NECs constitute 5.6%–25.6%, with a fluctuating frequency over time [1,2,3,8,18,26,40,44,45,47,50,51,54,55,66,67,68,69,70,71,72,73,74].

Many epidemiological studies have examined small cell or large cell NECs separately and compared for example GEP small cell NECs with other extra-pulmonary small cell carcinomas. Small cell GEP-NECs represent 13%–55.7% of extra-pulmonary small cell carcinomas [3,6,9,10,11,12,13,15,16,17,65,75,76,77,78]. A study based on the EUROCARE database found that the proportion of small cell NECs varied from 3.4% in Norway and Iceland to 30.3% of all malignant NENs in the UK, showing that incidences can vary depending on geographical region [79].

In several studies, the small cell or large cell groups of all extra-pulmonary organs have been organized as one large group. This has its drawbacks, as tumour progression and survival differ depending on the given extra-pulmonary site, with GEP-NECs often having the worst prognosis in comparison to small cell carcinomas of other sites such as the gynaecological/genitourinary system and the breast [6,9,11,12,13,15,17,65].

GEP-NECs are most often found in the pancreas, colorectal region, stomach or oesophagus, whereas NECs of the small intestine, gallbladder, appendix or liver are very rare [1,3,6,9,11,12,13,14,15,31,34,35,36,38,41,53,54,58,66,67,68,70,72,73,74,75,80,81,82,83,84,85,86,87,88,89,90,91,92]. It is important to mention that large and small cell carcinomas of unknown primary sites constitute a large portion of GEP-NEC cases. In a study from Korse *et al.* [3] including pulmonary NECs, 20% of large cell NECs and 5% of small cell NECs had unknown primaries. Also, in the Nordic NEC study, 32% of patients had an unknown primary [4].

## 6. Biochemistry

Generally, NENs can either be functioning or non-functioning, with functioning neoplasms secreting such high levels of a certain hormone that a hormone specific syndrome is induced. Non-functional neoplasms can still produce hormones but not be functional due to the produced hormone not being secreted, the level of secretion not being high enough, or that the hormone when secreted does not produce a syndrome. GEP-NECs are seldom functioning neoplasms.

Certain biomarkers do not cause a syndrome but can be monitored in both functional and non-functional NENs in relation to progression and treatment response. The main biochemical marker in NENs is chromogranin A (CgA), a glycoprotein with a diverse array of biofunctions.

Despite efficient monitoring in GEP-NETs, CgA is not always of great value for diagnosis and monitoring in GEP-NECs. Cimitan *et al.* [80] showed that only 4/9 GEP-NEC had raised CgA levels and the sensitivity of CgA as a biomarker was 37%. However, in the Nordic NEC study, 66% of patients had elevated CgA serum levels when measured, and 45% had levels over twice the upper normal limit [4]. Due to this finding, the Nordic guidelines suggest that CgA levels should be measured in GEP-NECs [30]. Measurement of U-5HIAA (serotonin metabolite) is not recommended [30].

Other biochemical markers such as progastrin-releasing peptide (pro-GRP) and cytokeratin fragments (CKfr, CK8, 18, 19) may be of value in patients with GEP-NECs [93]. Korse *et al.* [93] showed that median levels of pro-GRP, NSE and CKfr were higher in patients with NECs than patients with NETs. However, the majority of NECs were of pulmonary origin, with only 15/293 patients having a GEP-NEC. The same study also showed that 67% of large cell NECs had a CgA level over the upper limit of normal (similar to GEP-NETs), whereas only 36% and 17% had levels over the upper limit of normal of NSE and proGRP, respectively. Small cell NECs appeared to have a lower level of CgA than large cell NECs but due to the large number of SCLCs and only two small cell GEP-NECs, results were difficult to interpret. In a cohort of 294 NENs, 28 had elevated alpha-fetoprotein levels. Of these 28 neoplasms, the median Ki-67 index level was 21%. Rising alpha-fetoprotein levels were correlated with the Ki-67 index and lower survival. The authors suggested that alpha-fetoprotein could be a biomarker of dedifferentiation, and therefore possibly NECs [94].

In a study of oesophageal NECs, baseline serum neuron-specific enolase (NSE) was found to correlate to chemotherapy response and overall survival. Patients with lower baseline serum-NSE (≤17 ng/mL *versus* >17 ng/mL s-NSE) were found to respond better to chemotherapy treatment and had a higher survival rate than those with a higher baseline serum-NSE [95]. It is unknown whether this can be extrapolated to GEP-NECs of other sites, though Korse *et al.* [93] suggested that NSE may exert higher diagnostic use than CgA.

In conclusion, the recommended biomarker to monitor is CgA. However, serum NSE could prove to be useful in evaluating tumour aggressiveness and treatment response, but further studies are needed before testing for serum NSE levels becomes routine.

## 7. Ki-67 Index

Ki-67 is a nuclear protein expressed in proliferating cells (*i.e.*, during the G1, S, G2 and M phases of the cell cycle and not the G0 phase). Expression of Ki-67 is assessed by staining with an MIB-1 antibody which binds to the Ki-67 antigen. The Ki-67 proliferation index is 100 times the number of positively staining tumour cells, divided by the total number of tumour cells. The Ki-67 index should be assessed in areas where the highest nuclear labelling is observed (in “hot spot areas”), in a sample of 2000 cells. Ideally, 20 areas of 100 cells with the highest nuclear labelling are assessed, however, if this is not possible, the actual number of hot spot areas assessed is noted.

Different methods are used to assess the Ki-67 index; eyeballing (judging an approximate percentage by scanning the slide and not formally counting), computer programs, or manual cell counting. NECs are often easily distinguished from lower grade tumours. Generally, manual counting is more accurate, being comparable to digital image analysis, whereas eyeballing is less accurate but also less time consuming [96,97].

The Nordic NEC study [4] found that the average Ki-67 index can differ between GEP-NEC sites. In oesophageal, rectal and colonic NECs 67%–80% had a Ki-67 index over 55%, whereas only 30% of pancreatic NECs had a Ki-67 index over 55%. The same study found no difference in Ki-67 indexes between small cell and non-small cell NECs [4].

The Ki-67-index can also vary between the primary tumour and its metastases, as well as within the individual tumours themselves. Generally, the Ki-67 index is higher in metastases than in the primary, as shown in two separate studies from Hentic *et al.* [98] and Delektorskaya *et al.* [67]. The Ki-67-index may also increase or decrease in response to treatment. It is always the highest Ki-67 index value that decides the tumour grade.

The use of mitotic count *versus* Ki-67 index is controversial. However, when dealing with biopsies, it might not be possible to perform an accurate mitotic count due to the recommendation of counting 40–50 high-power fields, which is more than what most biopsy samples contain. In this case, the Ki-67 index is superior to the mitotic count in separating NECs from NETs [27].

## 8. Imaging

Imaging techniques have greatly improved during the past few decades, with both CT and MRI having become more accessible and with better resolution. According to ENETS, staging of GEP-NECs should include CT of the chest-abdomen-pelvis [99]. The North American Neuroendocrine Tumour Society (NANETS) guidelines add that CT, MRI and 18F-fluorodeoxyglucose (FDG)-positron emission tomography (PET) may also be used for baseline staging and monitoring response to treatment [100]. A review of imaging techniques found that MRI is best for imaging of liver metastases in NENs [101]; however, for other metastases CT is usually used. CT-scans should be multiphase contrast-enhanced. On CT-scans GEP-NECs are often hypo-attenuated [102], in contrast to NETs which are often hyper-attenuated.

Functional imaging within NENs includes imaging techniques such as FDG-PET and somatostatin receptor imaging (SRI). SRI may either be performed as SPECT with ^111^In or ^99^Tc labelled somatostatin analogues or, especially in Europe, more and more as PET with ^68^Ga or ^64^Cu labelled ligands. Compared to SPECT, PET has shorter scan protocols, better spatial resolution (resulting in higher diagnostic sensitivity) and allows for absolute quantification (which is valuable for treatment planning and monitoring). The most commonly used tracers for PET SRI are ^68^Ga-DOTATOC, ^68^Ga-DOTATATE and ^68^Ga-DOTANOC. At large, there is no major difference between these three tracers [103]. Recently, ^64^Cu-DOTATATE has been introduced as a promising SRI-PET tracer [104]. Advantages of using ^64^Cu instead of ^68^Ga as isotope includes; shorter positron range (giving better spatial resolution), a longer half-life (allowing delayed imaging) and a shelf-life of 24 h.

To date, five subtypes of somatostatin receptors have been identified (SSTR1-5), with SSTR2 further classified into subtypes 2A and 2B. Membranous expression of SSTR2A can be demonstrated by immunohistochemistry, as well as by PCR [72,105], and is correlated with SRI [69,106]. It should be noted that the current ligands used for SRI all have the highest affinity for SSTR2 receptors [103].

There is an ongoing debate as to whether GEP-NECs have somatostatin receptor expression and if SRI can be of use in these patients. Papotti *et al.* [72] found that GEP-NECs appeared to have a lower expression of SSTRs than GEP-NETs; however, the study only included five NECs. Of these five, three had expression of only one or none SSTRs, in comparison to three different SSTR types being present in 90% of the whole gastrointestinal cohort. Conversely, Fjällskog *et al.* [69] found no difference in the distribution of SSTR expression between NECs and NETs. The five included poorly differentiated carcinomas showed 100% positivity for SSTRs 1 and 4, 80% positivity for SSTRs 2 and 3, but only 40% positivity for SSTR5. Other studies have also observed expression of SSTR 2 in 67%–100% of included NECs, however expression of SSTR 5 is often low (17%–50%) [74,106].

When evaluating SRI, NETs are generally more positive than NECs, with an increasing Ki-67-index often meaning a decrease in the number of SRI-positive tumours [107]. Despite this, it seems that a number of NECs are actually positive. Studies have shown that 37%–71% of included GEP-NECs are positive on somatostatin receptor scintigraphy (SRS) scans [4,69,74,80,87,90,106,108,109], and in a study from Binderup *et al.* [107], 9/13 patients with a Ki-67 index >15% also had a positive SRS while 12/13 had a positive FDG-PET. However, the question is if a positive SRI is of any actual clinical value. In relation to treatment with peptide receptor radionuclide therapy (PRRT), a study by Ezziddin *et al.* [110] has shown that NECs have a poorer response to PRRT despite avid receptor-mediated tracer uptake. In a French study, 10/14 GEP-NECs had a positive octreotide scan and most of these GEP-NECs were well-differentiated [90], again posing the question of whether well-differentiated NECs are a separate entity to poorly differentiated NECs. Despite a general correlation between somatostatin receptor expression in immunohistochemistry and SRI, Sclafani *et al.* [74] did not recommend the use of immunohistochemistry instead of SRI. This is due to the number of false negative immunohistochemistry stainings in their cohort, explained by heterogeneous intratumoural SSTR expression.

Another modality of increased use in functional imaging is FDG-PET. In a retrospective study, Gregory *et al.* [77] showed that FDG-PET was more informative than conventional CT/MRI when diagnosing small cell NECs, with a positive predictive value of 100% at diagnosis, and 82% for residual disease. PET influenced a change of treatment choice in 3/7 patients after their PET-scan, and after treatment a new PET-scan influenced a change in future treatment management in 2/7 patients. However, one patient had a false positive result from FDG-PET after treatment, which may signify difficulties with interpretation of post-treatment change on FDG-PET. The sensitivity of FDG-PET was 100% and the specificity 83%. However, 50% of those who received a complete response on FDG-PET still died of disease progression. In agreement with Gregory *et al.*, Oh *et al.* [111] found that 90% of lesions could be found with FDG-PET in patients with GEP-NECs.

Binderup *et al.* [112] found that NENs with a Ki-67 >15% were often positive on FDG-PET (13/14 patients), and that patients with a SUV max >3 had a shorter progression free survival. The same study showed that patients with a lower Ki-67 index and a positive FDG-PET often died earlier than those with a negative FDG-PET, implying that FDG-PET could be of prognostic value. Indeed, in a multivariate analysis including both FDG-PET positivity and the Ki-67 index, only FDG-PET remained significant. One explanation for the strong prognostic power of FDG-PET could be that whole body imaging is not prone to sampling error in contrast to the Ki-67 index which is reliant on few biopsies for its assessment.

Other imaging modalities such as ultra-sound (US) and endoscopy are also used in GEP-NECs. US can be used for assessing solid viscera, or to take an ultra-sound guided biopsy, with contrast enhanced US giving additional information. Endoscopic ultra-sound (EUS) can be used to position the probe much closer to the organ than regular US and now novel devices also allow for core biopsies to be taken in the pancreas, whereas only fine needle aspiration was previously possible [113].

## 9. Treatment

The treatment of GEP-NECs is not straightforward and requires a multidisciplinary approach. When possible, surgery has always been advocated as the sole possibility of cure. However, the majority of patients are treated with chemotherapy due to most having disseminated disease at diagnosis. Somatostatin analogues are not recommended for NECs according to several guidelines [30,99,114], as they have only been documented to have an anti-tumoural effect in NETs with a Ki-67-index below 10% and as patients with NECs very rarely have hormone induced symptoms (for which somatostatin analogues can be effective).

Here, we present generalized studies concerning the treatment of GEP-NECs. Under each organ section, there is also specific treatment information regarding the organ in question.

### 9.1. Surgery

The role of surgery in GEP-NEC is controversial and can differ in relation to the anatomical site of the primary. The concept of surgery being the only possibility of a cure is not entirely true. There have been several cases of long-term survival from radiation and chemotherapy, for example when treating oesophageal, limited-stage NECs. Due to the higher risks of mortality combined with surgery, as well as the high percentage of swift recurrence, it is not always clear whether surgery is of benefit.

Brennan *et al.* [6] do not recommend surgery, even for limited disease, after evaluating 120 mixed location small cell extra-pulmonary carcinomas. However, a later study from Li *et al.* [16] with a similar patient group, recommends a combination of local therapy (surgery or radiotherapy) and systemic therapy (chemotherapy) for limited stage disease. The same study [16] found that surgery and chemotherapy prolonged survival time significantly in small cell NECs and that surgery specifically prolonged the survival time in limited stage, oesophageal small cell NECs. Another study from Brenner *et al.* [7], specific for small cell GI-NECs, supports the conclusions of Li *et al.*, finding it reasonable to treat patients with pre- and post-surgery chemotherapy, and suggested a potential role for surgery in limited disease, small cell NEC.

There is general consensus that surgery is considered in cases of limited disease, and if performed should be combined with adjuvant chemotherapy due to the risk of metastatic spread and recurrence. Guidelines recommend adjuvant therapy of cisplatin/carboplatin and etoposide [30,100]. Obviously, surgery is also considered in prevention or elimination of obstructive NECs.

### 9.2. Chemotherapy

First-line chemotherapy for GEP-NECs is cisplatin or carboplatin combined with etoposide [30,99,114,115]. It is essential that patients with metastatic NECs start chemotherapy as soon as possible, before their performance status declines to the extent that they are no longer fit for chemotherapy [30]. Chemotherapy is generally seen as a method to increase survival time while maintaining the best possible quality of life. Chances of a cure are very slim and responses to therapy are often short-lived [15].

The first-line therapy regime of cisplatin and etoposide is based on studies from 1991–2001 by Moertel *et al.* [116], Mitry *et al.* [88] and Fjällskog *et al.* [83]. This treatment combination, extrapolated from the treatment of SCLC, gave response rates of 41.5%–67% and progression free survivals (PFSs) of 7–11 months. Etoposide was first given in doses of 130 mg/m^2^, but due to severe haematological and neuropathic side-effects the dose was lowered to 100 mg/m^2^. More recent studies [90,98,117,118] have shown lower response rates of 17%–37.5% from the same treatment. This is probably due to changes in the classifications of NECs and response criteria, as well as the development of better imaging causing earlier detection of progression.

Carboplatin has been tested as an alternative to cisplatin, due to less nephrotoxicity, neurotoxicity and more convenient administration. Hainsworth *et al.* [84] have tried a regime of carboplatin, etoposide, paclitaxel, followed by weekly paclitaxel after four courses. The subsequent weekly paclitaxel had doubtful value, while the first regime of carboplatin, etoposide and paclitaxel gave an overall response rate of 53% and median PFS of 7.5 months. However, at least 82% of patients developed grade 3 or 4 toxicity which is why this regime has not been broadly used. This study was composed of only 15 GEP-NEC in a cohort of 78, including 48 with an unknown primary. Olsen *et al.* [119] have also tried first line treatment with a combination of carboplatin and etoposide, but replacing the paclitaxel with vincristine. This gave a response rate of 52% and PFS of 6.6 months in a mixed cohort. A study from Germany has compared the two regimes of cisplatin and etoposide *versus* carboplatin, paclitaxel and etoposide, finding no significant difference in treatment outcome [118]. The Nordic NEC study has since suggested that cisplatin can be replaced by carboplatin as the two are comparable in efficiency, and addition of a third drug (vincristine) did not affect efficacy [4].

Correale *et al.* [59] have tried combining etoposide and cisplatin with slow release lanreotide. The hypothesis was that lanreotide could sensitise tumour cells to cytotoxic drugs between cycles of chemotherapy, delay the recovery of less chemo-sensitive cells, and synchronise the cell cycle of tumour cells making them more vulnerable to cycle-specific cytotoxic drugs. The treatment produced an objective response rate of 37% and a high rate of disease control. However, 63% (17/27) of included patients had primary sites outside of the GEP-system. At present, we have no substantial evidence that a somatostatin analogue should be added to chemotherapy in GEP-NECs.

A number of Asian studies have also tested replacing etoposide with irinotecan, with response rates varying from 34%–67%, and PFS from 4.5–5.5 months [86,87,92,117,120]. Grade 3–4 side effects were often haematological and affected 60%–71% of patients [86,87,120]. Cohorts were compromised of NECs of mixed locations [86], and not all had advanced disease [120]. A large retrospective study by Yamaguchi *et al.* [92] found patients to have a better response rate when treated with cisplatin and irinotecan compared with cisplatin and etoposide (39% *versus* 12%), however, the primary tumour sites were not equally distributed within the two groups. The increased use of irinotecan in Asia mimics the tendency in SCLC; Caucasians tend to experience increased gastrointestinal toxicity after irinotecan in comparison to Asian populations [121]; hence, etoposide is generally still the treatment of choice in non-Asian patients with NEC and SCLC.

Other treatment combinations for first-line chemotherapy have been 5-fluorouracil and cisplatin [108]; 5-fluorouracil, cisplatin and streptozocin [122]; 5-fluorouracil and irinotecan [81]; and lastly, oxaliplatin and capecitabine [123,124]. These treatment options have not gained standardized use.

Second-line chemotherapy for GEP-NECs is not established [30]. The Nordic NEC Study suggests reinduction with platinum/etoposide, or possibly the use of temozolomide or capecitabine [4].

Welin *et al.* [109] proved that temozolomide is an option for second-line treatment with an overall response rate of 33% with minimal toxicity in a retrospective mixed cohort (*n* = 25) including 68% GI-NECs. A subset of patients were treated with capecitabine (*n* = 19) or bevacizumab (*n* = 11) in combination with temozolomide. This did not seem to have an additional effect on treatment, however, patient numbers were small and therefore the possible benefit of adding these compounds is unknown. MGMT-promotor methylation analysis (epigenetic silencing is a predictive factor of benefit of temozolomide treatment in patients with glioblastoma) was positive in only 6% of those tested. Patients who had not responded to first-line therapy appeared to respond better to second-line therapy than those who had responded; therefore, lack of response to first-line treatment should not prevent patients from trying temozolomide as second-line treatment. Also, patients with a Ki-67 index <60% responded better to treatment than those over 60%, as well as patients with strong SRS uptake responding better to treatment than those without. Those with a positive staining for CgA were likewise more often responders to treatment [109]. A study from Olsen *et al.* [125] supported the use of temozolomide as an acceptable option for palliative treatment, especially in patients with a Ki-67 index <50%, with 38% of the whole cohort obtaining disease control. These studies indicate that temozolomide may be more efficient in NEC patients with a Ki-67 index below 50%–60%.

In a study by Hentic *et al.* [85], a regime of irinotecan and 5-fluorouracil was given to patients progressing on etoposide-platinum treatment. This resulted in a response rate of 31% and PFS of 4 months. The multi-target tyrosine kinase pazopanib has also been tested as an option for second-line chemotherapy, however, the response rate was only 23% and PFS 5.8 months, suggesting that this treatment should only be considered in selected patients with limited treatment choices after failed first-line therapy [126].

Recently, an article has been published showing the possibility of topotecan as a second-to-fourth line treatment in NECs [127]. The study showed 23% of heavily pre-treated patients obtaining stable disease with tolerable toxicity, however PFS was only 2.1–2.5 months and survival was poor with a median survival time of only 3.2 months.

Unfortunately, it can be difficult to assess and compare the studies of chemotherapy in GEP-NECs. This is due to there being many small retrospective studies from single centres. Also, studies tend to have mixed cohorts; *i.e.*, mixed NECs and NETs, or mixed locations (lungs, GEP-system, urogenital systems *etc.*). Moreover, patients in different studies often differ dramatically in relation to previous treatment, advanced disease, Ki-67 score, performance status and age.

A topic of debate in relation to chemotherapy is whether there is a Ki-67 index cut-off point for response to chemotherapy and survival. Alternatively, a difference in treatment response and survival could lie in relation to tumour differentiation, with a number of NECs (Ki-67 index >20%) being well-differentiated. The Nordic NEC study [4] found that NECs with a Ki-67 index >55% had significantly worse survival but responded better to platinum-based chemotherapy than those with a Ki-67 index <55%. This has been replicated in studies by Olsen *et al.* [125] and Welin *et al.* [109]. However, these studies did not specify whether included NECs were poorly differentiated or not. This could mean that a number of NECs were well-differentiated with a Ki-67 >20%, which would explain a better survival rate and lower response to treatment. A study of pancreatic NECs excluded all well-differentiated tumours with a Ki-67 index >20% and subsequently did not observe any Ki-67 cut-off point for better survival [128]. Another study from France showed that well-differentiated NECs had a median survival time of 41 months compared to the 17 months median survival time of poorly differentiated NECS, though this was not significant due to too smaller numbers [90]. Also, complete and partial responses to cisplatin-based chemotherapy were found only in the poorly differentiated NECs. However, there are as yet no objective definitions of poorly differentiated *versus* well-differentiated NENs; therefore, it is not clear as to how poorly differentiated has been defined in the above mentioned studies.

The first-line treatment of GEP-NECs has largely been based on the treatment of SCLC, however NECs of the GEP-tract should be considered as a separate disease entity. It is uncertain whether the different organ sites of GEP-NECs should also be considered as different entities.

### 9.3. Other Treatment Modalities

Radiotherapy is not considered as a sole treatment for GEP-NECs. However, radiotherapy can be combined with chemotherapy or surgery, especially when treating oesophageal NECs.

Peptide receptor radionuclide therapy (PRRT) is another option rarely used in GEP-NECs. A positive SRI scan is required to show that the carcinoma has receptors that can be targeted by the PRRT treatment. However, a retrospective study from Ezziddin *et al.* [110] showed that PRRT had an increased failure rate in NECs comparison to NENs as a whole, despite avid receptor-mediated tracer uptake.

### 9.4. Metastases

Surgical treatment of metastases is generally not recommended for GEP-NECs [30,99,129].

In relation to brain metastases, prophylactic cranial radiation is not preformed routinely [100,130]. This is due to the lack of knowledge regarding its efficiency in patients with GEP-NECs [75], as well as few NEC patients experiencing brain metastases in comparison to patients with small cell lung cancer [6,7,75,117].

The majority of GEP-NEC patients develop liver metastases. Surgery with curative intent is often not possible, as there are often metastases disseminated in both liver lobes and metastases as isolated bulk with smaller deposits [131]. Frilling *et al.* [131] suggest the use of radio frequency ablation, hepatic artery embolization, chemoembolization or peptide receptor radionuclide therapy (PRRT) instead of surgery for liver metastases in GEP-NECs. Yttrium-90 radioembolization is an option, but best results are observed in patients with NETs, without extra-hepatic disease and low tumour burden [132]. However, this was based on only eight poorly differentiated NECs.

With regard to liver transplantation, allocation of organs to patients with malignancies can only be justified if expected five-year survival is over 50%. A retrospective study and review of the literature by Le Treut *et al.* [133] found that liver transplantation should only be offered to well-differentiated NETs with a Ki-67 <10%. Therefore, liver transplantations are not a treatment option for GEP-NECs [99].

### 9.5. Follow-Up

According to the Nordic and NANETS Guidelines, radically operated patients should be followed every 3–6 months with CT of the thorax and abdomen, as many patients will have a rapid recurrence of the disease [30,134]. Patients treated by chemotherapy should be monitored for progression every 2 months by CT of the thorax and abdomen according to Nordic Guidelines [30] and every 6–12 weeks according to NANETS guidelines [134]. In the European Society for Medical Oncology (ESMO) guidelines, follow up for R0/R1 patients should be every 2–3 months and in patients with non-resectable disease every 3 months [114]. If there is stable disease, ENETS suggests follow up every 6 months [99].

## 10. Survival

Patients with GEP-NEC live a median of 4–15.6 months after their diagnosis, with variations in relation to disease stage and primary site [4,6,7,8,9,10,11,12,13,14,15,16,17,19,40,48,92]. Without treatment survival is merely 1 month [4].

Unfortunately, survival for GEP-NECs does not seem to be improving. Lepage *et al.* [1] showed that the survival for small cell NECs had not improved in the 14-year span observed (1986–1999). Neither did trends of survival seem to improve for GEP-NEC patients between the periods 1990–2000 and 2001–2010 according to the Netherlands’ Cancer Registry [3]. This could be explained by the misclassification of large cell NECs during 1990–2000, before the cancer registry had implemented a specific histology code for large cell NECs.

Factors affecting survival according to the Nordic NEC study were the location of the primary tumour, performance score, blood levels of platelets and levels of lactate dehydrogenase (LDH) [4]. Similarly, Yamaguchi *et al.* [92] found LDH levels and primary site to be independent prognostic factors for overall survival. However, the primary site differed between the two studies: in the Nordic NEC study [4] pancreatic NECs had more favourable survival rates, whereas this was the opposite in the study by Yamaguchi *et al.* [92]. For small cell NECs of the GEP-system, performance status, weight loss, TNM stage, and whether or not the tumour was limited stage disease (LD) or extensive stage disease (ED) were prognostic for survival in the study from Brenner *et al.* [7]. Previously mentioned factors, such as level of differentiation or level of Ki-67 index, can also influence survival. A study from Hentic *et al.* [98] found three well-differentiated NETs (WD-NETs) to have a Ki-67 index >20%, though few in number, these tumours had a worse survival rate than the WD-NETs with a Ki-67 index <20%, and a better survival than PDECs (five-year survival of 33% *versus* 6%).

Survival can vary between different GEP-sites. The specific organ survival is described in the following individual organ sections.

## 11. Oesophagus

Neuroendocrine carcinoma (NEC) of the oesophagus was first described in 1952 by McKeown [135] and since then, almost 4000 cases have been reported in the literature [1,3,4,6,9,13,15,29,35,58,87,92,95,135,136,137,138,139,140,141,142,143,144,145,146,147,148,149,150,151,152,153,154,155,156,157,158,159,160,161,162,163,164,165,166,167,168,169,170,171,172], constituting approximately 1.6% of oesophageal cancers [13,135,136,137,138,139,140,141,143,144,145,146,147,148,149,150,151,152,156,158,159,160,161,162,163,164,165,166,167,168,169,171,173,174,175,176]. Generally, oesophageal NECs are of small cell type (small cell oesophagus cancer; SCEC) [1,6,9,11,13,15,16,29,35,135,136,137,138,139,140,141,142,143,144,145,146,147,150,151,152,154,155,156,158,159,160,161,162,163,164,165,166,167,168,169,170,171,172,174,175,176,177,178], occurring in 50–70-year-old men [16,29,58,95,135,136,137,139,141,143,144,145,146,147,148,150,151,152,153,154,155,156,158,159,160,161,162,163,165,166,167,168,169,170,171,172,174,175,176,177,178,179] with a slightly increased incidence within the Asian population [144,146,156,159,162,171,174,180]. Smoking and excessive alcohol consumption is often seen in patients with SCEC [136,144,145,147,150,154,168,171,175,178,180,181]. Tumours range in size from a few centimetres to 17 cm in length and are most frequently found in the middle and lower parts of the oesophagus [135,137,138,139,142,143,144,146,150,151,152,155,160,165,166,168,169,171,172,174,175,176,178], though tumours of the upper oesophagus also occur [95,135,137,139,143,144,145,146,147,149,150,151,152,153,154,155,156,158,162,163,165,166,168,169,170,172,174,175,176,177,178]. Patients tend to experience dysphagia, weight-loss and pain up to one year before diagnosis [13,135,136,137,138,140,142,144,145,147,149,150,153,154,157,162,163,164,166,167,168,169,170,171,175,178,180]. Lymph node metastases and distant metastases are common at presentation [95,137,138,140,148,150,158,160,161,165,168,177,182], with distant metastases most often located in the liver and distant lymph nodes [136,137,138,140,142,147,149,153,160,161,163,165,168,171,175,177,178,179,183]. Brain metastases are a relatively rarer occurrence in comparison to SCLC, representing only 1.6% of oesophageal NEC metastases [153,171,178].

Endoscopy with biopsy is the main tool for diagnosing SCEC [166,168], with imaging techniques (CT-scan, MRI, *etc.*) aiding in staging the cancer. Various staging systems have been used in the literature, most commonly the Veterans’ Administration Lung Study Group (VALSG) classification with limited or extensive disease [139], the American Joint Committee on Cancer criteria (AJCC) [164], the International Union against Cancer criteria (UICC) [177], or in some Japanese studies; the Japanese Classification of Esophageal Cancer [176]. The WHO Classification of 2010 recommends the AJCC staging system for most oesophageal neoplasms [20]. The 2002 version of this staging system was shown by Wang *et al.* [168] to be more sensitive at predicting survival than the VALSG system.

SCEC is histologically identical to its counterpart in the lung [184]; therefore, excluding a primary SCLC through imaging or bronchoscopy is essential. Immunohistochemical staining for neuroendocrine markers is mandatory for large and small cell NEC to exclude other rare tumours. SCEC and large cell oesophageal NEC often contain a non-neuroendocrine component which can be of a squamous cell type or adenocarcinoma type [29,34,35,135,137,138,140,143,144,147,151,156,157,163,166,167,168,169,174,178,180], SCEC is usually seen with a squamous component [29,34,35,135,138,140,144,151,156,157,163,168,169,174,178,180,183]. Maru *et al.* [153] have observed that mixed oesophageal NECs tend toward a better outcome than pure oesophageal NECs without non-neuroendocrine components, though this was not found when Takubo *et al.* [29] compared the same groups. Non-neuroendocrine components in oesophageal NECs, as well as the expression of the stem cell marker p63 and non-expression of CK20, have led to the general consensus that SCEC originates from an endodermal derived multi-potent stem cell [35,144,151,166,169,178,180].

There have been discussions as to the differences between SCECs and oesophageal squamous cell carcinomas (ESCC) or the lack hereof. However, though clinical features and gross appearances are similar, SCECs are generally more aggressive than ESCCs, [139,158,169,180] with a shorter survival time [162,180] and a higher risk of progression after surgery [180]. Also, in relation to immunohistochemistry, SCECs are often CgA, synaptophysin and NSE positive, whereas ESCCs are not [162]. On a molecular level, Zhimin *et al.* [172] found phosphatase and tensin homolog (PTEN) mutations to be as equally common in SCEC as previously found in ESCC. However, the mutations of epidermal growth factor (EGFR) and V-Ki-ras2 Kirsten rat sarcoma viral oncogene homolog (KRAS) commonly found in ESCC were not found when examining SCECs [172]. In another study, cytokeratin 19 fragment antigen 21-1 (CYFRA21-1) and carcinoembryonic antigen (CEA), both markers for the diagnosis and prognosis of ESCC, were found not to be of prognostic significance in SCEC [95].

The proliferation index of SCEC has been observed to be high [185], with Ki-67 levels up to 57%–100% in some studies [146,150,167], and mitotic levels up to 85/10 HPF in others [144]. In the Nordic NEC study, 67% of oesophageal NECs had a Ki-67 index of >55% [4]. Chow *et al.* [185] have shown that this high proliferation index is closely correlated with telomerase activity. Several studies have found an overexpression of p53 in 50%–100% of their cases, hereby postulating that p53 mutation is an important part of SCEC carcinogenesis [29,42,150,167]. Li *et al.* [34] also found all included SCECs (*n* = 6) to have p16 overexpression, which could suggest involvement of a dysfunctional p16/cyclin D1/Rb pathway in SCEC tumourgenesis.

The most effective treatment for SCEC is not yet established as, similar to other NECs, patient numbers are too small for randomised prospective trials and many treatments are polarized from the management of SCLC [186]. Most clinicians are in agreement that multi-modality treatment is necessary [141,146,151,157,164,178,182], with the highest survival rates having been observed after treatment combinations of chemotherapy and radiotherapy [147,177], chemotherapy and surgery [34,139,141,149,184] or surgery, chemotherapy and radiotherapy [143]. As previously mentioned, brain metastases are considerably rarer than in SCLC and prophylactic cranial irradiation is therefore not recommended as a routine treatment [147,151,155,158].

Chemotherapy tends to be platinum-based, as seen in the chemotherapy treatment of NEC of other sites, and is generally recommended due to SCEC often being disseminated at diagnosis and recurring at distant metastatic sites [149,152,159,175,178]. Nemoto *et al.* [159] have shown that metastases occur earlier in patients not receiving chemotherapy and another study has observed that the high proliferative activity seen in SCEC can be suppressed by chemotherapy [146], as well as significantly increasing survival [182]. The question of cisplatin combined with etoposide or irinotecan is again dependent on the region, with many Asian countries choosing irinotecan. A study from Okuma *et al.* [187] found a response rate of 50% in a cohort of 12 patients with extensive disease after treatment with irinotecan and cisplatin. Interestingly, a study from Yan *et al.* [95] found that patients who had a lower level of serum neuron-specific-enolase (s-NSE) before chemotherapy treatment with cisplatin and etoposide (≤17 ng/mL *versus* >17 ng/mL s-NSE) responded better to chemotherapy than those with high s-NSE.

Radiotherapy often consists of a total of 50–60 Gy over a five week period, but as a sole treatment its effects are disappointing [142] and should therefore be combined with other treatment modalities [141,146,179,184].

The role of surgery is undecided, but due to an operational mortality risk, it is generally agreed that surgery should only be performed when of clear survival benefit [188], for example in localized cases [160], and preferably with adjuvant chemotherapy due to the risk of recurrence with distant metastases [141]. It has been shown in a number of studies that lymph node metastases are a significant prognostic factor in patients receiving surgery as first line treatment [139], and if radical surgery and adjuvant chemotherapy are given to patients without lymph metastases or patients with limited disease, relatively good long-term survival can be achieved [139,146,149,151,163,165,180,188]. A study from Hou *et al.* [143] showed that the combination of surgery and radiotherapy could give a median survival of 23 months in limited stage SCEC, and if chemotherapy was added, survival time increased to 25 months. Another review of 64 cases, with a possible overlap of patients from the Hou *et al.* study [143], found no significant difference in the survival of patients treated with chemotherapy and radiotherapy *versus* surgery and radiotherapy [181]. Similarly, no difference in survival was found between patients treated with chemotherapy and radiotherapy or chemotherapy and surgery in a meta-analysis of 148 articles [189]. However, a review compiling studies of limited stage SCEC from Meng *et al.* [155] argues that chemotherapy and radiotherapy are the best treatment for limited stage SCEC, especially if malignancies are N1 [155].

Cases of long-term survival (>2 years) have been observed after treatment with surgery [144,152,156,161,185], radiotherapy [152,154,183], chemotherapy [175], surgery combined with chemotherapy [29,140,146,149,152,155,160,162,163,165,168,183], surgery combined with radiotherapy [175,183], chemotherapy combined with radiotherapy [29,136,145,146,147,152,158,168,170,171,174,175,177,179] and surgery combined with both chemotherapy and radiotherapy [147,150,154,156,157,159,169,183]. It has been argued that the possibly higher number of long-term survivors after surgery could be due to selection bias, as patients receiving surgery as primary treatment are usually of limited stage and without distant metastases compared to chemotherapy patients who often have disseminated disease [171].

In relation to large cell carcinomas of the oesophagus, too few cases have been reported as yet to be able to draw conclusions regarding best forms of therapy [190]. One Chinese study including five large cell oesophageal NECs showed that the only statistically significant difference between small cell and large cell groups was age, with large cell tumours being found in older patients [144]. This is supported by Maru *et al.* [153], who found no significant differences between small and large cell carcinoma of the oesophagus in relation to survival, suggesting that both morphological types respond similarly to the same treatment, which is consistent with NECs of other GI sites.

Survival times for SCEC are usually dismal, with median or mean survival time rarely surpassing 20 months [4,29,92,95,136,137,139,141,142,143,145,146,147,148,149,150,151,152,153,154,157,158,159,160,161,162,163,165,167,168,171,178,179] and averaging a median of 12 months [4,29,92,95,136,139,142,143,145,146,147,148,149,150,151,152,153,154,158,159,160,162,163,165,168,171,178,179]. Survival has improved slightly from earlier years [135,137,142,143,145,160] but there are still few long-term survivors. Survival rates can be difficult to interpret in some cases due to articles not specifying survival time; *i.e.*, if survival time is from diagnosis or from start of treatment [136,137,146,171]. Prognostic factors affecting survival are generally age [159], limited or extensive disease [147,152,153], TNM classification [168] and local treatment *versus* local combined with systemic treatment [141,182,184]/chemotherapy [152,164,182]. Yan *et al.* [95] also found s-NSE to be predictive of survival, with patients who had a s-NSE ≤17 ng/mL reaching a median survival time of 18 months, in comparison to those with a s-NSE >17 ng/mL reaching a median survival time of only 6 months. Another study found leucine-rich repeating-containing G-protein coupled receptor 5 (Lgr5) overexpression to be correlated to lymph node metastasis, tumour stage and response to chemotherapy, also showing a tendency to poorer survival in comparison to patients with low Lgr5 expression [173].

## 12. Stomach

Gastric NECs (GNECs) are a rare form of GEP-NEC with almost one thousand cases reported in the literature [1,3,4,11,13,28,31,34,38,54,56,61,68,81,82,87,191,192,193,194,195,196,197,198,199], and representing up to 1.5% of gastric/gastric resected cancers [28,192,193,194,196,197]. Neoplasms are often solitary and between 4 and 8 cm in size [61,64,191,192,193,194,195,196,197,198,200], occurring with a higher frequency in 60–70-year-old men [28,34,37,38,56,81,191,192,193,194,195,196,197,198,199,200], and presenting with regional lymph node or distant metastases at diagnosis [28,34,38,61,63,64,81,191,192,193,194,195,196,197,198,200], as well as vascular and lymphatic invasion [63,64,192,193,195,196,197,198]. Main symptoms include epigastric/abdominal pain, gastro-intestinal bleeding, nausea/vomiting, weight loss and dysphagia [70,192,197,200]. Tumours are located most frequently in the lower third of the stomach, with remaining tumours distributed equally between the upper and middle thirds [63,191,192,193,194,195,196,197,198]. Distant metastases are usually found in the liver [38,39,81,192,193,194,196,197,198,200] and, as in SCEC, brain metastases are very rare [195]. Predisposing factors were only mentioned in one study [192], making it difficult to conclude whether smoking or excessive alcohol consumption is associated with GNECs.

GNECs are classified on the basis of the WHO 2010 criteria for GEP-NECs as previously described [20]. However, the WHO 2010 classification also includes a description of gastric NENs based on a separate classification system which was already proposed by Rindi *et al.* in the late 1990s [201,202]. This additional classification system includes four types of gastric NENs and is based on their cell type (ECL or non-ECL), differentiation status (well or poorly differentiated) and association to hyper-gastinemic conditions. Types 1–3 are all well-differentiated tumours arising from ECL cells; type 1 is associated with predominant atrophic gastritis, type 2 is associated with gastrinoma as a part of MEN-1/Zollinger-Ellison syndrome, and type 3 is a sporadic type not associated with any specific gastric pathology [201,202]. Type 4 neoplasms are mentioned specifically in Rindi’s original classification, however, in the WHO 2010 classification they are described solely as GNECs. Type 4 gastric NENs or GNECs are considered to be a separate entity from types 1–3, as non-ECL cell, sporadic, poorly differentiated high-grade neuroendocrine carcinomas. Several studies chose to combine types 3 and 4 as all sporadic, solitary cases of gastric NENs, meaning that GNECs are then classified as a type 3 neoplasm [203]. Another element to the classification and grouping of GNECs is that in numerous articles GNECs and high grade gastric MANECs are grouped together as type 4/type 3 tumours, or as general NECs. This is also seen in yet another classification “The Japanese Classification of Gastric Cancer” where both gastric NECs and MANECs are grouped together as “Endocrine cell carcinoma” (ECC) and all other gastric NENs are grouped as “carcinoid tumour” (CD) [196]. These different classifications can obviously cause confusion, especially when not specified, as GNECs can potentially be grouped together with MANECs and defined as type 3, type 4 or ECC.

The typical modalities used for the diagnosis of GNEC are gastroscopy and endoscopic biopsy [203,204], followed by histological and IHC studies [195,205], as well as thoracic and abdominal CT or FDG-PET for staging [204,206]. ENETS’ TNM classification of GNECs [207] and the WHO 2010 grading system [20] have been validated as good prognostic tools for GNECs [61]. When comparing the ENETS and UICC staging systems it has been has shown that there is a large difference in the pathologically assessed (p-)Stage between the two, probably due to differences in the N-category, but both statistically reflect the prognosis of patients with GNECs and MANECs [196]. Laudry *et al.* [208] have also created a staging system for gastric NENs from the SEER-database.

GNECs can be of small cell or large cell type with a similar survival seen in the two groups [191,193,197]. Both small and large cell GNECs can have a macroscopic appearance similar to gastric adenocarcinomas [205]. However, large cell carcinomas are particularly difficult to differentiate from solid, poorly differentiated adenocarcinomas due to a similar microscopic appearance. Recognition of histologic features suggestive of neuroendocrine differentiation is therefore critical especially as GNECs have been reported to have a significantly worse prognosis than gastric adenocarcinomas [194,209]. GNECs (both small and large cell) are generally more aggressive than gastric adenocarcinomas, with a higher prevalence of lymphatic and venous involvement and postoperative liver metastases [193].

GNECs are frequently associated with non-neuroendocrine components, mainly of adenocarcinoma type [34,61,62,64,192,193,195,197,198]. As previously mentioned, many studies concerning GNECs chose not to differentiate between NECs and MANECs [193,196,199]. A study from Ishida *et al.* [193] has shown that there is no statistical difference in survival between pure GNECs and GNECs with adenocarcinoma components, nor between GNEC and MANEC groups.

The observation that many NECs have adenocarcinoma components suggests a histogenetic relationship between NECs and adenocarcinomas. The hypothesis for GNECs’ origin is as SCECs’: a common multi-potential epithelial stem cell precursor gives rise to GNECs, squamous cell carcinomas, adenocarcinomas, or combinations of the three, explaining the composite nature of GNECs. Nishikura *et al.* [199] found a concordance of p53 mutational status in 73.3% of ECC tumours and their adenocarcinoma components supporting the theory of a shared cell of origin for the two carcinomas.

Several studies have observed that GNECs have nuclear accumulation of p53 [38,42,196,198] which is correlated with Ki-67 expression [196] and could partly explain their carcinogenesis. Loss of E-cadherin has been observed to be correlated with lymph node metastases [191] and VEGF and PD-ECGF are suggested to be inducers of hepatic metastasis [209]. Expression of AMACR has been observed in 90% of GNECs, and was found to correlate with the Ki-67 index [64]. Ki-67 index is not always assessed, but tends to be approximately 70% [64,198].

Optimal therapy for GNECs remains yet to be established. GNECs are often treated with radical surgery [195] and adjuvant platinum-based chemotherapy, but no standard regime exists. A study from Huang *et al.* [192] has shown that surgery with adjuvant chemotherapy for patients with small cell gastric NEC (GSCC) can result in a median overall survival of 48.5 months in comparison to patients only receiving surgery with a median overall survival of 19 months, suggesting that GNECs are chemo-sensitive tumours. In contrast, Kubota *et al.* [196] found no beneficial effect of adjuvant chemotherapy; however, their chemotherapy regime differed from Huang *et al.*’s [192]. There are few studies showing results of non-surgical treatment. One study has suggested the possibility of cisplatin and irinotecan as primary treatment, with response rates of 75% and PFS of 212 days with the chemotherapy regime, compared to PFS of 177 days after surgery. As in NECs of other GEP-sites, there is a need for larger prospective studies concerning treatment, or better reporting of the current treatments given. Irrelevant of treatment, overall survival time can vary from a median of 8–33 months [4,34,63,92,192,210] or a mean of 14.9–40.1 months [81,197], with a five-year survival rate of 30%–60% [193,194,195,196,198,200]. Factors affecting survival are tumour recurrence [193,195] and high Ki-67 (>60%) [191].

## 13. Pancreas

NECs represent ≤1% of pancreatic neoplasms [20,211,212] and approximately 15% (3.9%–55%) of pancreatic NENs [67,213,214,215,216,217,218,219,220,221,222,223,224,225,226,227,228,229,230,231,232,233,234,235], with over 2000 cases described in the literature [1,3,4,13,88,90,211,225,226,228,230,231,233,234,235,236,237]. Generally, more men are diagnosed [12,85,128,211,222,231,238], but differences are not as large as seen in oesophageal or GNECs. Age ranges from 13–90 years, with the largest number of patients being diagnosed between 50 and 70 years of age [85,128,211,222,238,239]. Only one study with a relatively small cohort has mentioned predisposing factors, finding 50% of their patients to be smokers [212]. Symptoms are also rarely described, but when mentioned encompass abdominal pain, jaundice, weight loss, cachexia, ascites and disorientation [211,212]. Tumours are usually 4 cm in size, varying from 2–18 cm [67,102,128,211,217,222,239], and are typically located in the pancreatic head [128,211,222]. The majority of patients have regional lymph node metastases [67,128,211,222,236,238,240] and distant metastases [12,67,88,128,211,222,228,236,238,239] at diagnosis, as well as vascular and lymphatic invasion [67,128]. Distant metastases are most often found in the liver [128,211,228,238], and rarely in the brain [211]. PNECs are generally non-functioning [128,211,225,236,239] but are occasionally seen as insulinomas, glucagonomas, gastrinomas or VIPomas [128,225,238,241]. Non-functioning tumours are without clinical symptoms of hormonal hyper-secretion, however, they can show IHC positivity for hormones. In this case, the hormone may be produced but not secreted, is secreted but in so low levels as not to give a hormonal syndrome, or the hormone itself is clinically inert and does not give rise to a syndrome [242].

Endoscopic ultrasound (EUS)-guided fine-needle aspiration (FNA) is a good modality to identify and diagnose PNECs, though an exact Ki-67 index may be difficult to obtain. A study from Figueiredo *et al.* [221] assessed the sensitivity of EUS-FNA in diagnosing pancreatic NENs and found that after three attempts 90% of pancreatic NENs were diagnosed (78% after the first, 87% after the second, and 90% after the third attempt). Recently, studies have also shown that there is the possibility of taking core biopsies via EUS, thereby further increasing diagnosis accuracy [113]. Ultrasound (US), dual-phase CT and MRI are recommended for localizing primary and metastatic tumours [242]. The sensitivity and specificity of identifying PDECs on contrast-enhanced CT have been found to be 86% and 63%, respectively [102]. Specifically, small cell pancreatic carcinomas are homogeneous, well-defined, hypo-attenuated with minimal enhancement after intravenous contrast injection on CT imaging, and can be hypo- or hyper-echoic on ultrasound [212]. A study from Rodallec *et al.* [102] also found PDECs to be hypo-attenuating on enhanced CT imaging, and calcification to be associated with WD-NECs. Angiogenesis has been shown to play a role in the progression of different neoplasms. A study from Couvelard *et al.* [243] showed that VEGF-expression and density of microvessels are correlated negatively with WHO classification category, meaning that PNECs have the lowest VEFG-expression and microvessel density in comparison to well-differentiated endocrine tumours. This finding appears to be tumour-specific and in contrast to findings in other epithelial cancers, explaining the hypo-attenuation of PNECs on CT-imaging. Somatostatin receptor imaging, as well as FDG-PET might also be used for diagnostic purposes, as previously described in the general GEP-NEC section. The Nordic NEC Study [4] found 46% of included PNECs to have a high uptake on SRS, however, many included PNECs had a lower range Ki-67 index and tumours with a higher Ki-67 index were less likely to be scanned.

There are three different TNM-based staging systems for pancreatic NENs in use within the literature. The ENETS’ staging system [207] (specific for pancreatic NENs), the UICC (International Union for Cancer Control) staging system [244] (applied from pancreatic adenocarcinomas) and a staging system proposed by Martin *et al.* [230] based on the SEER database. The ENETS TNM staging system has been validated by several studies [219,222,225,232,239], though Scarpa *et al.* [245] suggested modifications. The UICC’s staging system is endorsed by both the AJCC and the WHO 2010 classification of NENs and has been validated by Strosberg *et al.* [233]. The major differences between the ENETS and AJCC-TNM pancreatic staging systems are confined to the tumour definition and derived stages, but also most importantly, the UICC/AJCC/WHO staging system is specific for NETs and not meant for PNECs; high grade carcinomas are required to be excluded for analysis of the UICC TNM-staging system [244,246]. It is essential that studies specify the TNM-stage used, as studies have shown that the incongruities of the two systems are relatively frequent [226]. In a head to-head comparison of the two staging systems, the ENETS TNM-staging system was found to be more accurate in predicting prognosis [245,246]. The third staging system based on the SEER-database has not been widely used [230].

PNENs are classified by the WHO 2010 classification criteria [20]. The criteria for PNECs have changed during the last decade, causing confusion amongst clinicians and when reviewing articles from different time periods. In the WHO 2000 and 2004 classifications, PNECs were described as poorly differentiated endocrine carcinomas (PDECs) composed of small/intermediate-size cells with a proliferation rate of >10 mitoses/10 HPF [24] and a Ki-67 index >10% [24]–15% [22]. In the WHO 2010 version, PNECs are defined by the presence of >20 mitoses/10 HPF or a Ki-67 index >20%, and are divided into small cell or large cell NECs [20]. The exclusion of large cell carcinomas in the 2004 WHO classification has undoubtedly caused numerous large cell PNECs to be classified as well-differentiated endocrine carcinomas, and lead to confusion in relation to correct diagnosis and grouping of pancreatic NENs. This explains why several studies have a higher number of G3 (high grade) tumours than PDECs, due to tumours with intermediate or large cells being classified as well-differentiated endocrine carcinomas according to classifications previous to 2010 [247]. Another critical issue is the difference of 10 mitoses/10 HPF in the proliferation rate between the 2004 and 2010 versions, meaning that many tumours classified as PNEC/G3 tumours could have been G2 tumours. Yet another grading system has also been in use, the “Hochwald grading system”, which meant to stratify well-differentiated pancreatic NETs into low grade and intermediate groups by level of necrosis and mitotic count [248]. Despite a strong predicating power [248], this grading system does not include NEC, and is therefore not relevant for PNECs.

The WHO 2010 grading system has been proved to be predictive of survival [246], and to be justified over the WHO 2004 grading system [248].

Small cell and large cell NECs are genetically related entities [237]. Due to the earlier WHO classification excluding large cell NECs, it is difficult to say if small or large cell carcinomas are the most common. In studies where both small and large cell carcinomas are included, large cell types are often greater in number [85,93,128,219]. A large multi-institutional study found the median survival rate to be 16 months for large cell PNECs and only 6 months for small cell PNECs. However, this was not statistically significant [128]. Another study from Korse *et al.* [3] also found a difference in the survival rates of small cell and large cell PNECs, with the five-year relative survival rate in comparison to the general population being 39% for large cell PNECs and only 6% for small cell PNECs. Evidence is insufficient to evaluate whether treatment modalities are equally efficient between the two groups.

When assessed, PNECs have a Ki-67 index between 22% and 95% [128,238], with the value being similar in both small and large cell PNECs. The Nordic NEC study [4], which included NECs of different GEP-origins, showed that patients with a Ki-67 index over 55% had a poorer prognosis. This difference in outcome dependent on the Ki-67 index was not replicated in a study by Basturk *et al.* [128] where a Ki-67-index higher or lower than 55% made no difference in survival. A major difference between these two studies is that Basturk *et al.* [128] had excluded all neoplasms that were not poorly differentiated despite a Ki-67 index >20%. Sixty percent of NECs in the Basturk *et al.* study [128] had a Ki-67 index >60%, similar to a Japanese study with 61% of PNECs having a Ki-67 >55% [92], whereas in the Nordic NEC study [4] non-poorly differentiated tumours were not excluded, and only 30% of cases had a Ki-67-index >55%. It could be assumed that a number of tumours in the Nordic NEC Study [4] were well-differentiated with a Ki-67 index >20%, which might explain the cut of point of 55% (Ki-67); meaning that well-differentiated tumours would be in the lower Ki-67 range and increase survival levels, whereas the poorly differentiated NECs would be in the higher range and lower survival levels.

The histological diagnosis of PNEC can be difficult to distinguish from acinar cell carcinoma or mixed acinar cell carcinoma (acinar-neuroendocrine carcinomas). Both have high proliferation rates and can have similar histological features. In the study from Basturk *et al.* [128], 17 cases originally diagnosed as PDECs proved to be acinar cell carcinomas or mixed acinar-neuroendocrine carcinomas. This implies that acinar differentiation should be confirmed by ICH stains for trypsin and chymotrypsin. In younger patients, it could also be relevant to exclude the possible diagnosis of primitive neuroectodermal tumour by IHC staining for CD99, which is negative in pancreatic PDEC and positive in primitive neuroectodermal tumours [128]. Due to small cell carcinoma of the pancreas being identical to SCLC, it is again necessary to exclude a pulmonary primary, and also to be aware of other possible extra-pancreatic NECs. Only one study has specified the number of PNECs with non-neuroendocrine components, finding approximately 16% of tumours to be of combined type, most frequently with ductal adenocarcinoma [128]. There was no statistically significant difference in survival between the pure or combined NECs [128].

The origin of pancreatic NEC is similar again to that of SCEC and GNEC. There have been suggestions of well-differentiated tumours evolving into PDEC, but this is generally considered to be a rarity, and the main origin of pancreatic NECs is thought to be different to that of well-differentiated NETS, supported by the many findings of different genetic mutations between the two groups suggesting that they are separate entities [128,237]. Co-inactivation of p53 and Rb/p16 pathways seem to be a fundamental genetic feature in PNECs [237].

Another theory is that PNECs arise from pre-existing ductal lesions, however, few genetic associations between ductal adenocarcinomas and PNECs suggest that this is not the case [237]. For example, genetic changes common in ductal adenocarcinomas such as mutations in KRAS and loss of SMAD4/DPC4 were found to be infrequent in PNECs [237]. Despite these genetic differences, PNECs are clinically similar to ductal adenocarcinomas [211,212] and studies have also shown similar survival rates [212,222,236], as well as prognosis after surgery for the two cancer forms [236].

Other molecular studies have reported of gains in chromosome 3p [249] and L1 expression [224] in PNECs. One study reported that 50% of pancreatic PDECs have a gains in chromosome 3p, this was however not associated with survival [249]. Interestingly, pancreatic NETs (PNETs) had most commonly losses and not gains in the same chromosome [249]. In a study from Kaifi *et al.* [224], PNECs were found to be significantly correlated with L1 expression (a cell adhesion molecule that plays a role in the development of the nervous system) in comparison to PNETs. However, there were only nine PNECs included in the study.

There is no consensus on the treatment of PNECs at the present time. The importance of surgical treatment is controversial. ENETS’ consensus guidelines from 2012 recommend an aggressive surgical approach for selected patients, but liver resections or transplantations are not recommended [242]. A review by Ito *et al.* [250] recommend surgery should be considered in patients with limited disease. However, Cherenfant *et al.* [217] suggest that pancreatic resection should be strongly considered if the patient is fit for operation, regardless of tumour size. Due to PNECs often being metastatic and large at diagnosis, the possibilities of resection are smaller in comparison to PNETs. For example, in a study from Bettini *et al.* [251], only 23.5% of PDECs were resectable at diagnosis (82% of the whole cohort of pancreatic NENs received surgery), and only 25% of grade III-IV tumours in the SEER data-base were resected in comparison to 79% of grades I and II [252]. In another study from Bettini [253], the author suggests that surgery should be limited to well-differentiated carcinomas with a Ki-67 index <10%. Apart from the lower chance of a possible resection, there is also the question of if a R0 or R1/R2 resection actually gives any survival benefit. A study from Fischer *et al.* [222] showed no difference in prognosis between R0 and R1/R2 resections in PNECs however, this was judged on only four R0 cases. In a study from You *et al.* [235], all R0 cases recurred within 7 months and their two-year survival was only 19%. A study from Sellner *et al.* [236] showed that 45% of pancreatic PNECs were subjected to R1/R2 resection when operated on, and that R1/R2 resections give no better survival rate than palliative procedures, with the outcome after surgical treatment being as poor as for ductal pancreatic carcinoma.

Chemotherapy for non-resectable patients or as adjuvant therapy for resected patients often consists of a etoposide and cisplatin regime [254] or, as in many Asian countries, an irinotecan and cisplatin regime [92]. Iwasa *et al.* [255] found a 14% overall response rate for etoposide and cisplatin treatment of pancreatic and hepatobiliary NECs. Similar response rates have been reported by Yamaguchi *et al.* [92], with 12% (7/34) of hepato-biliary-pancreatic NEC patients responding to the same regime. However, higher response rates were observed in patients treated with irinotecan and etoposide, with 39% (7/18) of patients responding to treatment [92]. Second-line treatments such as FOLFIRI and temozolomide have been tested in larger mixed cohorts including PNECs [85,125]. Olsen *et al.* [125] suggested that temozolomide might have a better effect in PNECs than in NECs of other locations, due to a median survival rate of 7 months for PNECs and only 2.9 months for other NECs. Few have assessed the possibilities of radiotherapy specifically for PNECs. Patients with PNEC should be strictly followed-up every 6 months with biochemical markers and CT/MRI scans according to the ENETS consensus guidelines [242].

As with other GEP-NECs, survival for PNECs is dire, with median survival rates ranging from 5–21 months [4,92,128,228,245,252,256] and five-year survival rates ranging from 0%–28.3% [128,215,221,223,228,229,236,245,256]. Few studies include non-resectable patients, implying a selection bias in relation to survival. When evaluating only non-resectable patients, survival drops to approximately 6 months [236,255]. In comparison to other organs, there are conflicting findings as to whether PNECs have better or worse survival. The Nordic NEC study [4] reported a higher survival rate for PNECs in comparison to the colon (median survival of 15 *versus* 8 months). However, Lepage *et al.* [1] found a 1.6 excess hazard ratio of death for small cell PNECs in comparison to small cell NECs of the large bowel. Yamaguchi *et al.* [92] also found a worse survival for PNECs, with a median survival time of 8.5 months in comparison to 13.4 or 13.3 months for NECs of the oesophagus or stomach. In the same study, the primary NEC site was prognostic of survival, with NECs of the hepato-biliary-pancreas system having a worse survival than NECs of the gastrointestinal tract.

Long-term survivors have been reported [128,212,221,222,236], with the longest survival reaching 173 months [212]. Age, sex, tumour location, lymphovascular and perineural invasion, margin status, T stage, lymph node metastases are all significantly associated with survival [128].

A major problem when reviewing the characteristics of PNECs is that in many cohorts PNECs are not separated from PNET. Another critical issue is the lack of information concerning duplicate cohorts; many articles have patient cohorts with overlapping time-periods at the same hospitals with no documentation of cases having been counted previously. Also, many cohorts select surgical patients only, meaning that interpretation of survival could be subjected to selection bias due to patients with localized disease and higher performance score undergoing surgery in comparison to patients deemed unresectable. Another central problem is that many larger databases have few histological diagnoses, resulting in less awareness of PDECs and their specific management, for example in the National Cancer Data Base (NCDB) study from the U.S., only 12.3% of patients had been given a histological differentiation diagnosis (well-differentiated *versus* PDEC) [241].

## 14. Gallbladder

Neuroendocrine carcinomas of the gallbladder are rare, with less than 200 cases reported in the literature [7,15,79,257,258,259,260,261], constituting 0.4%–4% of malignancies in the gallbladder and extra-hepatic bile duct [257,258,260,261]. In contrast to NECs of other GEP-sites, NECs of the gallbladder are most common in females [257,258,259,260,261]. Tumours present at an age of 60–70-years [257,258,259,260,261] with signs and symptoms often including abdominal pain and occasionally jaundice, weight loss, palpable mass, fever, acholia, pruritus and ascites [260,261,262]. NECs of the gallbladder are usually non-functional, but there have been rare reports of patients with Cushings syndrome and paraneoplastic sensory neuropathy [20]. The majority of patients tend to have cholelithiasis [257,259,260,261], a finding not dissimilar to patients with adenocarcinomas of the gallbladder. Tumours are generally large (>2 cm) [257,258,259] and are most often found in the gallbladder itself, though tumours also occur in the extrahepatic bile ducts [258]. Regional lymph node metastases as well as distant metastases are common at presentation [15,258,259,260,261,263].

Few gallbladder NECs are diagnosed pre-operatively as symptoms are vague and imaging techniques cannot differentiate between gallbladder adenocarcinomas and gallbladder NECs. Imagery such as EUS, MRCP or ERCP are useful for diagnosing the primary tumour, whereas CT is necessary to define the extent of loco-regional disease, for which FDG-PET can also be of use [263].

Due to the rarity of NENs of the gallbladder there is currently no specific TNM-staging system for these neoplasms. The WHO 2010 classification system recommends use of the TNM-staging system for adenocarcinomas of the gallbladder [20].

NECs of the gallbladder can be of small or large cell type, of which the large cell type is very rare [263]. Neuroendocrine cells do not exist in the normal gallbladder epithelium and occur only in intestinal or gastric metaplastic gallbladder mucosa, seen secondary to cholelithiasis or chronic cholecystitis [263]. According to a study from the SEER-registry, there is a higher ratio of small cell carcinomas to NETs in the gallbladder than any other GI site [258]. However, the SEER-registry has a low percentage of histological diagnoses and this could affect the number of NEC cases, as more aggressive cancer might be histologically examined more often.

Small cell carcinomas of the gallbladder can resemble malignant lymphoma, rhabdomyosarcoma or undifferentiated carcinomas [259]. These differential diagnoses can be excluded by morphological characteristics or immunohistochemical staining. Many tumours have adenocarcinoma components which often stain positive for CEA, whereas CEA reactivity is limited in small cell carcinomas of the gallbladder. Histogenesis of small cell carcinoma of the gallbladder is hypothesized to be of endodermal origin, with possible differentiation along epithelial or endocrine lines [257,260]. Small cell carcinomas of the gallbladder often have positive p53 immunoreactivity [259].

As previously mentioned, gallbladder NECs are similar to poorly differentiated adenocarcinomas of the gallbladder in relation to symptoms [257,259,260,261], disease spread, prognosis and survival [260,261,263]. This is also reflected on a molecular level, as gallbladder NECs seem to have the same genetic abnormalities at similar frequencies to adenocarcinomas of the gallbladder. For example, Maitra *et al.* [259] found that 44% of gallbladder NECs had abnormalities in p16 (a cell cycle inhibitor), corresponding to a reported 50% of adenocarcinomas. From the same study, abnormalities were also found at the locus for the tumour suppressor genes DCC and DPCA, again at a similar frequency for both NECs and adenocarcinomas (43% *versus* 31%) [259]. In contrast to these similar molecular abnormalities, Nishihara *et al.* [261] found that the rate of DNA aneuploidy was significantly higher in small cell gallbladder NECs than in adenocarcinomas of the gallbladder.

The treatment of gallbladder NECs is controversial. Generally surgery is performed and if tumours are unresectable medical treatment is given. The role of chemotherapy and radiotherapy is unclear, however, multimodal treatment is thought to prolong the survival of small cell gallbladder carcinomas [263]. Chemotherapy seems to benefit patients in combination with surgery which is reflected in survival rates [260]. A study from Albores-Saavedra found that patients who underwent surgery without chemotherapy lived a maximum of 4 months, whereas patients with combined surgery and chemotherapy survived 11–13 months; this was however based on small patient numbers [257]. Also, a study from Maitra *et al.* [259] showed a median survival rate of 31 months with all patients having undergone surgery and chemotherapy.

Mean or median survival times differ from 4–31 months [15,259,260] and one year or five year survival rates have been reported at 8% [258] and 28% [1], respectively. Only six long-term survivors [259,260,261] have been reported with one still alive after almost 16 years [260]. Survival is significantly worse for small cell carcinoma of the gallbladder than that of papillary adenocarcinoma and well-differentiated adenocarcinoma in pTNM-stage II-IV [261].

## 15. Small Intestine and Ampulla of Vater

NECs are exceedingly rare in the small intestine. Most are found in the ampullary region, hereafter the duodenum and proximal jejunum [20,264]. To date, no NECs have been reported from the distal jejunum and ileum [20]. A total of 205 small intestinal NECs have been reported in the literature [1,3,73,92,264,265,266], those located in the Ampulla of Vater representing between 0.25%–3% of all ampullary tumours [264,267]. Information regarding the tumours’ size and locations within the small intestine, as well as patient details and treatment are often not specified within these cohorts [1,3,73,92,266].

The few non-ampullary NECs reported in the literature have been found in 50–60-year-old men [20]. In the cases of ampullary NECs, patients often present at 70 years of age and are more likely to be male than female [264]. Tumours are usually 0.8–4 cm in size and most patients have liver or other distant metastases at diagnosis [264,265]. In the study from Nassar *et al.* [264], two of the three patients with disseminated disease had brain metastases, which is an unusually high percentage in comparison to NECs of other GEP sites. Tumours can be of small or large cell type [3,20,267] and many contain non-neuroendocrine components, suggesting a common origin for NECs and adenocarcinomas, though their pathogenesis may differ [264]. The Netherlands’ Cancer Registry found the incidence of large cell NECs to be higher than small cell NECs in the small bowel (0.04 *versus* 0.00 per 100,000 persons/year) [3].

NECs of the ampulla of Vater are considered to be similar to poorly differentiated adenocarcinomas of the same area in relation to clinical symptoms and gross appearance. However, there are a number of differences in the molecular abnormalities of these two tumour types. Nassar *et al.* [264] found that almost all NECs of the ampulla of Vater had normal p27 expression, whereas all poorly differentiated adenocarcinomas showed loss of expression of p27. Also, retinoblastoma (Rb) protein expression was lost in 6/10 NECs but no loss of expression was seen in the poorly differentiated adenocarcinomas [264]. These differences support the theory of different molecular pathogenesis in the development of NECs and adenocarcinomas in the ampulla of Vater.

NENs of the small intestine and ampulla of Vater have their own TNM-staging systems proposed by ENETS. There are two separate staging systems: one for tumours of the duodenum, ampulla and proximal jejunum [207] and another for tumours of the distal jejunum and ileum [268]. A third TNM-staging system for all tumours of the small intestine has also been published and is based on the SEER-registry database [269].

Survival data for NECs of the ampullary region differs from 4–52 months [264,265] with a mean of 14.5 months [264]. According to Yamaguchi *et al.* [92], small intestinal NECs were found to have a median survival time of 29.7 months, or according to Lepage *et al.* [1], a relative one-year survival rate of 27.1%. The Netherlands’ Cancer Registry found a five-year relative survival rate of respectively 27% and 3% for large cell and small cell NECs [3], similar to the five-year survival rate from the SEER registry of 15.7% for small and large cell NECs combined [267]. Patients are usually treated with surgery and occasionally with adjuvant chemotherapy or radiotherapy [264]. The few cases reported make it difficult to draw any conclusions on the most effective treatment, and there is currently no standardized protocol for treatment.

## 16. Appendix

Neuroendocrine neoplasms of the appendix are quite common, however, NECs of this region are exceedingly rare. The SEER-registry database has reported 22 cases of poorly or undifferentiated NENs of the appendix [270]. Apart from this, only two other cases have been reported [20] with one of the patients dying after 2 months and the second still alive after 65 months [20]. ENETS, AJCC and Landry *et al.* have all proposed a specific staging system for NENs of the appendix [21,268,270]. These systems differ in several ways. The AJCC staging system excludes NECs, also the T-stages of ENETS and AJCC differ considerably [21].

The extreme rarity of appendiceal NECs make conclusions on this group of neoplasms impossible at the present time.

## 17. Colon and Rectum

Colorectal neuroendocrine carcinomas (CRNECs) constitute less than 1%–2% of colorectal cancers [5,271,272,273] with approximately 2000 cases reported in the literature [1,3,4,5,12,25,34,36,38,46,47,66,67,68,82,84,92,272,274,275,276,277,278,279,280,281,282,283]. The majority of patients are diagnosed in their sixth or seventh decades [5,25,34,38,46,274,278,279,280,281,283], with an equal number of cases between men and women (52% *versus* 48% of cases) [5,12,25,34,38,46,272,274,275,276,278,279,280,281,283]. Symptoms and signs of CRNECs can encompass altered bowel habits, occult GI-bleeding, hematochezia, weight loss, pain, increased abdominal girth, malaise as well as systemic non-specific symptoms [271,278,284]. Tumours are often located in the right colon or rectum, accounting for approximately 42% and 37% of CRNECs, respectively [48,271,272,276,278,279,281,283]. Tumour size can vary considerably from several millimetres up to 15 cm [48,58,67,278,281,284] for both rectal and colonic tumours, though median or mean values are usually around 4 cm [58,67,281]. The majority of CRNECs display vascular, lymphatic and perineural invasion [278,279,281,282,285], as well as lymph node and distant metastases at diagnosis [5,12,34,38,48,67,109,119,274,278,279,280,281,283].

NECs of the colon or rectum are commonly diagnosed via biopsies taken during endoscopy [286,287]. TNM-stage is often assessed by multi-slice CT for imagery of the thorax, abdomen and pelvis, however, MRI is superior for determining liver metastases [286,287]. MRI of the pelvis is mandatory prior to rectal surgery and is the image modality of choice for T2, T3, T4 and nodal-positive tumours [286]. When evaluating rectal tumours, endoanal/rectal ultrasound has been found to be effective in assessing accurate tumour size, depth of invasion and possible para-rectal lymph node metastases [286,287]. FDG-PET can be useful for staging [287].

There are currently three different TNM-based staging systems for CRNECs; the ENETS’ staging system for all colorectal NENs [207], two SEER-based TNM-staging systems for the colon and rectum, respectively, [288,289] and the AJCC staging system for NETs arising in any part of the gastrointestinal tract [277]. An essential difference between the ENETS and AJCC TNM-staging systems is that the AJCC staging system excludes NECs. The SEER and ENETS systems differ in comparison to their tumour populations, T definitions and staging levels [207,288,289]. All mentioned staging systems have been validated by Chagpar *et al.* [277], however, the SEER-based staging systems were found to be more difficult to apply than the ENETS’ and AJCC’s systems.

Colorectal NECs should be histologically distinguished from lymphoma, squamous cell carcinoma with basaloid features, malignant melanoma and adenocarcinomas by immunohistochemistry (and morphological characteristics) [278], especially as CRNECs seem to have a poorer prognosis than colorectal cancer (CRC) [278,282]. CRNECs can be of either small cell or large cell type [25,53,93,274,281]. Proliferation rates are generally high, with Ki-67 values up to 90% and mitotic cell counts up to 140/10 HPF [53,275,278,279]. The Nordic NEC study [4] found 70% of all colonic NECs and 80% of all rectal NECs to have a Ki-67 index of >55%, in comparison to only 30% of pancreatic NECs having such a high an index. Many CRNECs have a non-neuroendocrine component which can occasionally consist of squamous carcinoma, but in the majority of cases is an adjacent adenoma or adenocarcinoma [7,274,278,279,283]. Several studies have shown that there is no difference in survival between small or large cell CRNECs, corresponding to findings from other GEP-NECs [273,281]. However, Korse *et al.* [3] found the expected relative survival of CRNECs in comparison to the general population to be 22% for large cell CRNECs (95% CI 15%–30%) and only 9% (95% CI 4%–18%) for small cell NECs, though there is an overlap of confidence intervals. Another study from La Rosa *et al.* [279] also found a difference in survival between small/intermediate cell and large cell NECs. This discrepancy could be due to La Rosa *et al.* grouping small and intermediate cell groups together. The same study from La Rosa *et al.* [279] found no difference in survival between CRNECs and colorectal MANECs.

The origin of CRNECs is similar to the hypothesis of origin in previously reviewed organs: a pluri-potent epithelial stem cell capable of multidirectional differentiation develops into NECs, adenocarcinomas or combinations of the two [283,285]. Alternatively, a precursor epithelial lesion such as an adenoma/adenocarcinoma differentiates into a NEC [274,283]. NETs are not considered to share the same pathway [283]. It is still unknown as to when the neuroendocrine or adenocarcinoma differentiation occurs in these theories for CRNEC origin, however, Vortmeyer *et al.* [283] suggest that this is a relatively early process.

Several genes and gene mechanisms have been investigated to clarify their possible role in the carcinogenesis of CRNECs. Common known mutations, also seen in other organs, are the p53 mutational overexpression [38,42,279,283], as well as methylation of p16 [38,42] which is also clearly associated with survival [275]. Other mutations and mechanisms more specific to CRNECs include the CpG island methylator phenotype (CIMP), microsatellite instability (MSI) and c-kit mutations. CIMP is thought to be an important mechanism of gene inactivation and has been shown to silence a number of important genes (p16, p14, *etc.*) in colorectal cancer (CRC) as well as many other cancer types. MSI is responsible for 10%–20% of CRCs and is associated to CIMP. C-kit is a proto-oncogene observed in a number of human malignancies such as gastrointestinal stromal tumours and SCLC amongst others.

In a study by Arnold *et al.* [275], CIMP was found much more frequently in CRNECs than in colorectal NETs, and correlated with a high Ki-67 index. A study from La Rosa *et al.* [279] found that microsatellite instability was associated with CIMP confirming a close association between the two. MSI was found in 15% of CRNECs and MANECs, with tumours possessing both CIMP and MSI representing a distinct entity with better prognosis [279]. Conversely, Stelow *et al.* [290] did not find MSI to be involved in the development of CRNECs. Additionally, a number of CRNECs have been found to overexpress c-kit protein, meaning that mutations in c-kit could occur in subsets of CRNECs as a molecular abnormality accumulated during tumour progression [274].

In relation to colorectal carcinomas, Furlan *et al.* [291] found that there were high levels of gene methylation in CRNECs, as well as in sporadic colorectal adenocarcinomas, with both having similar frequencies. However, the DNA methylation profiles were different for the two groups, emphasizing that epigenetic mechanisms target different genes in CRNECs and colorectal adenocarcinomas. Specifically, the methylation of RASSF1, CASP8 and APC was common in CRNECs, whereas the methylation of p16 and PAX6 was common in colorectal adenocarcinomas. Several studies support these findings of similar gene methylation frequencies but different specific genes between CRNECs and exocrine CRCs [275,279]. However, La Rosa *et al.* [279] found that GATA5, known to be hypermethylated in exocrine CRC, was also hypermethylated in 90% of CRNECs.

Clinically CRNECs differ from colorectal adenocarcinomas by being more aggressive [278], with a higher rate of distant metastases at diagnosis [5] or time of surgery [276] and poorer survival [5,273,278,282]. A SEER-database study also found CRNECs to be diagnosed in younger patients compared to patients diagnosed with colorectal adenocarcinoma [5]. Immunohistochemically, CRNECs tend to be CK20 negative, whereas colorectal adenocarcinomas are often positive [36,279].

Treatment of CRNECs is not standardized as yet. ENETS consensus guidelines state that in relation to surgical treatment, rectal and colonic NECs should be treated according to the guidelines for adenocarcinoma. If the cancer is disseminated and not obstructive, treatment with medical, regional, or ablative therapy should be considered [286]. This is supported by Mandair *et al.* [287], who also add that chemotherapy should be considered as adjuvant therapy to surgery, or as first-line treatment in unresectable patients. In a retrospective study by Smith *et al.* [281], resection on the primary tumour gave no statistically significant difference in survival. However, chemotherapy was associated with better survival in metastatic patients. Platinum regimens have proven to be effective in CRNECs [286] but a study from Patta *et al.* [280] showed that despite a positive response, the effect was short lived with a PFS of only 4.5 months and a median overall survival of 9.5 months in patients. These results could be affected by a selection-bias as patients with disseminated disease and poorer performance score are often selected to chemotherapy rather than surgery. Bernick *et al.* [273] recommend cisplatin and etoposide for stage III and IV tumours based on objective responses in their patients.

According to the ENETS guidelines, follow-up for CRNECs <2 cm is annually and for tumours ≥2 cm every 4–6 months the first year thereafter at least annually [286]. Methods of follow up include EUS, colonoscopy, CT, MRI, and for liver metastases MRI with hepatospecific contrast medium or multi-slice CT with multi-phasic liver scanning [286].

Survival for colorectal NECs seems to be even more dire than for other GEP-NECs, with almost all patients dying within a year and median overall survival time often between 4.5–10 months [4,34,53,92,273,274,275,276,278,280,282]. Vascular invasion and expression of CD117 have been found to be independent prognostic factors [279] of survival.

## 18. Conclusions and Future Directions

GEP-NECs are a group of highly malignant neoplasms. Studies have shown that the incidence of this cancer has increased during the past decades [1,2,3,4], though survival has not [1,3]. In this review, we have presented a comprehensive summary of the available literature concerning GEP-NECs. Here, we recapitulate the key points.

### 18.1. The Challenge of Terminology

During the past 34 years, the histological WHO Classification for GEP-NENs has changed four times. This has obviously created confusion and still causes difficulties when comparing studies. Major changes include shifting the lowest Ki-67 index level for NECs (from >15% [22] or >10% for pancreatic NECs [24] to >20% in 2010 [20]) and the recent inclusion of large cell NECs [20]. Certain organs, such as the stomach or pancreas, also have other classification systems based on different criteria, which again, can cause discrepancy in the comparison of studies. Though this discrepancy is problematic, it is the increase of knowledge within the subject of NECs and NENs as a whole that has necessitated these changes and created a need for updates within the classification field.

### 18.2. Different Entities

There is currently a debate as to whether there are different entities within the GEP-NEC group.

First, there is the histological question; are all high grade tumours (G3) poorly differentiated or is there a sub-group of NECs that are well-differentiated but considered NECs due to their high proliferation rates (>20% Ki-67 index)? Also, are there yet more cut-off points in relation to the Ki-index, for example a sub-group above and below 50%? If there are subgroups of well-differentiated NECs and lower Ki-67 index NECs, it is not known whether it is the level of differentiation or level of the Ki-67 index that most affects survival. Studies have described differences in survival time between poorly and well-differentiated NECs [90], whereas others have described differences between NECs with a Ki-67 index above and below 50%–60% [4,109,125]. Currently, there are no criteria defining well *versus* poorly differentiated NENs. Further studies are needed to establish if it is the grade, level of differentiation or a combination of both, that possibly separates groups within GEP-NECs.

Second, there is the question as to whether GEP-NECs are different entities depending on their organ site. This is based on the differences in survival times and treatment strategies between organs. Also, differences in the distribution of small *versus* large cell carcinomas and their different non-neuroendocrine components throughout the GEP-system raise the question of different entities.

### 18.3. Incidence

A number of studies have indicated an increasing incidence of GEP-NECs [1,2,3,4]. A part of the increase could be due to better awareness of GEP-NECs and hereby better immunohistochemistry standards (*i.e.*, increased testing for NENs when faced with a poorly differentiated tumour), implying that a number of previously diagnosed poorly differentiated carcinomas or metastases from an unseen primary are now being correctly diagnosed as GEP-NECs. Changes in classification systems, for example, the inclusion of large cell NECs, can also have affected incidence rates [3,20].

Another important point is that in large database studies few NENs have previously received a specific histological diagnosis. The increased awareness of GEP-NECs could have caused more researchers to be specific in the diagnoses of GEP-NENs, which might have caused an increased incidence.

Lastly, we cannot exclude the possibility of an actual increase of GEP-NECs.

### 18.4. Treatment and Survival

Despite the increase of research concerning GEP-NECs, the survival of these neoplasms has not improved [1,3]. Reasons for this involve the absence of established treatment strategies. Currently, there is no established second-line chemotherapy treatment and many still debate as to whether surgery is of benefit for limited stage patients. Adding to this, the studies available concerning the treatment of GEP-NECs have mixed cohorts (*i.e.*, NENs or NECs from other anatomical sites), making extrapolation of results difficult. Specific, prospective studies for GEP-NECs are needed for clearer guidelines in relation to treatment, which could hopefully improve survival for this aggressive cancer form.

## Supplementary Files

Supplementary File 1
