# Supplementary material for: Neuroendocrine Carcinomas of the Gastroenteropancreatic System: A Comprehensive Review
Source: Diagnostics (Basel). 2015 Apr 8;5(2):119–76. doi: 10.3390/diagnostics5020119 (PMC4665594; doi:10.3390/diagnostics5020119)
Supplement: Supplementary File 1 [file diagnostics-05-00119-s001.pdf]

Supplementary Information

Table S1. Overview of Studies with Mixed Primary Site Gastroenteropancreatic Neuroendocrine Carciomas.

**Author (Year, Country)**	**Time Period**	**P**	**CR**	**S**	**O**	**GB**	**SI**	**A**	**L**	**Other**	**UK**	**GEP-NECs (M:W)**	**Age (range)**	**LD: ED [g] (*n*)**	**Lymph Met. (*n*)**	**Distant Met. (*n*)**	**Ki-67% (Range)**	**SC: LC (*n*)**	***N* Positive: Syn/CgA/ NSE/CD56/ p53**	**OS Months (Range)**
Van der Gaast A. [91] (1990, The Netherlands)	NA	2	0	1	2	0	0	0	0	0	1	6 (NA)	NA	2:4	NA	4	NA	6 [h]:0	NA	(10–22)
Moertel C.G. [114] (1991, USA)	1987–1990	6	3	3	0	0	2	0	0	0	3	17 (NA)	NA	NA	NA	17 [h]	NA	NA	NA	NA
Galanis E. [9] (1997, USA)	1974–1994	7	13	0	8	1	0	0	0	0	0	29 (15:14)	NA	12:17	NA	17	NA	29 [h]:0	NA	M[1]: 5 mo
Kimura H. [14] (1999, Toyama, Japan)	1981–1994	0	2	8	4	0	0	0	0	0	0	14 (12:2)	M[2]: 61 (44–75)	NA	NA	13	NA	14 [h]:0	p53: 8/14	M[1]: 8.4
Mitry E. [88] (1999, France)	1988–1997	13	2	3	0	2	0	0	0	0	NA [d]	20 (NA)	NA	3:17	NA	17	NA	NA	NA	NA
Moyana T.N. [44] (2000, Canada)	1964–NA	NA	NA	NA	NA	NA	NA	NA	NA	NA	NA	5 (NA)	NA	NA	NA	3	NA	NA	NA	NA
Ordónez N.G. [36] (2000, USA)	NA	1	11	0	1	0	0	0	0	0	0	13 (NA)	NA	NA	NA	NA	NA	13 [h]:0	NA	NA

Table S1. *Cont.*

Author (Year, Country)	Time Period	P	CR	S	O	GB	SI	A	L	Other	UK	GEP-NECs (M:W)	Age (range)	LD: ED [g] (*n*)	Lymph Met. (*n*)	Distant Met. (*n*)	Ki-67% (Range)	SC: LC (*n*)	*N* Positive: Syn/CgA/ NSE/CD56/ p53	OS Months (Range)
Cheuk W. [35] (2001, Hong Kong, USA)	NA	1	2	4	7	0	1	0	0	0	0	15 (NA)	NA	NA	NA	NA	NA	15 [h]:0	NA	NA
Fjällskog M.L. [83] (2001, Sweden)	NA	4	0	0	0	0	0	0	0	0	0	4 (NA)	NA	NA	NA	4	NA	NA	NA	NA
Dacic S. [41] (2002, USA)	NA	1	2	2	0	0	0	0	0	0	0	5 (3:2)	M[2]: M: 62, W: 60	NA	NA	NA	NA	5 [h]:0	p53: 5/5	NA
Papotti M. [72] (2002, Italy)	NA	2	2	0	0	0	0	1	0	0	0	5 (3:2)	(20–76)	NA	4	1	NA	NA	NA	(6–72) 1: NA
Cimitan M. [80] (2003, Italy)	NA	NA	4	1	NA	NA	NA	NA	NA	4	0	9 (6:3)	(45–73)	NA	NA	7	NA	9 [h]:0	NA	NA
Fjällskog M.L. [69] (2003, Sweden)	NA	NA	NA	NA	NA	NA	NA	NA	NA	NA	NA	5 (2:3)	(25–64)	NA	NA	5	NA	NA	NA	NA
Pizzi S. [38] (2003, Italy)	NA	0	9	15	0	0	0	0	0	0	0	24 (19:5)	(40–91)	NA	16 (4 NA)	2	(5–80)	NA	p53: 14/24	NA
Brenner B. [7] (2004, USA)	1980–2002	6	25	5	19	5	2	0	0	0	2	64 (31:33)	M[1]: 61 (37–86)	33:30	34	35	NA	64 [h]:0	NA	M[1]: 11 (1–107)
Furlan D. [39] (2004, Italy)	NA	1	7	6	0	0	0		0	0	0	14 (11:3)	(37–77)	NA	16 (2 NA)	4 (2 NA)	(25–70)	NA	NA	(1–72)

Table S1. *Cont.*

Author (Year, Country)	Time Period	P	CR	S	O	GB	SI	A	L	Other	UK	GEP-NECs (M:W)	Age (range)	LD: ED [g] (*n*)	Lymph Met. (*n*)	Distant Met. (*n*)	Ki-67% (Range)	SC: LC (*n*)	*N* Positive: Syn/CgA/ NSE/CD56/ p53	OS Months (Range)
La Rosa S. [58] (2004, Italy)	NA	2	10	7	8	0	0	0	0	0	0	27 (22:5)	M^2: 56–70 [i]	NA	NA	26	NA	NA	NA	NA
Pape U.F. [18] (2004, Germany)	1980–2001	NA	NA	NA	NA	NA	NA	NA	NA	NA	NA	15 (NA)	NA	NA	NA	NA	NA	NA	NA	M^2: 15 (95% CI: 7–22)
Artale S. [107] (2005, Italy)	2000–2003	5	0	0	0	0	0	0	0	0	3	8 (4:4)	M^1: 54 (40–77)	NA	NA	8 [h]	M^2: 30 (7–90)	NA	NA	2-year survival: 66%
Furlan D. [43] (2005, Italy)	NA	1	10	7	2	1	0	0	0	0	0	21 (15:6)	NA	NA	16	7	(26–70)	1:6 (14 mixed)	p53: 11/14	NA
Grabowski P. [45] (2005, Germany)	1981–2001	0	8	2	0	0	0	0	0	0	0	10 (9:1)	M^1: 56 (29–81)	NA	NA	10	(15–NA)	NA	NA	M^1: 13
Pizzi S. [55] (2005, Italy)	NA	0	3	8	0	0	0	0	0	0	0	11 (9:2)	(44–87)	NA	8	3	NA	NA	NA	NA
Shida T. [50] (2005, Japan)	1999–2004	0	6	3	1	0	0	0	0	0	0	10 (5:5)	(45–83)	NA	NA	5	NA	6:4	Syn: 7/10, CgA: 4/10	(1–24)
Haider K. [12] (2006, Canada)	1971–2002	7	8	3	2	0	0	0	0	0	0	20 (13:7)	NA	7:13	NA	NA	NA	20 [h]:0	NA	M^1: 4.4 (0.1–23.5)

Table S1. *Cont.*

Author (Year, Country)	Time Period	P	CR	S	O	GB	SI	A	L	Other	UK	GEP-NECs (M:W)	Age (range)	LD: ED [g] (*n*)	Lymph Met. (*n*)	Distant Met. (*n*)	Ki-67% (Range)	SC: LC (*n*)	*N* Positive: Syn/CgA/NSE/CD56/p53	OS Months (Range)
Hainsworth J.D. [84] (2006, USA)	1999–2005	3	9	1	1	1	0	0	0	0	NA [d]	15 (NA)	NA	NA	NA	15	NA	NA	NA	NA
Ishikubo T. [54] (2006, Japan)	NA	0	4	16	1	0	2	0	0	0	0	23 (19:4)	Average: 60	NA	NA	NA	NA	NA	NA	NA
Kim K.O. [13] (2006, Korea)	1998–2005	5	1	5	6	0	0	0	1	0	0	18 (NA)	NA	11:7	NA	NA	NA	18 [h]:0	NA	M[1]: 8 (95% CI: 6.2–10)
Li A.F.Y. [42] (2006, Taiwan)	1985–2004	0	6	9	6	0	1	0	0	0	0	22 (18:4)	M[2]: 66.1 ± 16.9 (32–88)	NA	18	7	NA	NA	p53: 16/22	M[2]: 20.3 ± 23.1 (1–91)
Uccella S. [37] (2006, Italy)	1982–2004	0	11	11	6	0	0	0	0	0	0	28 (22:6)	(41–85)	NA	NA	(28 "malignant")	NA	NA	NA	NA
Bajetta E. [120] (2007, Italy)	2000–2005	4	NA	0	NA	NA	1	NA	NA	NA	0	5 (NA)	NA	NA	NA	NA	NA	NA	NA	NA
Cho C.S. [283] (2007, USA)	1990–2006	NA	NA	NA	NA	NA	NA	NA	NA	NA	NA	3–7 (NA)	NA	NA	NA	3–7 [h]	NA	NA	NA	M[1]: 6
Cicin I. [75] (2007, Turkey)	1999–2006	2	1	0	0	2	0	0	0	0	0	5 (3:2)	(40–73)	2:3	NA	3	NA	5 [h]:0	NA	(2–68)
Correale P. [59] (2007, Italy) [a]	1999–2003	2	NA	NA	NA	NA	NA	NA	NA	8	NA [d]	10 (NA)	NA	NA	NA	10	NA	NA	NA	NA

Table S1. *Cont.*

Author (Year, Country)	Time Period	P	CR	S	O	GB	SI	A	L	Other	UK	GEP-NECs (M:W)	Age (range)	LD: ED [g] (*n*)	Lymph Met. (*n*)	Distant Met. (*n*)	Ki-67% (Range)	SC: LC (*n*)	*N* Positive: Syn/CgA/ NSE/CD56/ p53	OS Months (Range)
Faggiano, A. [76] (2007, France)	1990–2001	6	1	0	0	1	1	0	0	0	0	9 (NA)	NA	NA	NA	NA	NA	0:9 [h]	NA	NA
Formica V. [8] (2007, UK)	1974–2005	NA	NA	NA	NA	NA	NA	NA	NA	NA	NA	9 (NA)	NA	NA	NA	NA	NA	NA	NA	M[1]: 16
Lee H. [70] (2007, Korea)	1996–2005	0	3	9	0	0	0	0	0	0	0	12 (8:4)	M[2] LC/SC: 53.5/57 (38–77)	NA	10	5	M[2] LC/SC: 59.6/56	6:6	Syn: 8/9, CgA: 3/9, p53: 5/9	NA
Lee S.S. [15] (2007, Korea)	1995–2004	1	5	3	7	15	1	0	2	0	0	34 (NA)	NA	15:19	NA	NA	NA	34 [h]:0	NA	M[1]: 10 (95% CI: 5–15)
Lepage C. [1] (2007, all of UK)	1986–1999	68	95	112	557	10	8	0	21	0	0	871 (359: 512)	M[2]: 70.6 (SD 11.4)	NA	NA	NA	NA	871 [h]:0	NA	1-year survival: 0.3%–28.2% [i]
Lin Y.L. [17] (2007, Taiwan)	1995–2005	NA	NA	NA	NA	NA	NA	NA	NA	NA	NA	27 (NA)	NA	7:20	NA	NA	NA	27 [h]:0	NA	M[1]: 6.4 (1.8–11.0)
Ploeckinger U. [73] (2007, Germany)	1999–NA	28	14	1	3	0	13	0	0	0	NA [d]	59 (NA)	NA	NA	NA	NA	NA	NA	NA	NA

Table S1. *Cont.*

Author (Year, Country)	Time Period	P	CR	S	O	GB	SI	A	L	Other	UK	GEP-NECs (M:W)	Age (range)	LD: ED [g] (*n*)	Lymph Met. (*n*)	Distant Met. (*n*)	Ki-67% (Range)	SC: LC (*n*)	*N* Positive: Syn/CgA/ NSE/CD56/ p53	OS Months (Range)
Ceppi P. [47] (2008, Italy)	1992–2006	NA	NA	NA	NA	NA	NA	NA	NA	NA	NA	8 (4:4)	M[2]: 45 (30–85)	NA	NA	NA	NA	NA	NA	NA
Grabowski P. [46] (2008, Germany)	1981–2001	0	8	2	0	0	0	0	0	0	0	10 (9:1)	M[1]: 56 (29–81)	NA	NA	10	NA	NA	NA	M[1]: 8 (1–15)
Le Treut Y.P. [130] (2008, France)	1989–2005	NA	NA	NA	NA	NA	NA	NA	NA	NA	NA	0–11 (NA)	NA	NA	NA	0–11 [h]	NA	NA	NA	M[1]: 10
Shia, J. [29] (2008, USA)	NA	0	40	18	6	0	0	1	0	0	0	65 (36:29)	M[1]: 64 (30–91)	NA	NA	22 (7 NA)	NA	23:33 (+ 11 mixed)	Syn: 57/65, CgA: 42/65, CD56: 47/65	M[2]: 15.7 (95% CI: 11.3–19.7)
Yao J.C. [19] (2008, USA)	1973–2004	NA	NA	NA	NA	NA	NA	NA	NA	NA	NA	4054 (NA)	NA	NA	NA	50%	NA	NA	NA	M[1]: 10 (95% CI: 9–11)
Frilling A. [128] (2009, Germany)	1992–2006	NA	NA	NA	NA	NA	NA	NA	NA	NA	NA	58–82 (NA)	NA	NA	NA	58–82 [h]	NA	NA	NA	NA

Table S1. *Cont.*

Author (Year, Country)	Time Period	P	CR	S	O	GB	SI	A	L	Other	UK	GEP-NECs (M:W)	Age (range)	LD: ED [g] (*n*)	Lymph Met. (*n*)	Distant Met. (*n*)	Ki-67% (Range)	SC: LC (*n*)	*N* Positive: Syn/CgA/ NSE/CD56/ p53	OS Months (Range)
Lepage C. [79] (2009, EUROCARE database)	1985–1994	109	114	123	419	48 [j]	7	0	0	0	0	820 (NA)	NA	NA	NA	NA	NA	820 [h]: 0	NA	5-year survival relative survival: 8.1 (7.4–11.3)
Lombard-Bohas C. [71] (2009, France)	NA	NA	NA	NA	NA	NA	NA	NA	NA	NA	NA	68 (NA)	NA	NA	NA	NA	NA	NA	NA	NA
Shida T. [51] (2009, Japan)	1999–2007	2	6	3	1	1	1	0	0	0	0	14 (6:8)	(19–83)	NA	NA	10	(18–82)	8:6	Syn: 10/14, CgA: 7/14	(1–48)
Shida T. [56] (2009, Japan)	1999–2007	3	6	0	0	0	0	0	0	0	0	9 (4:5)	(19–84)	NA	NA	5	NA	3:6	NA	(2–65)
Srirajaskanthan R. [105] (2009, UK)	NA	3	1	1	0	0	1	0	0	0	NA [d]	6 (NA)	NA	NA	NA	NA	NA	NA	NA	NA
Wong Y.N. [65] (2009, England)	1970–2004	55	60	92	293	15	NA	NA	NA	17	NA	532 (231: 301)	NA	181: 159 (192 NA)	NA	159 (192 NA)	NA	532 [h]:0	NA	3-year survival: 7%

Table S1. *Cont.*

Author (Year, Country)	Time Period	P	CR	S	O	GB	SI	A	L	Other	UK	GEP-NECs (M:W)	Age (range)	LD: ED [g] (*n*)	Lymph Met. (*n*)	Distant Met. (*n*)	Ki-67% (Range)	SC: LC (*n*)	*N* Positive: Syn/CgA/ NSE/CD56/ p53	OS Months (Range)
Binderup T. [111] (2010, Denmark)	2007–2008	NA	NA	NA	NA	NA	NA	NA	NA	NA	NA	14 [c,*] (NA)	NA	NA	NA	NA	NA	NA	NA	NA
Brennan S.M. [6] (2010, Australia)	1985–2007	NA	NA	NA	20	NA	NA	NA	NA	8	NA	28 (NA)	NA	16:12	NA	NA	NA	28 [h]:0	NA	M[1]: 8.4
Catena L. [57] (2010, Italy)	1984–2009	12	6	0	0	0	4	0	0	0	NA [d]	30 (NA)	M[1]: 59 (17–75)	NA	NA	30	NA	NA	NA	NA
Garcia-Carbonero R. [10] (2010, Spain)	2001–2008	NA	NA	NA	NA	NA	NA	NA	NA	NA	NA	54 (34:20)	NA	NA	NA	32	NA	NA	NA	M[1]: 14.4
Gregory D.L. [77] (2010, Australia)	1996–2007	0	1	1	5	0	0	0	0	0	0	7 (NA)	NA	6:1	NA	1	NA	7 [h]:0	NA	NA
Li A.F.Y. [16] (2010, Taiwan)	1989–2008	0	13	8	17	0	1	0	0	0	NA [d]	39 (35:5)	M[2]: 61.8–84 [i]	14:23 (2 NA)	NA	23	NA	39 [h]:0	NA	M[1]: 6–15 (±4.46–49.97) [i]
Li A.F.Y. [34] (2010, Taiwan)	1989–2009	0	15	15	11	1	0	0	0	0	0	42 (37:5)	M[1]: 64 (32–84)	15:27	NA	27	NA	42 [h]:0	Syn: 42/42, CgA: 26/42, NSE: 42/42, CD56: 38/42	M[1]: 16 ± 21 mo

Table S1. *Cont.*

Author (Year, Country)	Time Period	P	CR	S	O	GB	SI	A	L	Other	UK	GEP-NECs (M:W)	Age (range)	LD: ED [g] (*n*)	Lymph Met. (*n*)	Distant Met. (*n*)	Ki-67% (Range)	SC: LC (*n*)	*N* Positive: Syn/CgA/NSE/CD56/p53	OS Months (Range)
Niederle M.B. [66] (2010, Austria) [a]	2004–2005	5	8	2	0	0	2	1	1	0	0	19 (NA)	M[1]: 73	NA	NA	NA	NA	NA	NA	NA
O'Toole D. [40] (2010, Ireland)	NA	NA	NA	NA	NA	NA	NA	NA	NA	NA	NA	13 (NA)	NA	NA	NA	NA	NA	NA	NA	M[1]: 13 (0–112)
Saxena A. [129] (2010, Australia) [a]	2003–2009	NA	NA	NA	NA	NA	NA	NA	NA	NA	NA	(5–8) (NA)	NA	NA	NA	5–8 [h]	NA	NA	NA	NA
Turner N.C. [119] (2010, UK) [a]	1999–2008	5	0	0	0	0	0	0	0	NA	0	5 (NA)	NA	NA	NA	NA	NA	NA	NA	NA
Deutschbein T. [116] (2011, Germany)	2005–2009	2	2	4	1	0	0	0	0	0	3	12 (7:5)	(39–75)	NA	7	11	(25–85)	NA	NA	NA
Ezziddin S. [109] (2011, Germany)	NA	3	NA	NA	NA	NA	NA	NA	NA	4	0	7 (4:3)	NA	NA	NA	7	NA	NA	NA	NA
Grossman R.A. [11] (2011, USA)	1973–2006	NA	342	147	274	NA	13	NA	NA	456	0	1232 (NA)	NA	NA	NA	722	NA	1232 [h]:0	NA	M[1]: 4
Hentic O. [100] (2011, France)	1997–2006	8	0	1	0	0	1	0	0	0	8	18 (7:11)	M[1]: 58 (33–71)	NA	NA	18 [h]	(25–90)	NA	NA	M[1]: 14

Table S1. *Cont.*

**Author (Year, Country)**	**Time Period**	**P**	**CR**	**S**	**O**	**GB**	**SI**	**A**	**L**	**Other**	**UK**	**GEP-NECs (M:W)**	**Age (range)**	**LD: ED [g] (*n*)**	**Lymph Met. (*n*)**	**Distant Met. (*n*)**	**Ki-67% (Range)**	**SC: LC (*n*)**	***N* Positive: Syn/CgA/ NSE/CD56/ p53**	**OS Months (Range)**
Jin S. [86] (2011, China)	2004–2008	1	2	2	4	0	0	0	0	0	0	9 (NA)	NA	NA	NA	9	NA	9 [h]:0	NA	NA
Oh S. [110] (2011, Korea)	NA	NA	NA	NA	NA	NA	NA	NA	NA	NA	NA	6 (NA)	NA	NA	NA	6	NA	NA	NA	NA
Sclafani F. [74] (2011, Italy)	1997–2007	2	2	1	0	0	1	0	0	0	0	6 (3:3)	(34–78)	NA	NA	NA	NA	NA	NA	NA
Welin S. [108] (2011, Sweden, Norway)	2004–2009	10	5	2	0	0	0	0	0	0	NA [d]	17 (NA)	NA	NA	NA	17	NA	NA	NA	NA
Cho M.Y. [2] (2012, Korea)	2000–2009	NA	NA	NA	NA	NA	NA	NA	NA	NA	NA	72 (NA)	NA	NA	NA	NA	NA	NA	NA	5-year survival: 42.96%
Faggiano A. [68] (2012, Italy)	1990–2007	19	7	7	0	0	2	0	0	0	NA [d]	35 (NA)	NA	NA	NA	NA	NA	NA	NA	NA
Hentic O. [85] (2012, France)	2000–2010	10	2	0	0	0	0	0	6	1	0	19 (7:12)	NA	NA	NA	18	M[l]: 50 (21–100)	11:6 (2NA)	CgA: 14/17	M[l]: 14 (1.6–30)
Knösel T. [53] (2012, Germany)	NA	NA	NA	NA	NA	NA	NA	NA	NA	NA	NA	29 (NA)	NA	NA	NA	NA	NA	NA	NA	5-year survival: 31% ± 9%

Table S1. *Cont.*

Author (Year, Country)	Time Period	P	CR	S	O	GB	SI	A	L	Other	UK	GEP-NECs (M:W)	Age (range)	LD: ED [g] (*n*)	Lymph Met. (*n*)	Distant Met. (*n*)	Ki-67% (Range)	SC: LC (*n*)	*N* Positive: Syn/CgA/ NSE/CD56/ p53	OS Months (Range)
Korse C.M. [95] (2012, The Netherlands)	1994–2009	9	5	0	0	0	0	0	0	0	NA [d]	14 (NA)	NA	NA	NA	NA	NA	2:12	NA	NA
Nakano K. [118] (2012, Japan)	2005–2011	NA	NA	NA	NA	NA	NA	NA	NA	NA	NA [d]	9 (NA)	NA	NA	NA	NA	NA	NA	NA	NA
Olsen I.H. [117] (2012, Denmark)	2007–2009	4	6	0	3	0	2	0	0	0	NA [d]	15 (NA)	NA	NA	NA	15	(20–100)	NA	NA	NA
Olsen I.H. [122] (2012, Denmark)	NA	7	5	3	3	0	0	0	0	0	NA [d]	18 (NA)	NA	NA	NA	18	(20–NA)	NA	NA	NA
Quinn A.M. [78] (2012, Manchester, UK)	1994–2003	0	1	2	3	0	0	0	0	0	0	6 (NA)	NA	NA	NA	NA	NA	5 [h]:0, 1 NA	Syn: 5/6	NA
Terashima T. [115] (2012, Japan)	2000–2008	9	0	10	8	4	0	0	3	0	NA [d]	34 (23:11)	M[1]: 46.5–63.5 (27–79) [i]	0:34 [h]	18	34 [h] (ED)	NA	34 [h]:0	NA	M[1]: 7.8–14.9 (±1.49–14.9) [i]

Table S1. *Cont.*

Author (Year, Country)	Time Period	P	CR	S	O	GB	SI	A	L	Other	UK	GEP-NECs (M:W)	Age (range)	LD: ED [g] (*n*)	Lymph Met. (*n*)	Distant Met. (*n*)	Ki-67% (Range)	SC: LC (*n*)	*N* Positive: Syn/CgA/ NSE/CD56/ p53	OS Months (Range)
Ahn H.K. [123] (2013, Korea)	2010–2012	NA	NA	NA	NA	NA	NA	NA	NA	NA	NA	13 (NA)	NA	NA	NA	NA	NA	NA	NA	NA
Delektorskaya V.V. [67] (2013, Russia)	1994–2011	8	8	4	1	0	2	0	0	0	0	23 (NA)	NA	NA	14	12	(23–80)	4:4	p53: 12/23	NA
Du Z. [81] (2013, China)	2008–2010	0	3	6	0	0	1	1	0	0	0	11 (9:2)	M[1]: 53 (35–74)	NA	5	7	(40–90)	NA	NA	M[1]: 13 (6–40)
Feng S.T. [82] (2013, China)	2009–2013	0	5	16	7	0	3	0	0	0	0	31 (22:9)	M[2]: 60.7 (±12.1)	NA	28	13	NA	NA	NA	NA
Ferrarotto R. [121] (2013, Brazil)	2003–2011	NA	NA	NA	NA	NA	NA	NA	NA	NA	NA	(3–9) (NA)	NA	NA	NA	(3–9)	NA	NA	NA	NA
Galván J.A. [49] (2013, Spain)	2000–2009	NA	NA	NA	NA	NA	NA	NA	NA	NA	NA	25 (NA)	NA	NA	NA	NA	(20–NA) (1 < 20)	20:5	-	NA
Korse C.M. [3] (2013, The Netherlands) [b]	1990–2010	433	372	203	336	NA	97	0	NA	112	0	1553 (NA)	NA	NA	NA	594 (626 NA)	NA	NA	NA	5 year relative survival: LC 19%–44% [i] SC 0–12% [i]
Lee H.S. [48] (2013, Korea)	NA	NA	NA	NA	NA	NA	NA	NA	NA	NA	NA	32 (NA)	NA	NA	NA	NA	NA	NA	NA	NA

Table S1. *Cont.*

Author (Year, Country)	Time Period	P	CR	S	O	GB	SI	A	L	Other	UK	GEP-NECs (M:W)	Age (range)	LD: ED [g] (*n*)	Lymph Met. (*n*)	Distant Met. (*n*)	Ki-67% (Range)	SC: LC (*n*)	*N* Positive: Syn/CgA/ NSE/CD56/ p53	OS Months (Range)
Lu Z.H. [87] (2013, China)	2009–2011	1	0	8	5	0	1	0	0	0	1	18 [e] (13:3)	M[1]: 60 (31–66)	NA	NA	15	NA	15:1	Syn: 15/16, CgA: 7/12, NSE: 3/4, CD56: 12/13	M[1]: 10.6
Mia-Jan K. [52] (2013, Korea)	2000–2012	1	3	5	0	2	2	0	10	0	0	23 (NA)	NA	NA	NA	10	NA	NA	NA	NA
Ozkara, S. [26] (2013, Turkey)	2004–2012	NA	NA	NA	NA	NA	NA	NA	NA	NA	NA	17 (NA)	NA	NA	NA	NA	NA	NA	Syn: 16/17, CgA: 11/17	NA
Sorbye H. [4] (2013, 12 Nordic hospitals)	2000–2009	71	82	20	12	NA	NA	NA	NA	20	98 (2 NA)	305 (154: 151)	M[1]: 60 (24–89)	NA	188	301	(136 < 55 154 ≥ 55%)	117: 154 (30 NA)	Syn: 291/303, CgA: 286/303	M[1]: 1 or 11 (+/− chemo) (95% CI: 0.3–1.8/ 9.4–12.6)
Vélayoudom-Céphise F.L. [90] (2013, France)	2000–2005	9	1	NA	NA	NA	NA	NA	NA	NA	NA [d]	10 (NA)	NA	NA	NA	NA	(20–NA)	0:10 [h]	NA	NA
Van der Zwan J.M. [284] (2013 RARECARE)	1995–2002	128	172	143	NA	NA	24	2	NA	NA	NA	NA	NA	NA	NA	NA	NA	NA	NA	NA

Table S1. *Cont.*

Author (Year, Country)	Time Period	P	CR	S	O	GB	SI	A	L	Other	UK	GEP-NECs (M:W)	Age (range)	LD: ED [g] (*n*)	Lymph Met. (*n*)	Distant Met. (*n*)	Ki-67% (Range)	SC: LC (*n*)	*N* Positive: Syn/CgA/ NSE/CD56/ p53	OS Months (Range)
Olsen I.H. [124] (2014, Denmark)	NA	2	1	0	8	0	2	0	0	0	NA [d]	13 (NA)	NA	NA	NA	13	NA	NA	NA	NA
Yamaguchi T. [92] (2014, Japan)	2000–2011	35	31	70	85	31 [k]	6	0	NA	0	0	258 [f] (182:76)	M[1]: 62.5 (26–81)	NA	131	219	(43 >55%, 27:20–54%) (188 NA)	122: NA	Syn: 204/258, CgA: 172/258	M[1]: 11.5
TOTAL [c]	-	**744/ 4904**	**1122/ 4904**	**702/ 4904**	**1368/ 4904**	**66/ 4904**	**154/ 4904**	**4/ 4904**	**41/ 4904**	**601/ 4904**	**102/ 4904**	**7988 (989:890 /1879)**	-	-	-	-	-	-	-	-

[a] Prospective study, all other studies are retrospective or not stated; [b] Might not be exact numbers: extrapolated from incidence rates; [c] Total after removal of possible overlapping cohorts (*i.e.*, if cohorts are from the same hospital and if it is not clearly stated in the article that patients have not been used in previous studies); [d] Can have a number of unknown NECs in a mixed location cohort but without specification of gastroenteropancreatic metastases; [e] Only information for 16 patients; [f] 21 MANECs included; [g] LD (Limited Disease): confined to a localized anatomic region/organ of origin with locoregional lymph node involvement, (encompassable within one (tolerable) radiation therapy treatment port/radiation field), ED (Extensive Disease): beyond LD; [h] Part of inclusion criteria; [i] Variation due to no group value, range of values from different primary sites; [j] includes liver and gallbladder (distribution NA); [k] hepatobiliary system (distribution NA); GEP-NEC: Gastroenteropancreatic neuroendocrine carcinoma (includes WHO 2010 definition, WHO 2000/2004 definition; poorly differentiated endocrine carcinomas (PDEC), small cell carcinomas, large cell carcinomas and anaplastic/high grade carcinomas); Other NEC: neuroendocrine carcinoma/poorly differentiated carcinoma/small cell carcinoma of sites other than the gastroenteropancreatic system; NET: neuroendocrine tumour/well differentiated endocrine tumour/well differentiated carcinoma/low grade tumour; NA: Not Available (if the study has a larger cohort with locations other than the GEP-system, with values for the whole group but none specific for GEP-NECs, these values are marked as NA); P: N Pancreas, CR: N Colorectum, S: N Stomach, O: N Oesophagus, GB: N Gallbladder, SI: N Small Intestine, A: N Appendix, L: N Liver, UK: N Unknown; M: N Men, W: N Women; M[1]: Median, M[2]: Mean; LC: Large Cell, SC: Small Cell; Syn: Synaptophysin, CgA: Chromogranin A, NSE: Neuron Specific Enolase; OS: Overall Survival; Met.: Metastases.

Table S2. Overview of Immunohistochemistry and Molecular Markers.

Author (Year, Country)	Time Period	*n* GEP-NEC ([a])	Ki-67% (Range)	*n* SC: *n* LC	IHC: *n* syn Positive	IHC: *n* CgA Positive	IHC: *n* NSE Positive	IHC: *n* CD56 Positive	IHC: *n* p53 Positive	IHC: *n* p16 Positive	IHC: *n* TTF-1 Positive	IHC: *n* TS Positive	IHC: *n* mTOR Positive	Other *n* IHC Positive	MA: *n* LOH or AI p53	MA: *n* LOH or AI p3	MA: *n* LOH or AI MEN-1	MA: *n* LOH or AI other	Other
Moertel C.G. [114] (1991, USA)	1987–1990	17 (18 [b])	NA	NA:NA	2/4 [b] (50%)	2/4 [b] (50%)	2/4 [b] (50%)	-	-	-	-	-	-	-	-	-	-	-	-
Kimura H. [14] (1999, Japan)	1981–1994	14	PCNA: 72 ± 11.6 (53–91)	14 [d]:0	-	-	-	-	8/14 (57%)	-	-	-	-	-	-	-	-	-	-
Moyana T.N. [44] (2000, Canada)	1964–NA	5 (58 [c])	“extensive areas of positivity”	NA:NA	-	-	-	-	7/58 [c] (12%)	-	-	-	-	-	-	-	-	-	-
Ordónez N.G. [36] (2000, USA)	NA	13	NA	13 [d]:0	-	-	-	-	-	-	3/13 (23%)	-	-	CK-20: 0/13 (0%)	-	-	-	-	-
Cheuk W. [35] (2001, Hong Kong, USA)	NA	15	NA	15 [d]:0	-	-	-	-	-	-	8/15 (53%)	-	-	CK-20: 0/15 (0%)	-	-	-	-	-

Table S2. *Cont.*

Author (Year, Country)	Time Period	*n* GEP-NEC ([a])	Ki-67% (Range)	*n* SC: *n* LC	IHC: *n* syn Positive	IHC: *n* CgA Positive	IHC: *n* NSE Positive	IHC: *n* CD56 Positive	IHC: *n* p53 Positive	IHC: *n* p16 Positive	IHC: *n* TTF-1 Positive	IHC: *n* TS Positive	IHC: *n* mTOR Positive	Other *n* IHC Positive	MA: *n* LOH or AI p53	MA: *n* LOH or AI p3	MA: *n* LOH or AI MEN-1	MA: *n* LOH or AI other	Other
Dacic S. [41] (2002, USA)	NA	5	NA	5:0	-	-	-	-	-	-	-	-	-	-	4/4 (100%)	-	-	P16: 4/7 [i] (57%), L-myc: 5/5, PTEN: 4/4	-
Pizzi S. [38] (2003, Italy)	NA	24	(5–80)	NA:NA	-	-	-	-	14/24 (58%)	14/24 (58%)	-	-	-	Loss of Rb: 13/24 (54%)	18/22 (82%)	13/23 (57%)	11/23 (48%)	11q: 9/14, 18q: 11/23	-
Brenner B. [7] (2004, USA)	1980–2002	64	NA	64 [d]:0	-	-	-	-	-	-	-	-	-	-	-	-	-	-	-
Furlan D. [39] (2004, Italy)	NA	14	(25–70)	NA:NA	-	-	-	-	-	-	-	-	-	-	12/13 (92%)	5/13 (38%)	5/10 (50%)	-	-
La Rosa S. [58] (2004, Italy)	NA	27	NA	NA:NA	-	-	-	-	-	-	-	-	-	Nuclear CDX2: 3/27 (11%)	-	-	-	-	-

Table S2. *Cont.*

Author (Year, Country)	Time Period	*n* GEP-NEC ([a])	Ki-67% (Range)	*n* SC: *n* LC	IHC: *n* syn Positive	IHC: *n* CgA Positive	IHC: *n* NSE Positive	IHC: *n* CD56 Positive	IHC: *n* p53 Positive	IHC: *n* p16 Positive	IHC: *n* TTF-1 Positive	IHC: *n* TS Positive	IHC: *n* mTOR Positive	Other *n* IHC Positive	MA: *n* LOH or AI p53	MA: *n* LOH or AI p3	MA: *n* LOH or AI MEN-1	MA: *n* LOH or AI other	Other
Furlan D. [43] (2005, Italy)	NA	21 (36 [b])	(26–70)	1:6 (14 mixed)	-	-	-	-	11/14 (79%)	-	-	-	-	-	19/20 (95%)	9/18 (50%)	-	-	-
Grabowski P. [45] (2005, Germany)	1981–2001	10	(>15–NA)	NA:NA	-	-	-	-	-	-	-	-	-	Survivin1: 10/10 (100%)	-	-	-	-	-
Pizzi S. [55] (2005, Italy)	NA	11	NA	NA:NA	-	-	-	-	-	-	-	-	-	Hyper-expression CyD1: 2/11 (18%)	-	4/9 (44%)	-	-	RASSF1 A methylation: 2/11 (18%)
Shida T. [50] (2005, Japan)	1999–2004	10	NA	6:4	7/10 (70%)	4/10 (40%)	-	-	-	-	-	-	-	-	-	-	-	-	mRNA: 8/10 intense hASH1, 1/10 mild hASH1
Ishikubo T. [54] (2006, Japan)	NA	23	NA	NA:NA	-	-	-	-	-	-	-	-	-	c-kit: 6/23 (26%)	-	-	-	-	-

Table S2. *Cont.*

Author (Year, Country)	Time Period	*n* GEP-NEC ([a])	Ki-67% (Range)	*n* SC: *n* LC	IHC: *n* syn Positive	IHC: *n* CgA Positive	IHC: *n* NSE Positive	IHC: *n* CD56 Positive	IHC: *n* p53 Positive	IHC: *n* p16 Positive	IHC: *n* TTF-1 Positive	IHC: *n* TS Positive	IHC: *n* mTOR Positive	Other *n* IHC Positive	MA: *n* LOH or AI p53	MA: *n* LOH or AI p3	MA: *n* LOH or AI MEN-1	MA: *n* LOH or AI other	Other
Li A.F.Y. [42] (2006, Taiwan)	1985–2004	22	NA	NA:NA	-	-	-	-	16/22 (73%)	17/22 (77%)	-	-	-	c-kit: 0/22 (0%)	-	-	-	-	-
Uccella S. [37] (2006, Italy)	1982–2004	28	NA	NA:NA	-	-	-	-	-	-	-	-	-	VMAT2: 17/28 (61%) HDC: 13/22 (59%)	-	-	-	-	-
Lee H. [70] (2007, Korea)	1996–2005	12	Mean large cell: 59.6, mean small cell: 56	6:6	8/9 (89%)	3/9 (33%)	-	-	5/9 (56%)	-	0/9 (0%)	-	-	Gastrin: 0/9 (0%) SS: 0/9 (0%)	-	-	-	-	-
Ceppi P. [47] (2008, Italy)	1992–2006	8	NA	NA:NA	-	-	-	-	-	-	-	6/8 (75%)	-	-	-	-	-	-	TS mRNA: median 18.7 (3–49.5)
Grabowski P. [46] (2008, Germany)	1981–2001	10	NA	NA:NA	-	-	-	-	-	-	-	-	-	High cyclin E: 7/19 (37%) High p27: 0/27 (0%)	-	-	-	-	PCR: 1/10 (10%) p53 mutation

Table S2. *Cont.*

**Author (Year, Country)**	**Time Period**	***n* GEP-NEC ([a])**	**Ki-67% (Range)**	***n* SC: *n* LC**	**IHC: *n* syn Positive**	**IHC: *n* CgA Positive**	**IHC: *n* NSE Positive**	**IHC: *n* CD56 Positive**	**IHC: *n* p53 Positive**	**IHC: *n* p16 Positive**	**IHC: *n* TTF-1 Positive**	**IHC: *n* TS Positive**	**IHC: *n* mTOR Positive**	**Other *n* IHC Positive**	**MA: *n* LOH or AI p53**	**MA: *n* LOH or AI p3**	**MA: *n* LOH or AI MEN-1**	**MA: *n* LOH or AI other**	**Other**
Shia, J. [29] (2008, USA)	NA	65	NA	23:31 (+11 mixed)	56/65 (87%)	42/65 (65%)	-	47/65 (72%)	-	-	-	-	-	-	-	-	-	-	-
Shida T. [51] (2009, Japan)	1999–2007	14	(18–82)	8:6	10/14 (71%)	7/14 (50%)	-	-	-	-	-	-	-	Mash1: 0/14 (0%), Neuro D: 11/14 (79%)	-	-	-	-	-
Shida T. [56] (2009, Japan)	1999–2007	9	NA	3:6	-	-	-	-	-	-	-	-	6/9 (67%)	-	-	-	-	-	-
Catena L. [57] (2010, Italy)	1984–2009	30	NA	NA:NA	-	-	-	-	-	-	-	-	80%	-	-	-	-	-	-
Li A.F.Y. [16] (2010, Taiwan)	1989–2009	42	NA	42 [d]:0	42/42 (100%)	9/41 (22%)	42/42 (100%)	26/42 (62%)	-	-	9/41 (22%)	-	-	CK8: 39/42 (93%), AE1/AE3: 32/42 (76%), EMA: 30/42 (71%)	-	-	-	-	-

Table S2. *Cont.*

Author (Year, Country)	Time Period	*n* GEP-NEC ([a])	Ki-67% (Range)	*n* SC: *n* LC	IHC: *n* syn Positive	IHC: *n* CgA Positive	IHC: *n* NSE Positive	IHC: *n* CD56 Positive	IHC: *n* p53 Positive	IHC: *n* p16 Positive	IHC: *n* TTF-1 Positive	IHC: *n* TS Positive	IHC: *n* mTOR Positive	Other *n* IHC Positive	MA: *n* LOH or AI p53	MA: *n* LOH or AI p3	MA: *n* LOH or AI MEN-1	MA: *n* LOH or AI other	Other
Welin S. [108] (2011, Sweden, Norway)	2004–2009	17 (25 [b])	(>20–NA)	NA:NA	23/23 [b] (100%)	22/24 (92%) [b]	-	-	-	-	-	-	-	-	-	-	-	-	-
Hentic O. [85] (2012, France)	2000–2010	19	Median: 50 (21–100)	11:6 (2 NA)	-	14/17 (82%)	-	-	-	-	-	-	-	-	-	-	-	-	-
Knösel T. [53] (2012, Germany)	NA	29	NA	NA:NA	-	-	-	-	-	-	-			High KIT expression: 28%, PDGFRA: 50%	-	-	-	-	-
Nakano K. [118] (2012, Japan)	2005–2011	9 (44 [b])	NA	NA:NA	39/43 [b] (91%)	30/41 [b] (73%)	-	7/42 [b] (88%)	-	-	8/36 [b] (22%)	-	-	-	-	-	-	-	-
Olsen I.H. [117] (2012, Denmark)	2007–2009	15 (31 [b])	(20–100)	NA:NA	30/31 [b] (97%)	25/31 [b] (81%)	-	-	-	-	-	-	-	-	-	-	-	-	-
Olsen I.H. [122] (2012, Denmark)	NA	18 (28 [b])	Median: 50 [b] (>20–NA)	NA:NA	28/28 [b] (100%)	25/28 [b] (89%)	-	-	-	-	-	-	-	-	-	-	-	-	-

Table S2. *Cont.*

Author (Year, Country)	Time Period	*n* GEP-NEC ([a])	Ki-67% (Range)	*n* SC: *n* LC	IHC: *n* syn Positive	IHC: *n* CgA Positive	IHC: *n* NSE Positive	IHC: *n* CD56 Positive	IHC: *n* p53 Positive	IHC: *n* p16 Positive	IHC: *n* TTF-1 Positive	IHC: *n* TS Positive	IHC: *n* mTOR Positive	Other *n* IHC Positive	MA: *n* LOH or AI p53	MA: *n* LOH or AI p3	MA: *n* LOH or AI MEN-1	MA: *n* LOH or AI other	Other
Quinn A.M. [78] (2012, UK)	1994–2003	6	NA	5:0 (1 NA)	5/6 (83%)	-	-	-	-	-	3/6 (50%)	-	-	34βE12: 0/7 (0%)	-	-	-	-	-
Delektorskaya V.V. [67] (2013, Russia)	1994–2011	23	(23–80)	4:4 (15 NA)	-	-	-	-	12/23 (52%)	-	1/4 (25%)	-	-	CDX-2: 5/8 (63%)	-	-	-	-	-
Lee H.S. [48] (2013, Korea)	NA	32	NA	NA:NA	-	-	-	-	-	-	-	30/32 (94%)	-	E2F1: 22/32 (69%), Rb: 17/32 (53%), p18: 6/32 (19%), p21: 24/32 (75%), p27: 14/32 (44%), menin: 29/32 (91%)	-	-	-	-	-

Table S2. *Cont.*

Author (Year, Country)	Time Period	*n* GEP-NEC ([a])	Ki-67% (Range)	*n* SC: *n* LC	IHC: *n* syn Positive	IHC: *n* CgA Positive	IHC: *n* NSE Positive	IHC: *n* CD56 Positive	IHC: *n* p53 Positive	IHC: *n* p16 Positive	IHC: *n* TTF-1 Positive	IHC: *n* TS Positive	IHC: *n* mTOR Positive	Other *n* IHC Positive	MA: *n* LOH or AI p53	MA: *n* LOH or AI p3	MA: *n* LOH or AI MEN-1	MA: *n* LOH or AI other	Other
Lu Z.H. [87] (2013, China)	2009–2011	18 [e]	NA	15:1	15/16 (94%)	7/12 (58%)	3/4 (75%)	12/13 (92%)	-	-	-	-	-	-	-	-	-	-	-
Mia-Jan K. [52] (2013, Korea)	2000–2012	23	NA	NA:NA	-	-	-	-	-	-	-	-	-	6/23 (26%) CD133	-	-	-	-	-
Ozkara, S. [26] (2013, Turkey)	2004–2012	17	NA	NA:NA	16/17 (94%)	11/17 (65%)	-	-	-	-	-	-	-	-	-	-	-	-	-
Sorbye H. [4] (2013, 12 Nordic hospitals)	2000–2009	305	(20–NA)	117:154	291/303 (96%)	286/303 (94%)	-	-	-	-	-	-	-	-	-	-	-	-	-
Vélayoudom-Céphise F.L. [90] (2013, France)	2000–2005	22 (28 [b])	Median 21–30 [b] (21–90)	0:22 [d]	23/28 [b] (82%)	23/28 [b] (82%)	23/28 [b] (82%)	-	-	-	-	-	-	-	-	-	-	-	-
Olsen I.H. [124] (2014, Denmark)	NA	13 (22 [b])	Median: 95 (20–NA)	NA:NA	21/22 [b] (95%)	19/21 [b] (90%)	-	-	-	-	-	-	-	-	-	-	-	-	-

Table S2. *Cont.*

Author (Year, Country)	Time Period	*n* GEP-NEC ([a])	Ki-67% (Range)	*n* SC: *n* LC	IHC: *n* syn Positive	IHC: *n* CgA Positive	IHC: *n* NSE Positive	IHC: *n* CD56 Positive	IHC: *n* p53 Positive	IHC: *n* p16 Positive	IHC: *n* TTF-1 Positive	IHC: *n* TS Positive	IHC: *n* mTOR Positive	Other n IHC Positive	MA: *n* LOH or AI p53	MA: *n* LOH or AI p3	MA: *n* LOH or AI MEN-1	MA: *n* LOH or AI other	Other
Yamaguchi T. [92] (2014, Japan)	2000–2011	258 [f]	(>20–NA) [g]	122:NA	204/258 (79%)	172/258 (67%)	-	-	-	-	-	-	-	-	-	-	-	-	-
TOTAL	-	-	-	-	**672/762 (88%) [b,h] 647/730 (89%) [h]**	**576/768 (75%) [b,h] 551/736 (75%) [h]**	**77/120 (64%) [b,h] 45/46 [h] (98%)**	**85/119 (71%)**	**73/164 (45%) [c,h] 66/106 [h] (62%)**	**31/46 (67%)**	**32/124 (26%) [b] 24/116 (21%)**	**36/40 (90%)**	**6/9 (67%) + 80% (n NA)**	-	**41/46 (89%) [h]**	**22/41 (54%) [h]**	**16/33 (48%)**	-	-

GEP-NEC: Gastroenteropancreatic neuroendocrine carcinoma (includes WHO 2010 definition, WHO 2000/2004 definition; poorly differentiated endocrine carcinomas (PDEC), small cell carcinomas, large cell carcinomas and anaplastic/high grade carcinomas); Other NEC: neuroendocrine carcinoma/poorly differentiated carcinoma/small cell carcinoma of sites other than the gastroenteropancreatic system; NET: neuroendocrine tumour/well differentiated endocrine tumour/well differentiated carcinoma/low grade tumour; NA: Not Available; SC: Small Cell; LC: Large Cell; PCNA: proliferating cell nuclear antigen; IHC: Immunohistochemistry; Syn: synatophysin; CgA: Chromogranin A; NSE: neuron specific enolase; TTF-1: Thyroid transcription factor 1; TS: Thymidylate synthase; SS: Somatostatin; MA: Microsatellite analysis; LOH: Loss of heterozygosity; AI: Allelelic Imballance; [a] includes GEP-NEC + other NEC ([b]) and/or NET ([c]) if no specific value for GEP-NECs is available; [b] value includes GEP-NEC and other NEC of orginal cohort; [c] value includes GEP-NECs and NETs of orginal cohort; [d] part of inclusion criteria; [e] only information for 16/18; [f] 21 MANEC included; [g] 73% do not have a Ki-67 index; [h] possible duplicates removed; [i] 2 tests for p16.

Table S3. Overview of Imaging.

Author (Year, Country)	Time Period	Sample Size (GEP-NEC [e])	Lymph. Met: Distant Met	SRS (%)	FDG-PET (%)
Cimitan M. [80] (2003, Italy)	NA	9	NA:7	4+/9 (44)	NA
Fjällskog M.L. [69] (2003, Sweden)	NA	5	NA:5	3+/5 (60)	NA
Artale S. [107] (2005, Italy) [a]	2000–2003	8	NA:8	5+/8 (63)	NA
Srirajaskanthan R. [105] (2009, UK)	NA	6	NA:NA	2+/3 (67)	NA
Binderup T. [111] (2010, Denmark)	2007–2008	14 [b]	NA:NA	NA	13+/14 (93)
Gregory D.L. [77] (2010, Australia)	1996–2007	7	NA: 1	NA	7/7 (100)
Oh S. [110] (2011, Korea)	NA	6	NA:6	NA	36/39 lesions (90)
Sclafani F. [74] (2011, Italy)	1997–2007	6	NA:NA	4+/6 (67)	NA
Hentic O. [85] (2012, France)	2000–2010	19	NA:18	2+/10, 9 NA	NA
Feng S.T. [82] (2013, China)	2009–2013	31	28:13	NA	NA
Lu Z.H. [87] (2013, China)	2009–2011	18 [c]	NA:15	7+/16 (44)	NA
Sorbye H. [4] (2013, 12 Nordic hospitals)	2000–2009	305	188:301	68+/182 (37)	NA
TOTAL				**92/234 (39)** [d]	

[a] Prospective study, all others are retrospective or not stated; [b] Ki-67 >15%; [c] information for only 16; [d] removal of possible overlapping cohorts; [e] includes poorly differentiated endocrine carcinomas, small cell carcinomas, high grade NENs of the gastroenteropancreatic system; GEP-NEC: Gastroenteropancreatic neuroendocrine carcinoma (includes WHO 2010 definition, WHO 2000/2004 definition; poorly differentiated endocrine carcinomas (PDEC), small cell carcinomas, large cell carcinomas and anaplastic/high grade carcinomas); NA: Not Available; Met: Metastases.

Table S4. Overview of Studies including Chemotherapy in GEP-NECs.

Study Author (Year, Country)	Time Period	Original Cohort *n* (GEP-NEC *n*/ Other NEC *n*/ NET *n*)	Median Age GEP-NEC (Range) ([a] (Range))	PS Used	PS (*n*) GEP-NEC ([a])	Lymph Met (*n*): Distant Met (*n*) ([a])	Primary Treatment (*n*) ([a])	1st-Line Chemotherapy [f] ([a])	2nd-Line Chemotherapy [f] ([a])	Grade 3/4 Side Effects ([a])	Effect of Primary Treatment ([a])	Median PFS Mo (Range) ([a] (Range))	MOS Mo (Range) ([a] (Range))
Van der Gaast A. [91] (1990, The Netherlands)	NA	11 (**6**/5/0)	NA (55 (26–72) [b])	WHO	NA (0:2, 1:3, 2:6 [b])	NA: 4	4:C, 2: C+R	**5:CDE, 1: EP**	NA	NA	CR: 3, PR: 2, SD: 1	(7–11)	(10–22)
Moertel C.G. [114] (1991, USA)	1987–1990	45 (**17**/1/27)	NA (52 (24–74) [b])	ECOG	NA (0:5, 1:7, 2:5, 3:1 [b])	NA: NA	17:C	**17: EP**	NA	NA (22%: nausea/vomiting, 35%: leucopenia, 15%: thrombopenia, 22%: anemia, 100%: alopecia, 13%: other [b,c])	NA (CR:3, PR:9, SD:6 [b])	NA (11 (2–21) [b])	NA (19 (5–36+ mo) [b])
Mitry E. [88] (1999, France)	1988–1997	53 (**20**/21/12)	NA (53.4 (20–76) [b])	NA	NA	NA: NA (LD:ED: 8:33 [b])	20:C (58% [b]:prior therapy: 13:S, 13:other C, 3:SSA)	**20: EP**	NA	NA (50/53 [b,c]: 20: nausea/vomiting, 21: leukopenia, 30: neutropenia, 6: thrombocytopenia, 6: anaemia)	NA (CR:4, PR:13, SD:14, PD:10 [b])	NA (8.9 (6.7–13.4) [b])	NA (15 mo (11.7–25 mo) [b])

Table S4. *Cont.*

Study Author (Year, Country)	Time Period	Original Cohort *n* (GEP-NEC *n*/ Other NEC *n*/ NET *n*)	Median Age GEP-NEC (Range) ([a] (Range))	PS Used	PS (*n*) GEP-NEC ([a])	Lymph Met (*n*): Distant Met (*n*) ([a])	Primary Treatment (*n*) ([a])	1st-Line Chemotherapy [f] ([a])	2nd-Line Chemotherapy [f] ([a])	Grade 3/4 Side Effects ([a])	Effect of Primary Treatment ([a])	Median PFS Mo (Range) ([a] (Range))	MOS Mo (Range) ([a] (Range))
Fjällskog M.L. [83] (2001, Sweden)	NA	36 (**4**/0/32)	NA (47.5 (15–70) [c])	NA	NA	NA: 4	4:C (83% [c]:prior therapy: 10:S, 8:R, 17: other C, 4: other)	NA (15: streptozotocin + 5-FU, **21: EP[C]**)	NA (47%: EP [c])	NA (23 neutropenia, 36 alopecia [c])	OR: 2, SD: 1, PD: 0, 1 NA	7 (5–9)	NA (38 [c])
Artale S. [107] (2005, Italy)	2000–2003	19 (**8**/0/11)	54 (40–77)	ECOG	0:3, 1:5	NA: 8	8:C	**8: L-leucovorin + 5-FU + CDDP**	NA	12.5%: neutropenia, 25%: alopecia	PR: 3, SD: 2, PD: 3	4 (1–14)	2-year survival: 66%
Hainsworth J.D. [84] (2006, USA)	1999–2005	78 (**15**/63/0)	NA (58 (21–82) [b])	ECOG	NA (0:8, 1:59, 2:11 [b])	NA: 15	15:C	**15: paclitaxel** + **carboplatin** + **VP-16**	NA	NA (Grade 3: 50% neutropenia, 31%: thrombopenia, 15%: anemia, 18% fatigue, 13%: infection, 10% nausea/vomiting, other: 15%. Grade 4: 51% neutropenia, 19% hospitalization (neutropenia/fever), 8% infection, 40% RBC transfusion, other: 17% 4% treatment related death [b])	NA (CR: 12, PR: 29, SD: 23, PD: 7, NA: 7 [b])	NA (7.5 [c])	NA (14.5 [c])

Table S4. *Cont.*

Study Author (Year, Country)	Time Period	Original Cohort *n* (GEP-NEC *n*/ Other NEC *n*/ NET *n*)	Median Age GEP-NEC (Range) ([a] (Range))	PS Used	PS (*n*) GEP-NEC ([a])	Lymph Met (*n*): Distant Met (*n*) ([a])	Primary Treatment (*n*) ([a])	1st-Line Chemotherapy [f] ([a])	2nd-Line Chemotherapy [f] ([a])	Grade 3/4 Side Effects ([a])	Effect of Primary Treatment ([a])	Median PFS Mo (Range) ([a] (Range))	MOS Mo (Range) ([a] (Range))
Bajetta E. [120] (2007, Italy)	2000–2005	40 (**5**/7/27)	NA (59 (46–70) [b])	ECOG	NA (0:8, 1:3, 2:2 [b])	NA: 5	5:C	**5: XELOX**	NA	NA	NA (PR: 3, SD: 1, PD: 9 [b])	NA (4 (1–43 [c])	NA (5 (1–44+ [c])
Correale P. [59] (2007, Italy)	1999–2003	27 (**10**/17/0)	NA (63.5 (47–78) [b])	ECOG	NA(≤2 [b])	NA: 10	10:C (48% [b]:prior therapy: S, C)	4–10: **EP + lanreotide longlasting** (6/27[C] received 5-FU as 1st-line C)	0–6: **EP + lanreotide longlasting** (6/27[C] received 5-FU as 1st-line C)	NA (Grade 3: 15 trombocytopenia, 14 anaemia, 25 neutropenia, 16 fever, 10: other [c])	NA (CR: 4, PR: 6, SD: 12, PD: 5 [b])	NA (9 [b])	NA (relative survival: 24 [b])
Turner N.C. [119] (2010, UK)	1999–2008	82 (**5**/10/67)	NA (55 (22–82) [b,c])	NA	NA (0–1:71, 2:11 [b,c])	NA: NA (NA:71 [b,c])	5:C (30% [b,c]:prior therapy: SSA, INF-alfa, R, other)	5: **5-FU + CDDP + streptozocin**	NA	NA (grade 3: 22 nausea/vomitting, 6 fattigue, 6 diarrhea, 21 neutropenia, 6 haemoglobin, 3 platelet, 8: other. Grade 4: 4 nausea/ vomitting, 9: other [b,c])	NA (PR: 9, SD: 3, PD: 3 [b])	NA (9.1 [b,c])	NA (31.5 [b,c])

Table S4. *Cont.*

Study Author (Year, Country)	Time Period	Original Cohort *n* (GEP-NEC *n*/ Other NEC *n*/ NET *n*)	Median Age GEP-NEC (Range) ([a] (Range))	PS Used	PS (*n*) GEP-NEC ([a])	Lymph Met (*n*): Distant Met (*n*) ([a])	Primary Treatment (*n*) ([a])	1st-Line Chemotherapy [f] ([a])	2nd-Line Chemotherapy [f] ([a])	Grade 3/4 Side Effects ([a])	Effect of Primary Treatment ([a])	Median PFS Mo (Range) ([a] (Range))	MOS Mo (Range) ([a] (Range))
Deutschbein T. [116] (2011, Germany)	2005–2009	18 (**12**/1/5)	(39–75)	KPS	NA (<60% [b,c])	7:11	12:C (50% [b,c]: prior therapy: S)	**5: carboplatin + paclitaxel + VP-16, 9: EP** [e]	NA	NA (carboplatin + paclitaxel + VP-16: grade 3: 25% diarrhea/ nausea, 13% infection, 13% sensory neuropathy, 50% thrombocytopenia, grade 4: 12% motor neuropathy, 13% thrombocytopenia. EP: grade 4: 17% infection without neutropenia, 17% thrombocytopenia [b,c])	Regime A: PR: 1, SD: 1, PD: 1, 2 NA Regime B: PR: 1, SD: 3, PD: 5	Regime A: (3.2–10.0), Regime B: (2.8–26.4)	NA
Hentic O. [100] (2011, France)	1997–2006	63 (**18**/0/45)	58 (33–71)	NA	NA	NA: 18 [d]	14:C, 3:S+C, 1:C+ liver S	**17: EP**, 1: other C	NA	NA	EP: PR: 5, SD: 4, PD: 8	NA	14
Jin S. [86] (2011, China)	2004–2008	15 (**9**/6/0)	NA (49 (31–74) [b])	ECOG	NA (0:4, 1:10, 2:1 [b])	NA: 9	9:C (other therapy NA)	**9: IP**	NA	NA (10: neutropenia, diarrhea, febrile neutropenia [c])	NA (CR: 3, PR: 7, SD: 3, PD: 2 [b])	NA (4.5 [b])	NA (11.4 [b])
Welin S. [108] (2011, Sweden, Norway)	2004–2009	25 (**17**/5/0)	NA (55 (32–76) [b])	NA	NA (0–2 [b])	NA: 17	17:C (NA if other therapy)	16-17: EP (1/24[C]: doxorubicin + docetaxel)	**17 temozolomide** as 2nd/3rd-line (18/25 2nd-line, 7/25 3rd-line [c])	NA (all treated with temozolomide: grade 3: 1 leukopenia + thrombopenia [b])	NA (temozolomide: CR: 1, PR: 7, SD: 9)	NA (6 [b])	NA (22, from diagnosis: 32 [b])

Table S4. *Cont.*

**Study Author (Year, Country)**	**Time Period**	**Original Cohort *n* (GEP-NEC *n*/ Other NEC *n*/ NET *n*)**	**Median Age GEP-NEC (Range) ([a] (Range))**	**PS Used**	**PS (*n*) GEP-NEC ([a])**	**Lymph Met (*n*): Distant Met (*n*) ([a])**	**Primary Treatment (*n*) ([a])**	**1st-Line Chemotherapy [f] ([a])**	**2nd-Line Chemotherapy [f] ([a])**	**Grade 3/4 Side Effects ([a])**	**Effect of Primary Treatment ([a])**	**Median PFS Mo (Range) ([a] (Range))**	**MOS Mo (Range) ([a] (Range))**
Hentic O. [85] (2012, France)	2000–2010	19 (**19**/0/0)	53 (29–78)	ECOG	0–1:14, 2:5	NA: 18	19:C	19: EP	**19: FOLFIRI**	3: neutropenia, 3: diarrhea (2nd-line FOLFIRI)	OR: 6, SD: 6, PD: 7 (2nd-line FOLFIRI)	4	18
Nakano K. [118] (2012, Japan)	2005–2011	44 (**9**/35/0)	NA (60 (20–80) [b])	NA	NA (0:24, 1:11, ≥2:2 [b])	NA: NA (LD:ED: 14:30 [b])	9:C (57% [b]:prior therapy: other C, S, R)	**0–9: IP** (9/44[C]: prior C)	**0-9: IP** (9/44[C]: prior C)	NA (grades 3/4: 19 leukopenia, 26 neutropenia, 12 thrombopenia, 7: other [b])	CR: 1, PR: 3 (OR: 22, CR: 3, PR: 19 [b])	5.1	NA (1 year: 67% [b])
Olsen I.H. [117] (2012, Denmark)	2007–2009	31 (**15**/16/0)	NA (63 (33–82) [b])	WHO	NA (0:10, 1:16, 2:5 [b])	NA: 15	15:C	**15: carboplatin + VP-16 + vincristine**	NA	NA (Grade 4: 8 neutropenia, Grade 3: 7 neutropenia, 3 thrombopenia [b])	NA (CR: 3, PR: 13, SD: 8, PD: 7 [b])	NA (6.6 [b])	NA (15.3 [b])
Olsen I.H. [122] (2012, Denmark)	NA	28 (**18**/10/0)	NA (58 (32–81) [b])	WHO	NA (0–1:22, 2:6 [b])	NA: 18	18:C	18: carboplatin + VP-16	**18: temozolomide as 2nd- or 3rd-line** (19/28: 2nd-line, 9/28: 3rd-line)	NA (Grade 3: 1 leucopenia, Grade 4: 2 thrombocytopenia [b])	All progressed on carboplatin + VP-16 [d]), temozolomide: NA (CR: 0, PR: 0, SD: 6, PD: 10 [b])	NA (2.4 [b])	NA (3.5 [b])

Table S4. *Cont.*

Study Author (Year, Country)	Time Period	Original Cohort *n* (GEP-NEC *n*/ Other NEC *n*/ NET *n*)	Median Age GEP-NEC (Range) ([a] (Range))	PS Used	PS (*n*) GEP-NEC ([a])	Lymph Met (*n*): Distant Met (*n*) ([a])	Primary Treatment (*n*) ([a])	1st-Line Chemotherapy [f] ([a])	2nd-Line Chemotherapy [f] ([a])	Grade 3/4 Side Effects ([a])	Effect of Primary Treatment ([a])	Median PFS Mo (Range) ([a] (Range))	MOS Mo (Range) ([a] (Range))
Terashima T. [115] (2012, Japan)	2000–2008	41 (**34**/7/0)	46.5–63.5 (27–79)	ECOG	0:8, 1:25, 2:1	18: 34 [d]	34:C (NA if other treatment)	**17: EP, 17: IP**	NA	NA	CR: 0, PR: 6, SD: 6, PD: 4, NA: 2	NA	7.8–14.9
Ahn H.K. [123] (2013, Korea)	2010–2012	37 (**13**/0/24)	NA (55 (19–71) [c])	ECOG	NA (0:4, 1:33 [c])	NA: NA (NA:36 [c])	13:C (51% [c]:prior therapy: S, R, C)	13: NA regime	**13: pazopanib**	NA	23% (CR+PR)	5.8	NA
Du Z. [81] (2013, China)	2008–2010	11 (**11**/0/0)	53 (35–74)	ECOG	0:3, 1:7, 2:1	5: 7	7:C, 4:S+C	**11: FOLFIRI**	2: EP, 1: CAV	3 neutropenia, 1 thrombopenia, 2 diarrhea, 3 nausea/vomiting	CR: 0, PR: 7, SD: 3, PD: 1	6.5 (2.5–17+)	13 (6–40)
Ferrarotto R. [121] (2013, Brazil)	2003–2011	24 (**3–9**/ 3–9/15)	NA (56 (23–73) [b,c])	ECOG	NA (0:5, 1:12, ≥2:7 [b,c])	NA: 3–9	3–9:C (50% [b,c]:prior therapy: S, C)	**0–9: XELOX** (12/24:XELOX as 2nd/3rd line)	**0–9: XELOX** (12/24: XELOX as 2nd/3rd line)	NA (grade 3: 2 neuropathy, 2 diarrhea, 2 other [b,c])	NA (PR: 7, SD: 17 [b,c])	NA (9.8 [b,c])	NA
Lu Z.H. [87] (2013, China)	2009–2011	18 (**18**/0/0)	60 (31–66)	KPS	<90: 44%, ≥90: 56%	NA: 15	16:C	**15: IP, 1: EP**	1: IP, others NA	5 leukocytopenia, 9 neutropenia, 2 diarrhea, 1 nausea, 1 vomitting	CR: 1, PR: 7, SD: 3, PD: 3, 2 NA	5.5	10.6

Table S4. *Cont.*

Study Author (Year, Country)	Time Period	Original Cohort *n* (GEP-NEC *n*/ Other NEC *n*/ NET *n*)	Median Age GEP-NEC (Range) ([a] (Range))	PS Used	PS (*n*) GEP-NEC ([a])	Lymph Met (*n*): Distant Met (*n*) ([a])	Primary Treatment (*n*) ([a])	1st-Line Chemotherapy [f] ([a])	2nd-Line Chemotherapy [f] ([a])	Grade 3/4 Side Effects ([a])	Effect of Primary Treatment ([a])	Median PFS Mo (Range) ([a] (Range))	MOS Mo (Range) ([a] (Range))
Sorbye H. [4] (2013, 12 Nordic hospitals)	71	305 (**305**/0/0)	60 (24–89)	WHO	0:83, 1:133, 2:49, 3–4:27	188: 301	252:pallative C, 53:BSC (83:S + C/BSC)	**129: EP, 67: carboplatin + VP-16, 28: carcboplatin + VP-16 + vincristine, 28: others**	**35: temozolomide, 20: taxotere-based, 44: others**, (31: 3rd-line)	NA	1st-line EP: CR: 2%, PR: 29%, SD: 37%, PD: 32%, 1st-line carboplatin + VP-16: CR: 2%, PR: 28%, SD: 32%, PD: 38%, 1st -line carcboplatin + VP-16 + vincristine: CR: 0%, PR: 44%, SD: 24%, PD: 32%). 2nd-line C: RR: 18%, 3rd-line C: RR: 7%	EP: 4 (95% CI: 3.1–4.6), carboplatin + VP-16: 4 (95% CI: 2.6–5.4), carcboplatin + VP-16 + vincristine: 4 (95% CI: 3.3–4.7)	Without C: 1, with C: 11

Table S4. *Cont.*

**Study Author (Year, Country)**	**Time Period**	**Original Cohort *n* (GEP-NEC *n*/ Other NEC *n*/ NET n)**	**Median Age GEP-NEC (Range) ([a] (Range))**	**PS Used**	**PS (*n*) GEP-NEC ([a])**	**Lymph Met (*n*): Distant Met (*n*) ([a])**	**Primary Treatment (*n*) ([a])**	**1st-Line Chemotherapy [f] ([a])**	**2nd-Line Chemotherapy [f] ([a])**	**Grade 3/4 Side Effects ([a])**	**Effect of Primary Treatment ([a])**	**Median PFS Mo (Range) ([a] (Range))**	**MOS Mo (Range) ([a] (Range))**
Vélayoudom -Céphise F.L. [90] (2013, France)	2000–2005	28 (**10**/18/0)	NA (55 (29–79) [b])	NA	NA	NA: NA (NA:86% [b])	10C (100% [b]: prior therapy: S, SSA, R, other)	**0–10: cisplatin-based** (14/28: prior C)	**0–10: cisplatin-based** (14/28: cisplatin-based 2nd-line)	NA	NA (CR/PR: 5, SD: 6, PD: 9 [b])	NA	NA (poorly differentiated: 17 (1–113), well-differentiated: 41 (7–143) [b])
Olsen I.H. [124] (2014, Denmark)	NA (3-year period)	22 (13/9/0)	NA (65 (35–77) [b])	WHO	NA (0:6, 1:10, 2:3, 3:2, 4:1 [b])	NA: 13	13:C (NA if other treatment)	NA (all exposed to carboplatin + VP-16 [c])	**13: topotecan** as 2nd/3rd/4th-line	NA (grade 3: 3 leukopenia, 3 thrombocytopenia, grade 4: 2 thrombocytopenia [b])	NA (topotecan: CR: 0, PR: 0, SD: 5, PD: 17 [b])	NA (2.1 [b])	NA (3.2 (from topotecan study inclusion) [b])

Table S4. *Cont.*

Study Author (Year, Country)	Time Period	Original Cohort *n* (GEP-NEC *n*/ Other NEC *n*/ NET *n*)	Median Age GEP-NEC (Range) ([a] (Range))	PS Used	PS (*n*) GEP-NEC ([a])	Lymph Met (*n*): Distant Met (*n*) ([a])	Primary Treatment (*n*) ([a])	1st-Line Chemotherapy [f] ([a])	2nd-Line Chemotherapy [f] ([a])	Grade 3/4 Side Effects ([a])	Effect of Primary Treatment ([a])	Median PFS Mo (Range) ([a] (Range))	MOS Mo (Range) ([a] (Range))
Yamaguchi T. [92] (2014, Japan)	2000–2011	258 (**258**/0/0)	62.5 (26–81)	ECOG	0–1:240, ≥2:18	131: 219	76:S+C, 182:C	**160: IP, 46: EP, 37: fluoro-pyrimidine-based, 18: other**	**25: amrubicin, 23: VP-16, cisplatin/ carboplatin, 21: irinotecan, 11: S-1, 5: IP**	NA	IP: RR: 50%, EP: RR 28%. 2nd-line: amrubicin: RR 4%, VP-16, cisplatin/ carboplatin: RR 17%, irinotecan: RR: 5%, S-1: RR: 27%, IP: RR: 40%	IP: 5.2, EP: 4	11.5 mo, IP: 13, EP: 7.3 (OBS different distribution of primary site)

GEP-NEC: Gastroenteropancreatic neuroendocrine carcinoma (includes WHO 2010 definition, WHO 2000/2004 definition; poorly differentiated endocrine carcinomas (PDEC), small cell carcinomas, large cell carcinomas and anaplastic/high grade carcinomas); Other NEC: neuroendocrine carcinoma/poorly differentiated carcinoma/small cell carcinoma of sites other than the gastroenteropancreatic system; NET: neuroendocrine tumour/well differentiated endocrine tumour/well differentiated carcinoma/low grade tumour; NA: Not Available; PS: Performance Score; ECOG: Eastern Cooperative Oncology Group; KPS: Karnofsky performance status score; LD (Limited Disease): confined to a localized anatomic region/organ of origin with locoregional lymph node involvement, (encompassable within one (tolerable) radiation therapy treatment port/radiation field), ED (Extensive Disease): beyond LD; Met: Metastases; S: Surgery; C: Chemotherapy; R: Radiation; SSA: Somatostatin Analogues; BSC: Best Supportive Care; CDE: cyclophosphamide, doxorubicin, etoposide; EP: cisplatin + etoposide; 5-FU: 5-fluorouracil; CDDP: cisplatin; IP: irinotecan + cisplatin; FOLFIRI: irinotecan, leuocovorin, 5-fluorouracil; CAV: cyclophosphamide + doxorubicin + vincristine; CR: Complete Response; PR: Partial Response; SD: Stable Disease; PD: Disease Progression; OR: Overall response; RR: Response Rate (CR+PR); PFS: Progression Free Survival; MOS: Median Overall Survival; Mo: months; [a] includes GEP-NEC + other NEC([b]) and/or NET([c]) if no specific value for GEP-NECs is available; [b] value includes GEP-NEC and other NEC of orginal cohort; [c] value includes GEP-NECs and NETs of orginal cohort; [d] part of inclusion criteria; [e] 2 patients received both treatments; [f] high-lighted: study aim/focus.

Table S5. Overview of Studies Including Oesophageal Neuroendocrine Carcinomas.

Author (Year, Country)	Time Period	% of Oesophageal Carcinomas that Are NECs	*N* Oesophageal NECs (Me:W)	Predisposing Factors	Age Year (Range)	Size cm (Range)	Location of Primary Tumour (U:M:L)	Symptoms (*n* Dysphagia/ Weight Loss/ Pain/Other)	Symptom Duration Mo (Range)	*n* LD: *n* ED	*n* Lymph. Met.	*n* Distant Met. at Presentation/ Study Start (Sites)	*n* SC: *n* LC	*n* + Other Histological Components	Overall Survival Mo (Range)	*n* Survivors >2 Years: Time to Death or End of Follow-Up
Reyes CV [131] (1980, USA)	1947–1978	1.7	16 (16:0)	NA	M^1: 58 (29–74)	Average: 5.39 (1–10)	2:10:4	11/12/4/ 6 anorexia	M^1: 3 (1–12)	NA	NA	NA	16:0	4 + squamous component, 2 + mucosubstance	Average: 2 (1–6)	0
Briggs JC [134] (1983, U.K.)	1969–1983	2.4	23 (8:15)	NA	M^1:69.8 (51–88)	(2.5–14/ ”large”)	0:5:18	22/1/1/0	(2 weeks–12 mo)	NA	11	4 (3 liver, 1 lung) [w]	23:0 (16 mix)	2 + squamous differentiation, 1 + glandular differentiation	(8 days–14 mo)	0
Doherty MA [138] (1983, U.K.)	NA	NA	6 (2:4)	NA	(69–83)	(5–8)	0:4:2	4/4/0/ 1 regurgitation	NA	NA	NA	1 (liver)	16 [y]:0	NA	M^2: 3 (NA)	0
Mori M [153] (1989, Japan)	1972–1986	NA	10 (8:2)	NA	M^1: 65 (55–76)	M^1: 5.4 (2–13)	1:8:1	6/0/4/ 1 tonic-clonic seizure	(1–8)	NA	NA	≥4 (1 brain), NA others	10 [y]:0	2 + squamous and glandular differentiation, 5 + squamous differentiation, 1 NA	(1–33)	1: 33 mo
Tennvall J [162] (1990, Sweden)	1970–1987	1.25	7 (5:2)	NA	M^2: 71 (40–89)	(3.5–16)	1:4:2	5/3/3/0	M^2: 3 (0–12)	NA	NA	3 (2 lung, 1 bone)	7 [y]:0	2 + squamous carcinoma	(1.5–23)	0

Table S5. *Cont.*

Author (Year, Country)	Time Period	% of Oesophageal Carcinomas that Are NECs	*N* Oesophageal NECs (Me:W)	Predisposing Factors	Age Year (Range)	Size cm (Range)	Location of Primary Tumour (U:M:L)	Symptoms (*n* Dysphagia/ Weight Loss/ Pain/Other)	Symptom Duration Mo (Range)	*n* LD: *n* ED	*n* Lymph. Met.	*n* Distant Met. at Presentation/ Study Start (Sites)	*n* SC: *n* LC	*n* + Other Histological Components	Overall Survival Mo (Range)	*n* Survivors >2 Years: Time to Death or End of Follow-Up
Beyer KL [133] (1991, USA)	1969–1989	4.7	11 (8:3)	NA	M[1]: 64 (53–88)	M[1]: 7.9 (3–14)	2:6:3	8/0/1/ 2 odynophagia, 1 hematemesis	M[1]: 3.9 (1.5–8)	NA	5	9 (4 liver, 3 bone, 1 diaphragma, 1 lung) [w]	11 [y]:0	1 + squamous differentiation, 1 + glandular differentiation	Average: 5.4 (10 days–16 mo)	0
Craig SR [136] (1995, U.K.)	10 years period	2.7 [a]	16 (5:11)	NA	M[1]: 60 (39–72)	NA	NA	16/16/0/ 2 hoarseness, 1 hematemesis + melena	NA	NA	7	9 (8 distant lymph nodes, 1 liver)	16 [y]:0	5 + squamous carcinoma, 1 + adenocarcinoma	(14 days–96 mo)	1: 96 mo (S+C)
Huncharek M [141] (1995, USA)	1978–1993	1.6	13 (11:2)	10: smokers, mean of 44 pack-years (10–80) [f], 2: heavy alcohol use [f]	M[1]: 67 (51–78)	NA	3:7:3	12/0/2/0	NA	8:5	NA	NA	13 [y]:0	NA	M[2]: 7 (3–17)	1: 24 mo (C+R)
Galanis E. [9] (1997, USA)	1974–1994	NA	8 (NA)	NA	NA	NA	NA	NA	NA	6:2	NA	NA	8 [y]:0	NA	NA	NA
Nishimaki T [156] (1997, Japan)	1974–1994	1.6	13 (12:1)	NA	Average: 63.5 (50–78)	M[1]: 7.4 (2.3–14.0)	0:10:3	NA	NA	NA	12	6 (2 liver, 5 distant lymph nodes)	13 [y]:0	NA	M[2]: 10 (1–106)	2: 64, 106 mo (S+C)

Table S5. *Cont.*

**Author (Year, Country)**	**Time Period**	**% of Oesophageal Carcinomas that Are NECs**	***N* Oesophageal NECs (Me:W)**	**Predisposing Factors**	**Age Year (Range)**	**Size cm (Range)**	**Location of Primary Tumour (U:M:L)**	**Symptoms (*n* Dysphagia/ Weight Loss/ Pain/Other)**	**Symptom Duration Mo (Range)**	***n* LD: *n* ED**	***n* Lymph. Met.**	***n* Distant Met. at Presentation/ Study Start (Sites)**	***n* SC: *n* LC**	***n* + Other Histological Components**	**Overall Survival Mo (Range)**	***n* Survivors >2 Years: Time to Death or End of Follow-Up**
Poynton AR [160] (1997, Ireland)	1977–1994	1.1	11 (4:7)	NA	M^2: 71 (39–84)	NA	0:2:9	11/5/6/0	(1–6)	NA	NA	3 (NA)	11 [y]:0	NA	(22 days–54 mo)	0
Takubo K [31] (1999, Japan)	NA	NA	21 (15:6)	NA	(48–89)	NA	NA	NA	NA	NA	16 (3 NA)	NA	20 [y]:0	7 + *in situ* squamous cell carcinoma, 4 + squamous cell carcinoma, 1 + mucoepidermoid carcinoma	M^2: 11 (4–26)	2: 24, 26 mo (C+R, C+S)
Bennouna J [132] (2000, France)	1993–1998	2.8	10 (7:3)	4: smokers [f] + heavy drinkers [g], 4: smokers	M^2: 62 (48–73)	NA	0:3:7	10/3/0/ 1 dysphonia, 1 hiccup	(1–3)	4 [p]:6	NA	6 (3 liver, 1 bone, 2 distal lymph nodes)	10 [y]:0	NA	M^2: 15.5 (2–36)	1: 36 mo
Lam KY [146] (2000, Hong Kong)	1982–1996	1.3	20 (14:6)	14: smokers [h] + drinkers [i], 2: smokers, 1: drinker	M^2: 60 (40–84)	M^2: 7.5 (3.5–15)	1:8:11	16/0/2/ 1 shortness of breath, 1 hematemesis	M^2: 1 (1–40 weeks)	NA	17	3 (sites NA)	20 [y]:0	NA	M^2: 3.4 (13 days–6 years)	1: 6 years (S+C+R)
Medgyesy DC [150] (2000, USA)	1944–1997	NA	8 (6:2)	7: smokers [f], 3: heavy drinkers [f]	M^2: 58 (31–79)	NA	1:2:5	7/5/2/ 2 hematemesis, 1 SIADH	NA	6:2	NA	2 (sites NA)	8 [y]:0	0	M^2: 12.5 (5–57)	2: 33, 57 mo (R, S+C+R)

Table S5. *Cont.*

Author (Year, Country)	Time Period	% of Oesophageal Carcinomas that Are NECs	*N* Oesophageal NECs (Me:W)	Predisposing Factors	Age Year (Range)	Size cm (Range)	Location of Primary Tumour (U:M:L)	Symptoms (*n* Dysphagia/ Weight Loss/ Pain/Other)	Symptom Duration Mo (Range)	*n* LD: *n* ED	*n* Lymph. Met.	*n* Distant Met. at Presentation/ Study Start (Sites)	*n* SC: *n* LC	*n* + Other Histological Components	Overall Survival Mo (Range)	*n* Survivors >2 Years: Time to Death or End of Follow-Up
Mitani M [152] (2000, Japan)	1982–1999	2.8	5 (5:0)	NA	(39–70)	NA	2:3:0	NA	NA	NA	3	NA (none mentioned)	5 [y]:0	4 + squamous cell carcinoma	(11 mo–9 years)	2: 7, 9 years (S, S+C+R)
Cheuk W. [35] (2001, Hong Kong, USA)	NA	NA	7 (NA)	NA	NA	NA	NA	NA	NA	NA	NA	NA	7 [y]:0	1 + squamous cell carcinoma	NA	NA
Chow V [180] (2001, Hong Kong)[ø]	1991–2000	NA	5 (4:1)	4: smokers [f], 4: drinkers [f]	M^1: 58.2 (40–70)	(2–12)	2:1:2	NA	NA	NA	4	0	5 [y]:0	NA	(3.5–50)	1: 50 mo (S)
Nemoto K [155] (2002, Japan)	1985–1999	1.8	24 [d] (18:2)	NA	M^2: 65 (49–76)	NA	NA	NA	NA	20 [q]:0	NA	0	20 [y]:0	NA	M^2: 11.7	3: 56, 99, 108 mo, (all S+C+R)
Osugi H [158] (2002, Japan)	1986–2000	2.0	9 (8:1)	NA	M^2: 65 (49–71)	NA	1:3:5	8/0/0/0	NA	NA	8 (1 NA)	NA [w]	9 [y]:0	NA	M^2: 11.0 (4–76 mo)	1: 76 mo (S+C)
Pantvaidya GH [159] (2002, India)	1985–2001	0.15	24 [d] (13:5)	NA	M^1: 62 (48–80)	NA	1:14:3	16/0/1/ 1 hoarseness	NA	10:8	NA	7 (5 liver, 1 coeliac nodes)	18 [y]:0	2 + epidermoid differentiation, 1 + squamous differentiation	M^2: 6 (1–48)	1: 48 mo (S+C+R)

Table S5. *Cont.*

**Author (Year, Country)**	**Time Period**	**% of Oesophageal Carcinomas that Are NECs**	***N* Oesophageal NECs (Me:W)**	**Predisposing Factors**	**Age Year (Range)**	**Size cm (Range)**	**Location of Primary Tumour (U:M:L)**	**Symptoms (*n* Dysphagia/ Weight Loss/ Pain/Other)**	**Symptom Duration Mo (Range)**	***n* LD: *n* ED**	***n* Lymph. Met.**	***n* Distant Met. at Presentation/ Study Start (Sites)**	***n* SC: *n* LC**	***n* + Other Histological Components**	**Overall Survival Mo (Range)**	***n* Survivors >2 Years: Time to Death or End of Follow-Up**
Noguchi T [157] (2003, Japan)	NA	1.4	6 (6:0)	NA	M[1]: 70 (55–78)	NA	0:5:1	NA	NA	NA	5	3 (distant lymph nodes)	6 [y]:0	NA	M[1]: 9.3 (2–24)	1: 24 mo (S)
Brenner B. [7] (2004, USA)	1980–2002	NA	19 (NA)	8/18 (smokers or drinkers or reflux) [f]	NA	NA	NA	NA	NA	NA	NA	NA	19 [y]:0	NA	NA	NA
La Rosa S. [58] (2004, Italy)	NA	NA	8 (7:1)	NA	M[1]: 60 (45–73)	M[1]: 3.6 (2.3–5.5)	NA	NA	NA	NA	NA	NA	NA	NA	NA	NA
Wu Z [165] (2004, China)	1985–2000	1.0	9 (6:3)	NA	M[1]: 56 (45–66)	Average: 5 (3–7)	1:5:3	6/6/6/0	NA	NA	7	0	6 [y]:0	3 + squamous carcinoma *in situ*	(5–37) 3 NA	1: 37 mo (S+C+R)
Hosokawa A [170] (2005, Japan)	1990–2001	0.8	14 (14:0)	13: smokers [f] + drinkers [f], 1: drinker	M[2]: 63 (37–71)	11: >5 cm, 3: <5 cm	1:7:6	13/0/1/0	(1–5)	3:11	NA	11 (8 cervical lymph/node, 1 para-aortic lymph node, 3 liver, 1 lung, 1 bone)	14 [y]:0	NA	M[2]: 7.7 (0.6–89.1)	3: 24, 28.2, 89.1 mo (C, C+R, S+R)
Kim K.O. [13] (2006, Korea)	1998–2005	NA	6 (NA)	NA	NA	NA	NA	NA	NA	NA	NA	NA	6 [y]:0	NA	NA	NA

Table S5. *Cont.*

Author (Year, Country)	Time Period	% of Oesophageal Carcinomas that Are NECs	*N* Oesophageal NECs (Me:W)	Predisposing Factors	Age Year (Range)	Size cm (Range)	Location of Primary Tumour (U:M:L)	Symptoms (*n* Dysphagia/ Weight Loss/ Pain/Other)	Symptom Duration Mo (Range)	*n* LD: *n* ED	*n* Lymph. Met.	*n* Distant Met. at Presentation/ Study Start (Sites)	*n* SC: *n* LC	*n* + Other Histological Components	Overall Survival Mo (Range)	*n* Survivors >2 Years: Time to Death or End of Follow-Up
Li A.F.Y. [42] (2006, Taiwan)	1985–2004	NA	6 (6:0)	NA	(45–82)	NA	NA	NA	NA	NA	5	0	NA	NA	(1–37)	2: 32, 37 mo
Uccella S. [37] (2006, Italy)	1982–2004	NA	6 (4:2)	NA	(49–73)	(2–5.5) 1 NA	NA	NA	NA	NA	NA	(6 ”malignant”)	NA	NA	NA	NA
Hudson E [174] (2007, U.K	1998–2005	NA	16 (4:12)	NA	M[2]: 68.5 (48–81)	NA	0:3:13	NA	NA	6:9 1 NA	NA	9 (7 liver, 1 lungs, 2 abdominal lymph nodes, 1 spleen, 1 supraclavicular fossa, 1 pancreas)	16 [y]:0	NA	M[2]: 13.2 (0.4–104)	3: 30, 48 and 104 mo (all C+R)
Koide N [142] (2007, Japan)	1991–2005	3.1	10 (9:1)	NA	(51–75)	(3–9.5)	1:7:2	NA	NA	NA	10	0	10 [y]:0	NA	M[2]: 13.0, M[1]: 22.7 (7–80)	2: 37, 80 mo (C+R, S+C)
Ku GY [143] (2007, USA)	1980–2005	1.0	25 [d] (18:4)	7: alcohol [f] and tobacco use [f], 2: tobacco use, 1: alcohol use	M[2]: 60 (51–73)	NA	2:1:19	16/9/11/ 2 bleeding	NA	14:8	NA	8 (5 liver, 4 distant lymph nodes, 1 lung, 1 adrenal, 1 bone, 1 bone marrow)	22 [y]:0	2 + adenocarcinoma, 1 + squamous cell, 1 + sarcomatoid	M[2]: 19.8 (1.5–11.2 years)	6: 2.2, 2.6, 2.6, 3.6, 5.8 and 11.2 (all C, then 5: C+R, 1: S +C+R)

Table S5. *Cont.*

Author (Year, Country)	Time Period	% of Oesophageal Carcinomas that Are NECs	*N* Oesophageal NECs (Me:W)	Predisposing Factors	Age Year (Range)	Size cm (Range)	Location of Primary Tumour (U:M:L)	Symptoms (*n* Dysphagia/ Weight Loss/ Pain/Other)	Symptom Duration Mo (Range)	*n* LD: *n* ED	*n* Lymph. Met.	n Distant Met. at Presentation/ Study Start (Sites)	*n* SC: *n* LC	*n* + Other Histological Components	Overall Survival Mo (Range)	*n* Survivors >2 Years: Time to Death or End of Follow-Up
Lee S.S. [15] (2007, Korea)	1995–2004	NA	7 (NA)	NA	NA	NA	NA	NA	NA	NA	NA	NA	7 [y]:0	NA	NA	NA
Lepage C. [1] (2007, U.K.)	1986–1999	NA	557 (NA)	NA	NA	NA	NA	NA	NA	NA	NA	NA	557 [y]:0	NA	Relative 1 year survival: 18.0%	NA
Yau KK [167] (2007, Hong Kong)	1993–2003	2.2	10 (6:4)	10: smokers [f] + drinkers [f]	M^2: 69 (55–86)	(2–10)	0:7:3	10/0/0/0	(1–12 weeks)	4:6 [r]	NA	6 (3 distant lymph., 2 liver, 1 brain)	10 [y]:0	NA	M^2: 8 (2–62)	2: 34, 62 mo (C+R)
Yun JP [178] (2007, China)	1989–2005	0.5	21 (16:5)	NA	M^2: 56 (30–76)	M^1: 5.8 (1.2–15.5)	1:12:8	18/0/3/0	M^1: 2 (10 days–9 mo)	NA	10	3 (2 liver, 1 thyroid)	21 [y]:0	2 + well differentiated squamous carcinoma	M^2: 18.3 (3–71)	1: 46 mo (C+S), 5: 28–71 mo (2: S+C+R, 1: S+C, 1: S+R, 1: R)
Chin K [169] (2008, Japan)	1999–2004	2.4	15 [d] (11:1)	NA	M^2: 66 (53–77)	M^2: 6 (3–13)	1:7:3 (1 NA)	NA	NA	NA	NA	7 (NA)	12 [y]:0	4 + squamous cell	M^2: 417 days (97–1626)	3: 1465, 1291, 1626 days (all C+R)
Lv J [148] (2008, China)	1985–2005	1.2	126 (99:27)	NA	M^2: 58	M^2: 5.5	22:78:26	NA	NA	85:41	92	41 (30 distant lymph, others NA)	126 [y]: 0	NA	M^2: 12.5 (95% CI: 10.3–14.7)	12: >5 years (3: S, 1: R, 6: S+C, 2: R+C

Table S5. *Cont.*

Author (Year, Country)	Time Period	% of Oesophageal Carcinomas that Are NECs	*N* Oesophageal NECs (Me:W)	Predisposing Factors	Age Year (Range)	Size cm (Range)	Location of Primary Tumour (U:M:L)	Symptoms (*n* Dysphagia/ Weight Loss/ Pain/Other)	Symptom Duration Mo (Range)	*n* LD: *n* ED	*n* Lymph. Met.	*n* Distant Met. at Presentation/ Study Start (Sites)	*n* SC: *n* LC	*n* + Other Histological Components	Overall Survival Mo (Range)	*n* Survivors >2 Years: Time to Death or End of Follow-Up
Maru DM [149] (2008, USA)	1997–2007	NA	42 [d] (35:5)	NA	Average: 63 (34–82)	NA	1:5:33 (1 NA)	18/0/0/ 10 reflux, 6 symptoms from metastases, 4 hematemesis/ blood in stool	NA	21 LD: 17 ED [s] (2 NA)	NA	17 (8 liver, 4 abdominal lymph nodes, 2 bone, 3 lung, 1 brain)	13:27	1 + squamous (SC NEC), 15 + adenocarcinoma (3 SC, 12 LC NEC)	M^2: 14 (2–49)	NA
Shia, J. [29] (2008, USA)	NA	NA	6 (NA)	NA	NA	NA	NA	NA	NA	NA	NA	NA	5:0 (1 mixed)	NA	NA	NA
Lepage C. [79] (2009, EUROCARE database)	1985–1994	NA	419 (NA)	NA	NA	NA	NA	NA	NA	NA	NA	NA	419 [y]:0	NA	5-year relative survival rate: 5.3	NA
Sadanaga N [171] (2009, Japan)	1965–2007	1.2	12 (9:3)	NA	M^2: 65 (53–82)	M^2: 5 (2.5–11)	2:7:3	NA	NA	NA	7	0	12 [y]:0	NA	M^2: 7.6	1: 5 years 3 mo (S, 2 years after: C+R)
Wong Y.N. [65] (2009, U.K.)	1970–2004	NA	293 (NA)	NA	NA	NA	NA	NA	NA	Na	NA	NA	293 [y]:0	NA	NA	NA
Yamashita H [166] (2009, Japan)	2003–2006	NA	21 [d] (6:3)	NA	M^2: 63 (54–68)	NA	2:5:2	7/0/0/0	NA	9:0	7	0	9 [y]:0	NA	3-year overall survival rate: 56% (4.2–42.8 mo)	2: 34.7, 42.8 mo (C+R)

Table S5. *Cont.*

**Author (Year, Country)**	**Time Period**	**% of Oesophageal Carcinomas that Are NECs**	***N* Oesophageal NECs (Me:W)**	**Predisposing Factors**	**Age Year (Range)**	**Size cm (Range)**	**Location of Primary Tumour (U:M:L)**	**Symptoms (*n* Dysphagia/ Weight Loss/ Pain/Other)**	**Symptom Duration Mo (Range)**	***n* LD: *n* ED**	***n* Lymph. Met.**	***n* Distant Met. at Presentation/ Study Start (Sites)**	***n* SC: *n* LC**	***n* + Other Histological Components**	**Overall Survival Mo (Range)**	***n* Survivors >2 Yearss: Time to Death or End of Follow-Up**
Atsumi K [172] (2010, Japan)	1996–2007	NA	11 (7:4)	NA	M[1]: 69 (44–77)	NA	2:5:4	NA	NA	9:2	8	2 (2 liver, 2 adrenal)	11 [y]:0	NA	M[2]: 13.2 (4.2–43.6)	1: 24 mo (C+R)
Brennan S.M. [6] (2010, Australia)	1985–2007	NA	20 (NA)	NA	NA	NA	NA	NA	NA	NA	NA	NA	20 [y]:0	NA	NA	NA
Gregory D.L. [77] (2010, Australia)	1996–2007	NA	5 (NA)	NA	NA	NA	NA	NA	NA	4:1	NA	NA	5 [y]:0	NA	NA	NA
Kuo CH [145] (2010, Taiwan)	1992–2007	1.0	18 [d] (14:2)	NA	(34–83)	NA	1: 1: 1 (13 mixed)	11/6/5/ 2 odynophagia, 1 hoarsness	NA	8:8	NA	8 (3 liver, 3 lung, 3 abdominal, 2 bone, 2 abdominal, 1 chest wall, 1 neck mass, 1 distant lymph)	16 [y]:0	NA	M[2]: 13.5 (4 days– 221 mo)	3: 34, 75 and 221 mo (all S+C)

Table S5. *Cont.*

Author (Year, Country)	Time Period	% of Oesophageal Carcinomas that Are NECs	*N* Oesophageal NECs (Me:W)	Predisposing Factors	Age Year (Range)	Size cm (Range)	Location of Primary Tumour (U:M:L)	Symptoms (*n* Dysphagia/ Weight Loss/ Pain/Other)	Symptom Duration Mo (Range)	*n* LD: *n* ED	*n* Lymph. Met.	*n* Distant Met. at Presentation/ Study Start (Sites)	*n* SC: *n* LC	*n* + Other Histological Components	Overall Survival Mo (Range)	*n* Survivors >2 Years: Time to Death or End of Follow-Up
Li A.F.Y. [16] (2010, Taiwan)	1989–2008	NA	17 (16:1)	NA	M[1]: 61.8 ± 14.2 SD	NA	NA	NA	NA	9:7	NA	NA	17 [y]:0	NA	M[2]: 15 ± 4.46	NA
Li A.F.Y. [34] (2010, Taiwan)	1989–2009	NA	11 (10:1)	NA	M[1]: 61 ± 13	NA	NA	NA	NA	7:4	NA	4 (NA)	11 [y]:0	4 + squamous cell carcinoma	M[2]: 27.7 ± 14.8	NA
Lu J [175] (2010, China)	1990–2004	4.8 [b]	15 (11:4)	11: smokers + drinkers [j], 6: hot food and quick food [f]	M[1]: 58.8 (40–81)	NA	3:10:2	15/0/5/0	M[1]: 75 days (15 days–10 mo)	NA	3	0	15 [y]:0	6 + squamous dysplasia/ carcinoma insitu, 2 + well differentiated squamous carcinoma	M[2]: 19, M[1]: 23.7 (4–108 mo)	1: 108 mo (S)
Tanaka T [161] (2010, Japan)	1986–2007	0.9	7 (5:2)	NA	(61–72)	(3–8)	1:4:2	NA	NA	4:3	7	3 (3/7 lymph mets; cervical or celiac)	7 [y]:0	NA	M[2]: 16 (12–93)	2: 45, 93 mo (S+ C)
Chen BS [135] (2011, China)	1990–2009	1.0	42 [d] (26:14)	NA	M[1]: 57 (39–77)	M[2]: 5 (2–12)	1:32:7	Dysphagia, weight loss, retrosternal pain (numbers NA)	NA	40:0	27	0	40 [y]:0	NA	M[2]: 13.0	NA

Table S5. *Cont.*

Author (Year, Country)	Time Period	% of Oesophageal Carcinomas that Are NECs	*N* Oesophageal NECs (Me:W)	Predisposing Factors	Age Year (Range)	Size cm (Range)	Location of Primary Tumour (U:M:L)	Symptoms (*n* Dysphagia/ Weight Loss/ Pain/Other)	Symptom Duration Mo (Range)	*n* LD: *n* ED	*n* Lymph. Met.	*n* Distant Met. at Presentation/ Study Start (Sites)	*n* SC: *n* LC	*n* + Other Histological Components	Overall Survival Mo (Range)	*n* Survivors >2 Years: Time to Death or End of Follow-Up
Grossman R.A. [11] (2011, USA)	1973–2006	NA	274 (NA)	NA	NA	NA	NA	NA	NA	NA	NA	NA	247 [y]:0	NA	NA	NA
Vos B [173] (2011, Belgium, the Netherlands, USA, Switzerland)	1994–2004	NA	24 (16:8)	90%: smokers [k], 70%: drinkers [l]	M^2: 65 (59–69)	>5 in 56% of cases (2–8) (5 NA)	3:12:8 (1 NA)	19/13/11/0	NA	11:13 [f]	NA	11 (6 liver, 5 distant lymph, 2 lung, 1 brain, 1 bone, 1 thyroid) 2 NA	24 [y]:0	2 + squamous differentiation	M^2: 11 (8–20)	4: >2 years (1: S+C, 1: S+C+R, 2: NA)
Nakajima Y [154] (2012, Japan)	1996–2006	0.9	18 (13:5)	NA	M^2: 67 (40–70)	NA	4:6:8	NA	NA	10:8	14	9	18 [y]:0	NA	M^2: 16.1	3: 48, 54.2, 76.5 mo (C+R)
Terashima T. [115] (2012, Japan)	2000–2008	NA	8 (NA)	NA	NA	NA	NA	NA	NA	0:8 [y]	NA	8 [y]	8 [y]:0	NA	NA (oesophagus + gallbladder: M^2: 14.9)	NA
Ding J [137] (2013, China)	1998–2007	0.8	212 [d] (66:40)	NA	M^2: 58 (45–77)	NA	NA	NA	NA	106:0 [t]	NA	0	106 [y]:0	NA	S + C or R + C = M^2: 22 S or R = M^2 11	NA

Table S5. *Cont.*

Author (Year, Country)	Time Period	% of Oesophageal Carcinomas that Are NECs	*N* Oesophageal NECs (Me:W)	Predisposing Factors	Age Year (Range)	Size cm (Range)	Location of Primary Tumour (U:M:L)	Symptoms (*n* Dysphagia/ Weight Loss/ Pain/Other)	Symptom Duration Mo (Range)	*n* LD: *n* ED	*n* Lymph. Met.	*n* Distant Met. at Presentation/ Study Start (Sites)	*n* SC: *n* LC	*n* + Other Histological Components	Overall Survival Mo (Range)	*n* Survivors >2 Years: Time to Death or End of Follow-Up
Feng S.T. [82] (2013, China)	2009–2013	NA	7 (NA)	NA	NA	NA	NA	NA	NA	NA	NA	NA	NA	NA	NA	NA
Huang Q [140] (2013, China)	2004–2010	3.8	42 (33:9)	19: tobacco abuse [f], 12: alcohol abuse [f]	M[1]: 62 (47–79)	M[1]: 3.5 (0.2–8)	5:22:15	30/0/11/ 2 hoarseness	NA	NA	20	0	37:5	21 + squamous carcinoma *in situ*, 13 + squamous carcinoma, 6 + glandular differentiation	M[2]: 28.5	2: 90 mo (S)
Hou X. [139] (2013, China)	1994–2011,	1.2%–1.8%	141 (107:34)	NA	M[2]: 58 (38–76)	33 < 5 cm, 77 ≤ 5 cm	14:91:36	NA	NA	141:0	NA	0	141 [y]:0	18 + squamous cell carcinoma, 3 + adenocarcinoma	M[2]: 16.1	NA
Korse C.M. [3] (2013, Netherlands Cancer Registry) [æ]	1990–2010	NA	336 (NA)	NA	NA	NA	NA	NA	NA	NA	NA	NA	NA	NA	NA	NA

Table S5. *Cont.*

**Author (Year, Country)**	**Time Period**	**% of Oesophageal Carcinomas that Are NECs**	***N* Oesophageal NECs (Me:W)**	**Predisposing Factors**	**Age Year (Range)**	**Size cm (Range)**	**Location of Primary Tumour (U:M:L)**	**Symptoms (*n* Dysphagia/ Weight Loss/ Pain/Other)**	**Symptom Duration Mo (Range)**	***n* LD: *n* ED**	***n* Lymph. Met.**	***n* Distant Met. at Presentation/ Study Start (Sites)**	***n* SC: *n* LC**	***n* + Other Histological Components**	**Overall Survival Mo (Range)**	***n* Survivors >2 Years: Time to Death or End of Follow-Up**
Kukar M. [144] (2013, USA)	1973–2009	0.6%	387 (230:157)	NA	M[1]: 68.2	NA	NA	NA	NA	NA	NA	191: distant spread	387 [y]:0	NA	M[2]: 11	NA
Lu X.-J [147] (2013, China)	NA	Mean: 1.26 (0.4–2.8)	1176 (830:346)	NA	M[2]: 57 (25–87)	Average: 5.4 (0.5–17)	121:731:324	78.8%–100%/ NA nr/22.8–15.6%/NA nr hoarseness	M[1]: 1 (10 days–20 mo)	347: 120 [u]	NA	99 [x] (NA)	735 [y]:0 (441 NA)	123 + squamous cell carcinoma, 12 + adenocarcinoma, 3 + adenosquamous carcinoma [z]	M[2]: 11.1 (10–15)	NA
Lu Z.H. [87] (2013, China)	2009–2011	NA	5 (NA)	NA	NA	NA	NA	NA	NA	NA	NA	NA	NA	NA	NA	NA
Meng MB [151] (2013, China)	1991–2009	NA	14 (12:2)	NA	(50–70)	(2–12)	4:10:0	NA	NA	14:0	3	1 (histologic)	14 [y]:0	NA	M[2]: (2–26.3)	1: 26.3 mo (S+C)
Terada T [163] (2013, Japan)	20-years period	0.25	6 (6:0)	NA	M[1]: 73 (62–81)	NA	0:2:4	5/0/0/ 1 vomiting	NA	NA	NA	NA	6 [y]:0	1 + squamous cell carcinoma and adenocarcinoma	M[1]: 13 (2–25)	NA

Table S5. *Cont.*

Author (Year, Country)	Time Period	% of Oesophageal Carcinomas that Are NECs	*N* Oesophageal NECs (Me:W)	Predisposing Factors	Age Year (Range)	Size cm (Range)	Location of Primary Tumour (U:M:L)	Symptoms (*n* Dysphagia/ Weight Loss/ Pain/Other)	Symptom Duration Mo (Range)	*n* LD: *n* ED	*n* Lymph. Met.	*n* Distant Met. at Presentation/ Study Start (Sites)	*n* SC: *n* LC	*n* + Other Histological Components	Overall Survival Mo (Range)	*n* Survivors >2 Years: Time to Death or End of Follow-Up
Sorbye H. [4] (2013, 12 Nordic hospitals)	2000–2009	NA	12 (NA)	NA	NA	NA	NA	NA	NA	NA	NA	NA	75%: NA	NA	M^2: 14 (CI 95%: 2.3–25.7)	NA
Wang SY [164] (2013, China)	2001–2011	1.3	76 (60:16)	39: tobacco + alcohol abuse [f], 12: tobacco abuse [f], 4: alcohol abuse [f]	M^2: 61 (41–84)	>5 cm: 32, ≤5 cm: 44	16:46:14	57/36/25/ 2 bleeding, 2 cerchnus, 1 other	NA	60:16	44	17 (6 liver, 7 distant lymph nodes, 3 lung, 3 bone, 1 pancreas, 1 stomach)	76 [y]:0	14 + squamous differentiation, 1 + sarcomatoid differentiation	M^2: 15.77 (95% CI: 11.0–15.77)	4: >5 years (2: S+C, 2: C+R)
Yan H. [97] (2013, China)	1992–2012	NA	82 (53:29)	NA	44 ≤ 60 years, 37 > 60 years	48 ≤ 5 cm, 34 > 5 cm	29:37:16	NA	NA	NA	46	31	82 [y]:0	NA	M^2: Me: 12, W: 14	NA
Chen W.W. [177] (2014, China)	1998–2012	NA	211 (164:47)	NA	154 < 60 years, 57 ≥ 60 years	73 < 5 cm, 124 ≥ 5 cm	31:124:56	NA	NA	148: 63 [v]	145	60	211 [y]:0	NA	M^2: 13 mo	NA
Chen W.W. [186] (2014, China)	1994–2012	1.7%	44 (33:11)	NA	15 > 60 years, 29 ≤ 60 years	21/39 < 5 cm, 18/39 ≥ 5 cm	7:22:15	NA	NA	NA	26	10	44 [y]:0	NA	High level Lgr5: M^2: 7 mo, Low level: M^2: 18 mo	18: >2 years

Table S5. *Cont.*

**Author (Year, Country)**	**Time Period**	**% of Oesophageal Carcinomas that Are NECs**	***N* Oesophageal NECs (Me:W)**	**Predisposing Factors**	**Age Year (Range)**	**Size cm (Range)**	**Location of Primary Tumour (U:M:L)**	**Symptoms (*n* Dysphagia/ Weight Loss/ Pain/Other)**	**Symptom Duration Mo (Range)**	***n* LD: *n* ED**	***n* Lymph. Met.**	***n* Distant Met. at Presentation/ Study Start (Sites)**	***n* SC: *n* LC**	***n* + Other Histological Components**	**Overall Survival Mo (Range)**	***n* Survivors >2 Years: Time to Death or End of Follow-Up**
Okuma H.S. [182] (2014, Japan)	2000–2013	NA	12 (8:4)	NA	M^2: 62 (39–72)	NA	NA	NA	NA	0:12	NA	12 (9 liver, 6 lymph, 1 lung, 1 bone, 1 bone marrow, 1 stomach, 1 crural muscle)	NA	NA	M^2: 12.6 (1.1–46.2)	3: 28.6, 32, 46)
Olsen I.H. [124] (2014, Denmark)	3-year period	NA	8 (NA)	NA	NA	NA	NA	NA	NA	NA	NA	8	NA	NA	NA	NA
Yamaguchi T. [92] (2014, Japan)	2000–2011	NA	85 [e] (NA)	NA	NA	NA	NA	NA	NA	NA	NA	NA	NA	NA [e]	M^2: 13.4	NA
Zhang Z. [168] (2014, China)	2007–2012	NA	38 (31:7)	NA	M^2: 61.3 (42–76)	NA	4:23:11	NA	NA	NA	NA	NA	38 [y]:0	NA	NA	NA
Zhu Y. [176] (2014, China)	1990–2011	0.94%	64 (46:18)	35: smoking index >400 [f], 27: risky diets (heavy drinking, hot food, bulky food) [f]	M^2: 58 (43–76)	M^2: 5 (1–12)	9:35:16 (4 mixed)	52/0/NA nr/ NA nr hoarsness, abdominal distension, cervial mass	NA	46:18	49	18 (5: liver mets, 2: lung, 1: bone, 1: thyorid)	64 [y]:0	6 + squamous cell carcinoma, 2 + cardiac adenocarcinoma	M^2: 12.6	4: >4 years (1: S, 1: S+C, 1: R+C, 1: R+S+C)

Table S5. *Cont.*

Author (Year, Country)	Time Period	% of Oesophageal Carcinomas that Are NECs	*N* Oesophageal NECs (Me:W)	Predisposing Factors	Age Year (Range)	Size cm (Range)	Location of Primary Tumour (U:M:L)	Symptoms (*n* Dysphagia/ Weight Loss/ Pain/Other)	Symptom Duration Mo (Range)	*n* LD: *n* ED	*n* Lymph. Met.	*n* Distant Met. at Presentation/ Study Start (Sites)	*n* SC: *n* LC	*n* + Other Histological Components	Overall Survival Mo (Range)	*n* Survivors >2 Years: Time to Death or End of Follow-Up
TOTAL	(1947–2011)	Average: 1.6 [c]	3806 [c] (Me: 1897/2704 (70%), W: 807/2704 (30%)	At least 196 smokers or drinkers or both of 254 observed (77.2%)	(25–89)	(0.2–17)	250:1240: 636 (of 2126 [c, m])	411/119/111 [o]/ 44 (Of 680 symptoms observed in 525 patients [c, n])	(0–20)			66 (36%) liver, 57 (31.3%) distal lymph nodes, 20 (11%) lung, 16 (8.8%) bone, 3 (1.6%) brain, 18 (9.9%) other (of 182 mentioned)		225 + squamous (81.5%), 40 + adeno/muco/ glandular (14.5%), 11 + other (4.0%) (of 276 mentioned)		81/2080 (3.9%) [c]

[a] includes oesophageal and proximal gastric cancer; [b] includes only superficial carcinomas of the oesophagus; [c] possible overlapping cohorts with lowest patient number not included. NA not included; [d] lower number of patients included in the study/with complete records, as reflected in the total of (M:W); [e] orginal cohort of 258 included 21 MANECs, distribution between organ sites is not known; [f] no further specification; [g] excess of 80 g alcohol per day; [h] ≥1 pack of cigarettes/day for >2 years; [i] >2 pints of beer (or the equivalent)/day for >2 years; [j] moderate to severe degree for >10 years; [k] mean of 40 pack-years (range: 20–50); [l] mean of 3 drinks/day (range 2–5); [m] other categorization not included; [n] only precise numbers; [o] includes reflux; [p] LD includes mediastinal lymph node involvement; [q] LD encompasses stages I–III and no metastases; [r] ED includes intra-abdominal lymph nodes in patients with mid-third oesophageal tumours; [s] ED is the equivalent to AJCC stage IVB; [t] LD: visable tumour within a radiation field, ED: visable tumour exceeded beyond LD; [u] Of 467 cases studied; [v] VALSG (no further information); [w] Unknown if any of the lymph metastases are distant; [x] Of 523 studied; [y] part of inclusion criteria; [z] Of 735 analyzed; [å] Includes patients alive without recurrence, alive with recurrence and those who have died of the disease/other disease 2 years after diagnosis/treatment start; [æ] Numbers may not be exact; extrapolated from incidence rates [ø] Prospective study, all others retrospective or NA; NA: Not Available; NECs: includes small cell/large cell carcinomas, WHO 2010/WHO 2000 definition of gastroenteropancreatic neuroendocrine carcinoma/poorly differentiated endocrine carcinoma/ G3 tumours; Me: Men; W: Women; M[1]: Mean; M[2]: Median; Year: years/years-of-age; U: Upper oesophagus; M: Middle oesophagus; L: Lower oesophagus; SAIDH: Syndrome of inappropriate antidiuretic hormone; Mo: months; LD: tumour confined to a localized anatomic region (the oesophagus/perioesophageal tissue/adjacent organs) with/without locoregional lymph nodes metastases (encompassable within a tolerable radiation therapy treatment portal), ED: outside LD; SC: Small cell, LC: Large cell; Met.: Metastases; CI: Confidence interval; C: Chemotherapy; S: Surgery; R: Radiotherapy.

Table S6. Overview of Studies Including Gastric Neuroendocrine Carcinomas.

Author (Year, Country)	Time Period	% of NECs in Relation to Gastric Cancers	*n* Gastric NEC (M:W)	Age (Range)	Size cm (Range)	Location in Primary	*n* Patients with Lymph. Met.	*n* Patients with Distant Mets. at Presentation (Sites)	*n* SC: *n* LC	Other Histological Components	Overall Survival Mo (Range)	Long-Term Survivors (>2 Years) (DOD, AWD, NED) *n*: Time until Death/End of Follow-Up Mo
Rindi G. [197] (1993, Italy)	1970–NA	NA	9.00	M[1]: 61.3 ± 10 years	M[2]: 3.5 (1.5–7)	2 antrum. 2 body-fundus, 5 NA	NA (4 local lymph, 3 local + distant sites, 2 distant only)	5	6	NA	M[1]: 6.5 (1–12)	0
Rindi G. [198] (1996, Italy)	1970–NA	NA	12.00	M[1]: 63 (41–76)	M[1]: 4.2 (1.5–7)	10 body/fundus, 2 antrum	6 local lymph, 3 local + distant, 3 distant sites	6	NA	3: 2+ well differentiated argyrophil, 1 poorly + well differentiated	M[1]: 6.6 (1–12)	3: 72 mo M[1] follow-up (13–168)
Matsui K [194] (1998, Japan)	1987–1996	1.22 [a]	33 (23:10)	M[2]: 60 (44–92)	M[2]: 6.0 (2–14)	15 cardia: 8 M: 10 antrum	32	8 (8 liver, unknown if others)	12:21	19 + adenocarcinoma, 2 + squamous component and adenocarcinoma	M[1]: 14.9 [b]	1: >4 years
Kimura H [205] (1999, Japan)	1973–1997	0.23 [c]	8 (7:1)	M[1]: 62.6 (44–73)	M[1]: 7.9 (4–12)	1: M, 3: antrum, 2: cardia + M, 1: antrum + M, 2: M + cardia + antrum	8	3 (sites NA)	8: 0	NA	Estimated M[2]: 252 days (45–1520 days)	1: 43
Kimura H. [14] (1999, Japan)	1981–1994	NA	8 (NA)	(44–73)	(4–12)	NA	8	3 (sites NA)	8 [d]:0	NA	M[2]: 253 days (45–822 days)	2: 747 days, 822 days
Rindi G. [63] (1999, Italy)	NA [e]	NA	16 (13:3)	NA	M[2]: 4.25 (3–5.55)	0 U: 9 M : 7 antrum	15	8	NA	NA	M[2]: 8	NA

Table S6. *Cont.*

Author (Year, Country)	Time Period	% of NECs in Relation to Gastric Cancers	*n* Gastric NEC (M:W)	Age (Range)	Size cm (Range)	Location in Primary	*n* Patients with Lymph. Met.	*n* Patients with Distant Mets. at Presentation (Sites)	*n* SC: *n* LC	Other Histological Components	Overall Survival Mo (Range)	Long-Term Survivors (>2 Years) (DOD, AWD, NED) *n*: Time until Death/End of Follow-Up Mo
Kim K.M. [62] (2002, Korea)	NA	NA	6 (5:1)	(61–81)	(2.5–10.2)	5: antrum, 1: body + antrum	NA	1 (NA others)	0: 6	6 + adenocarcinoma	NA	NA
Nishikura K [196] (2003, Japan)	1980–2000	NA	68 [f] (54:14)	Average: 66.5 [f]	NA	NA	NA	NA	NA	48 + adenocarcinoma [f]	NA	NA
Pizzi S. [38] (2003, Italy)	NA	NA	15 (13:2)	(40–91)	NA	NA	10, 2 NA	5 (3 liver, 3 peritoneum, 1 bone) [g]	NA	NA	NA	NA
Brenner B. [7] (2004, USA)	1980–2002	NA	5 (NA)	NA	NA	NA	NA	NA	5 [d]:0	NA	NA	NA
Furlan D. [39] (2004, Italy)	NA	NA	6 (6:0)	(37–72)	(1.2–14)	NA	5	1 (liver), (1 NA) [g]	NA	NA	Average: 15.7 (5–48)	1: 48
La Rosa S. [58] (2004, Italy)	NA	NA	7 (7:0)	M^1: 56 (38–74)	M^1: 7.6 (1.2–14)	NA	NA	7	NA	NA	NA	NA
Borch K [188] (2005, Sweden) [m]	1994–1999	NA	9 (7:2)	M^2: 72 (20–80)	M^2: 4.3 (1.5–10.0)	NA	NA	7 (7 liver)	NA	NA	5 years survival: 33.3% (2.5%–64.1%)	33.3% alive at 5 years

Table S6. *Cont.*

Author (Year, Country)	Time Period	% of NECs in Relation to Gastric Cancers	*n* Gastric NEC (M:W)	Age (Range)	Size cm (Range)	Location in Primary	*n* Patients with Lymph. Met.	*n* Patients with Distant Mets. at Presentation (Sites)	*n* SC: *n* LC	Other Histological Components	Overall Survival Mo (Range)	Long-Term Survivors (>2 Years) (DOD, AWD, NED) *n*: Time until Death/End of Follow-Up Mo
Furlan D. [43] (2005, Italy)	NA	NA	7 (6:1)	(37–72)	(1.2–14)	NA	6 (1 NA)	1 (liver) (1 NA) [g]	6 intermediate, 1 large/ intermediate	NA	(5–48)	1: 48
Pizzi S. [55] (2005, Italy)	NA	NA	8 (7:1)	(44–83)	(0.4–10)	NA	5 (1 NA)	3 (3 liver, 2 peritoneum) (1 NA) [g]	NA	NA	NA	NA
Ishikubo T. [54] (2006, Japan)	NA	NA	16 (13:3)	(51–79)	NA	NA	NA	NA	NA	NA	NA	NA
Jiang SX. [191] (2006, Japan)	1980–2004	1.48 [h]	42 (34:8)	M[1]: 62.7 (47–79)	M[1]: 6.4 (1.1–13.0)	15 U: 11 M: 16 L	30 (1 NA)	3 (3 liver)	0: 42 [d] (inclusion)	NA	5 years survival: 31.1%	NA
Kim K.O. [13] (2006, Korea)	1998–2005	NA	5 (NA)	NA	NA	NA	NA	NA	5 [d]:0	NA	NA	NA
Li A.F.Y. [42] (2006, Taiwan)	1985–2004	NA	9 (8:1)	(34–88)	NA	NA	7	2	NA	NA	(4–91), 1 NA	3: 91, 65, 25
Uccella S. [37] (2006, Italy)	1982–2004	NA	11 (10:1)	(41–72)	(3–14)	NA	NA	NA	NA	NA	NA	NA

Table S6. *Cont.*

Author (Year, Country)	Time Period	% of NECs in Relation to Gastric Cancers	*n* Gastric NEC (M:W)	Age (Range)	Size cm (Range)	Location in Primary	*n* Patients with Lymph. Met.	*n* Patients with Distant Mets. at Presentation (Sites)	*n* SC: *n* LC	Other Histological Components	Overall Survival Mo (Range)	Long-Term Survivors (>2 Years) (DOD, AWD, NED) *n*: Time until Death/End of Follow-Up Mo
Boo YJ [187] (2007, Korea)	1993–2000	NA	12 (10:2)	Average: SC: 63.6 (±8), LC (64.8 (±11)	M[1]: SC: 4.28 (±3.36), LC: 6.00 (± 4.06)	2 U: 2 M: 8 L	NA	5	7:5	NA	15 (CI 95% 1.2–28.8)	NA
Lee H. [70] (2007, Korea)	1996–2005	NA	9 (NA)	NA	NA	NA	NA	NA	4:5	NA	NA	NA
Lepage C. [1] (2007, UK)	1986–1999	NA	112 (NA)	NA	NA	NA	NA	NA	112 [d]:0	NA	Relative 1 year survival: 17.6%	NA
Landry CS [204] (2008, USA)	1973–2004	NA	46 (NA)	NA	NA	NA	NA	NA	NA	NA	NA	NA
Shia, J. [29] (2008, USA)	NA	NA	18 (12 gastric + 6 gastro-oesophageal junction) (NA)	NA	NA	NA	NA	NA	7:10 (+1 mixed)	NA	NA	NA
Lepage C. [79] (2009, EUROCARE)	1985–1994	NA	123	NA	NA	NA	NA	NA	123 [d]:0	NA	5 years relative survival: 7.7	NA

Table S6. *Cont.*

Author (Year, Country)	Time Period	% of NECs in Relation to Gastric Cancers	*n* Gastric NEC (M:W)	Age (Range)	Size cm (Range)	Location in Primary	*n* Patients with Lymph. Met.	*n* Patients with Distant Mets. at Presentation (Sites)	*n* SC: *n* LC	Other Histological Components	Overall Survival Mo (Range)	Long-Term Survivors (>2 Years) (DOD, AWD, NED) *n*: Time until Death/End of Follow-Up Mo
Wong Y.N. [65] (2009, England)	1970–2004	NA	92 (NA)	NA	NA	NA	NA	NA	92 [d]:0	NA	NA	NA
Kim BS [192] (2010, Korea)	1993–2008	NA	52 (45:7)	M^2: 62.1 (±11.1)	M^1: 5.44 (±3.7)	14 U: 10 M: 28 L	23	13	NA	17 + adenocarcinoma	5 years survival: 41.6%	41.6% alive at 5 years
Li A.F.Y. [16] (2010, Taiwan)	1989–2008	NA	8 (7:1)	M^1:68.0 SD: ±11.1	NA	NA	NA	3 ED	8 [d]:0	NA	M^2: 15 (SD ± 49.97)	50% alive at 2 years
Li A.F.Y. [34] (2010, Taiwan)	1989–2009	NA	15 (15:0)	M^1: 75 ± 8	NA	NA	NA	7 ED	15 [d]:0	4 + adenocarcinoma	M^2: 21.3 ± 29.0 (M^1 SD)	NA
Grossman R.A. [11] (2011 USA)	1973–2006	NA	147 (NA)	NA	NA	NA	NA	NA	147 [d]:0	NA	NA	NA
La Rosa S [61] (2011, Italy)	1980–2009	NA	51 (NA)	NA	M^2: 5.55 (7 NA)	NA	NA	38 (9 NA)	51:0	12 + non-neuroendocrine glandular/solid growth	”Rate”: 75.0 (7 NA)	NA
Okita N.T [206] (2011, Japan)	2000–2006	NA	22 (19:3)	(27–82)	NA	NA	NA	8	20:2	4 + adenocarcinoma	M^2: 33	2: >3 years

Table S6. *Cont.*

Author (Year, Country)	Time Period	% of NECs in Relation to Gastric Cancers	*n* Gastric NEC (M:W)	Age (Range)	Size cm (Range)	Location in Primary	*n* Patients with Lymph. Met.	*n* Patients with Distant Mets. at Presentation (Sites)	*n* SC: *n* LC	Other Histological Components	Overall Survival Mo (Range)	Long-Term Survivors (>2 Years) (DOD, AWD, NED) *n*: Time until Death/End of Follow-Up Mo
Annenkov A [64] (2012, Japan)	1985–2011	NA	51 [i] (40:11)	Average: 69.6 (SD ± 9.8) [i]	M^2: 4.85 (quartile range 3–6.1) [i]	NA	33 [i]	NA	NA	37 + adenocarcinoma [i]	NA	NA
Endo S. [28] (2012, Japan)	1998–2011	0.25	7 (5:2)	(46–72)	(1.7–9.8)	NA	5	2	NA	NA	Average: 50.3 (1–122)	3: 24, 62, 122
Faggiano A. [68] (2012, Italy)	1990–2007	NA	7 (NA)	NA	NA	NA	NA	NA	NA	NA	NA	NA
Huang J [189] (2012, China)	1990–2011	0.16	19 (17:2)	M^2: 61 (38–73)	M^1: 7.3 (2–15)	9: cardia, 4: cardia + fundus, 1: corpus + antrum, 1: corpus + fundus, 1: cardica + fundus + corpus + antrum	15	2 (1 liver, 1 retroperitoneal lymph nodes)	19:0	6 + adenocarcinoma	M^2: 19.5	4: 25.5, 36.6, 48.5, 227.8
Kubota T [193] (2012, Japan)	1986–2008	0.4 [f]	27 [f] (20:7)	Average: 66.3 (±8.6) [f]	Average: 7.27 (±4.43) [f]	1 U: 5 M: 10 L 1: U+M+L [f]	20 [f]	9 (4 liver, 2 peritoneum, 3 distant lymph) [f]	NA	NA [f]	5 years survival: 43.8% [f]	43.8% alive at 5 years [f]

Table S6. *Cont.*

Author (Year, Country)	Time Period	% of NECs in Relation to Gastric Cancers	*n* Gastric NEC (M:W)	Age (Range)	Size cm (Range)	Location in Primary	*n* Patients with Lymph. Met.	*n* Patients with Distant Mets. at Presentation (Sites)	*n* SC: *n* LC	Other Histological Components	Overall Survival Mo (Range)	Long-Term Survivors (>2 Years) (DOD, AWD, NED) *n*: Time until Death/End of Follow-Up Mo
Terashima T. [115] (2012, Japan)	2000–2008	NA	10 (NA)	NA	NA	NA	NA	NA	10 [d]:0	NA	NA (oesophagus + gallbladder: M[2] 14.9 mo)	NA
Du Z. [81] (2013, China)	2008–2010	NA	6 (5:1)	(47–74)	NA	NA	3	4 (3 liver, 1 lung, 1 peritoneum, NA where lymph mets are)	NA	N	(6–24)	0
Feng S.T. [82] (2013, China)	2009–2013	NA	16 (NA)	NA	NA	NA	NA	NA	NA	NA	NA	NA
Ishida M [190] (2013, Japan)	1990–2011	0.64 [j]	51 [j] (39:12)	M[2]: 70 (35–86) [j]	M[2]: 5 (1.5–15) [j]	20 U: 18 M: 13 L [j]	36 [j]	13 (4 distant lymph nodes, 4 liver, 4 peritoneum) [j]	11:40 [j]	7: mixed SC and LC, 36: adenocarcinoma and/or dysplasia [j]	3 years survival: 57.8% [j]	57.8% alive after 3 years
Kim BS. [295] (2013, Korea)	1997–2012	NA	13 (10:3)	M[2]: 58 (42–81)	NA	4 U: 3 M: 6 L	4	4 (NA)	13 [d]:0	NA	M[1]: 40.3 (1.2–146.5)	4: 65.9, 98.2, 114.6, 146.5
Korse C.M. [3] (2013, Netherlands Cancer Registry) [n]	1990–2010	NA	203 (NA)	NA	NA	NA	NA	NA	NA	NA	5 years relative survival rate: LC 1990–2000/ 2001–2010: 34/32%. SC 1990–2000/ 2001–2010: 5/6%	NA

Table S6. *Cont.*

**Author (Year, Country)**	**Time Period**	**% of NECs in Relation to Gastric Cancers**	***n* Gastric NEC (M:W)**	**Age (Range)**	**Size cm (Range)**	**Location in Primary**	***n* Patients with Lymph. Met.**	***n* Patients with Distant Mets. at Presentation (Sites)**	***n* SC: *n* LC**	**Other Histological Components**	**Overall Survival Mo (Range)**	**Long-Term Survivors (>2 Years) (DOD, AWD, NED) *n*: Time until Death/End of Follow-Up Mo**
Lu Z.H. [87] (2013, China)	2009–2011	NA	8 (NA)	NA	NA	NA	NA	NA	NA	NA	NA	NA
Mia-Jan K. [52] (2013, Korea)	2000–2012	NA	5 (NA)	NA	NA	NA	NA	NA	NA	NA	NA	NA
Namikawa T [195] (2013, Japan)	2004–2011	NA	5 (4:1)	M^2: 72 (52–84)	M^2: 6 (2–13)	1 U: 2 M: 2 L	5	3 (2 perinoneum, 1 liver)	5:0	3 + adenocarcinoma	5 years survival: 60% (2–84 mo)	2: 53, 84
Sorbye H. [4] (2013, 12 Nordic hospitals)	2000–2009	NA	20 (NA)	NA	NA	NA	NA	NA	NA	NA	M^2: 11 (CI 95%: 7.1–14.9)	NA
van der Zwan J.M. (2013 RARECARE, Europe) [284]	1995–2002	NA	143	NA	NA	NA	NA	NA	NA	NA	NA	NA

Table S6. *Cont.*

Author (Year, Country)	Time Period	% of NECs in Relation to Gastric Cancers	*n* Gastric NEC (M:W)	Age (Range)	Size cm (Range)	Location in Primary	*n* Patients with Lymph. Met.	*n* Patients with Distant Mets. at Presentation (Sites)	*n* SC: *n* LC	Other Histological Components	Overall Survival Mo (Range)	Long-Term Survivors (>2 Years) (DOD, AWD, NED) *n*: Time until Death/End of Follow-Up Mo
Yamaguchi T. [92] (2014, Japan)	2000–2011	NA	70 [k] (NA)	NA	NA	NA	NA	NA	NA	NA [k]	M[2]: 13.3 [k]	NA
TOTAL	1970–2012	0.16–1.48	937 [l]	(27–92)	M[2]: 4.85–6 (1.5–15), M[1]: 4.28–6.4 (0.4–13) [l]	-	235/335 (4 NA) [l]	119/344 (sites where mentioned: 60 sites mentioned: 38 liver, 12 peritoneum, 8 distant lymph nodes, 1 bone, 1 lung) [l]	-	-	-	-

NEC: Neuroendocrine Carcinoma: includes WHO 2000/2004/2010 definition (poorly differentiated endocrine carcinomas, G3 tumours, small cell and large cell carcinomas); NA: Not Available; M: Men; W: Women; M[1]: Mean; M[2]: Median; Mo: Month/s; Year: Year/s; SC: Small Cell; LC: Large Cell; Met.: Metastases; CI: Confidence Intervall; DOD: Dead of disease; AWD: Alive with disease; NED: No evidence of disease; U: Upper stomach; M: Middle stomach; L: Lower stomach; LD: tumour contained within a localised anatomic region, with or without regional lymph node involvement, ED: beyond LD; [a] % of surgically resected gastric cancers; [b] 1 long survivor and 4 patients who died post-op are not in survival equation; [c] % of all patients who underwent a gastrectomy due to gastric cancer; [d] part of inclusion criteria; [e] "Last three decades"; [f] classified after the Japanese Classification of Cancer: ECC; includes both NEC and MANEC (unknown distribution!); [g] Unknown if lymph node metastases could also be distant metastases; [h] Large cell carcinomas only; [i] cohort includes 12 gastric MANECs, unknown distribution; [j] cohort includes 13 gastric MANECs, unknown distribution; [k] in original cohort of 258 NEC, 21 were MANECs; unknown distribution amoung primary sites; [l] after removal of possible overlapping cohorts with lowest patient numbers, and known number MANECs. Can still include an unknown number of MANECs; [m] prospective study, all other NA or retrospective; [n] not all numbers may be exact due to extrapolation from incidence rates.

Table S7. Overview of Studies Including Pancreatic Neuorendocrine Carcinomas.

Author (Year, Country)	Time Period	% of PNENs Diagnosed as NECs	*n* Pancreatic NEC (M:W)	Age Year (Range)	Size cm (Range)	Location in Primary: *n* Caput: *n* Corpus:*n* Cauda	*n* Lymph. Met.	*n* Distant Met. at Presentation/Study Start (Sites)	*n* SC:*n* LC	Overall Survival Mo (Range)	Long-Term Survivors (>2 Years) *n*: Mo Survival (DOD, AWD or NED)
Reyes C.V. [207] (1980, USA)	1951–1979	NA	5 (5:0)	M[1]: 60 (42–73)	Average: 4.2	4:1:0	5	NA (4 liver, 3 lungs, 1 spleen, 1 gallbladder, 1 adrenal gland, 1 cerebrum) (NA nr distant lymph metastases)	5:0	(1–2)	0
Moertel C.G. [114] (1991, USA)	1987–1990	NA	6 (NA)	NA	NA	NA	NA	6 [k] (NA)	NA	NA	NA
Pelosi G. [231] (1996, Italy)	1974–1993	14.8	8 (4:4)	(25–65)	(1.5–9)	NA	5	3 (2 liver, 1 retroperitoneal lymph node)	NA	(1–23) 1 NA	0
Galanis E. [9] (1997, USA)	1974–1994	NA	7 (NA)	NA	NA	NA	NA	6 [l] (NA)	7 [k]:0	NA	NA
Madeira I [221] (1998, France)	1991–1997	12.3	9 (NA)	NA	NA	NA	NA	8 (8 liver, others NA)	NA	M[2]: 5	0
Mitry E. [88] (1999, France)	1988–1997	NA	13 (NA)	NA	NA	NA	NA	10 (NA)	NA	NA	NA
Brenner B. [7] (2004, USA)	1980–2002	NA	6 (NA)	NA	NA	NA	NA	NA	6 [k]:0	NA	NA
Artale S. [107] (2005, Italy) [a]	2000–2003	NA	5 (NA)	NA	NA	NA	NA	5 [k] (NA)	NA	NA	NA
Couvelard A [235] (2005, France)	1995–2001	17.8	8 (NA)	NA	NA	NA	NA	NA	NA	NA	NA

Table S7. *Cont.*

Author (Year, Country)	Time Period	% of PNENs Diagnosed as NECs	*n* Pancreatic NEC (M:W)	Age Year (Range)	Size cm (Range)	Location in Primary: *n* Caput: *n* Corpus:*n* Cauda	*n* Lymph. Met.	*n* Distant Met. at Presentation/Study Start (Sites)	*n* SC:*n* LC	Overall Survival Mo (Range)	Long-Term Survivors (>2 Years) *n*: Mo Survival (DOD, AWD or NED)
Haider K. [12] (2006, Canada)	1971–2002	NA	7 (6:1)	NA	NA	NA	NA	6 [l] (NA)	7 [k]:0	NA	NA
Kaifi J.T. [218] (2006, Germany)	NA	14.2	9 (NA)	NA	NA	NA	NA	NA	NA	NA	NA
Kim K.O. [13] (2006, Daegu, Korea)	1998–2005	NA	5 (NA)	NA	NA	NA	NA	NA	5 [k]:0	NA	NA
Rodallec M. [104] (2006, France)	1993–2002	18.9	7 (NA)	NA	M[1]: 6 (2–9)	3 (+1 isthmus): 3 corpus/cauda	NA	NA	NA	(1–52)	1: 52
Bilimoria KY [233] (2007, USA)	1985–2004	22.1 [b]	268 (NA)	NA	NA	NA	NA	NA	NA	NA	NA
Faggiano, A [76] (2007, France)	1990–2001	NA	6 (NA)	NA	NA	NA	NA	NA	0: 6 [k]	NA	NA
Lepage C. [1] (2007, UK)	1986–1999	NA	68 (NA)	NA	NA	NA	NA	NA	68 [k]:0	Relative 1 year survival: 17.3%	NA
Ploeckinger U. [73] (2007, Germany)	1999–2007	NA	28 (NA)	NA	NA	NA	NA	NA	NA	NA	NA
Sellner F. [229] (2007, Austria)	1985–2005	NA	11 (NA)	NA	NA	NA	6 (2 NA)	40% (NA)	NA	5 years survival: 0%	1: 24

Table S7. *Cont.*

Author (Year, Country)	Time Period	% of PNENs Diagnosed as NECs	*n* Pancreatic NEC (M:W)	Age Year (Range)	Size cm (Range)	Location in Primary: *n* Caput: *n* Corpus:*n* Cauda	*n* Lymph. Met.	*n* Distant Met. at Presentation/Study Start (Sites)	*n* SC:*n* LC	Overall Survival Mo (Range)	Long-Term Survivors (>2 Years) *n*: Mo Survival (DOD, AWD or NED)
Chung J.C. [285] (2007, Korea)	1995–2004	27.3	5 (NA)	NA	NA	NA	NA	NA	NA	5 years survival: 41.7%	41.7% alive at 5 years
Bilimoria KY [211] (2008, USA)	1985–2004	22.1	268 (NA)	NA	NA	NA	NA	NA	NA	5 years survival: 28.3%	28.3% alive at 5 years
Bettini R. [242] (2008, Italy)	1990–2004	9	17 (NA)	NA	NA	NA	NA	NA	NA	M^2: 11.8 (95% CI: 5.5–18.1)	NA (All died within 3.5 years)
Ekeblad S [213] (2008, Sweden)	1967–2005	8.7	21 (NA)	NA	NA	NA	NA	NA	1: NA	(NA–150)	NA
Fischer L [216] (2008, Germany) [a]	1994–2006	11	13 (10:3)	M^2: 56.8 (13–90)	M^2: 3 (3–5)	9:2:2	9	7 (NA)	NA	(NA–30)	3: 20–30
Halfdanarson T.R. [286] (2008, USA)	1973–2000	48.5 [c]	146 (NA)	NA	NA	NA	NA	NA	NA	M^2: 7.5	NA
Pape U.F. [223] (2008, Germany)	1980–2003	16	21 (12:9)	NA	NA	NA	NA	NA	NA	NA	NA
Bettini R. [244] (2009, Italy)	1990–2004	9.8	5 (NA)	NA	NA	NA	NA	5 [k] (all liver, others NA)	NA	M^2: 11.8 (95% CI: 0–24.5)	NA
Casadei R [287] (2009, Italy)	1976–2008	15.8	12 (NA)	NA	NA	NA	NA	NA	NA	NA	NA

Table S7. *Cont.*

Author (Year, Country)	Time Period	% of PNENs Diagnosed as NECs	*n* Pancreatic NEC (M:W)	Age Year (Range)	Size cm (Range)	Location in Primary: *n* Caput: *n* Corpus:*n* Cauda	*n* Lymph. Met.	*n* Distant Met. at Presentation/Study Start (Sites)	*n* SC:*n* LC	Overall Survival Mo (Range)	Long-Term Survivors (>2 Years) *n*: Mo Survival (DOD, AWD or NED)
Figueiredo F.A.F. [215] (2009, France)	1999–2008	26.7	26 [g] (NA)	NA	NA	NA	NA	NA	NA	M[1]: 42 (24–61)	16: 24–61
La Rosa S. [219] (2009, Italy)	1978–2001	3,9	6 (NA)	NA	NA	NA	NA	NA	NA	M[2]: 6 (5/6 patient), 1: 36 mo	1: 36
Lepage C. [79] (2009, EUROCARE database)	1985–1994	NA	109	NA	NA	NA	NA	NA	109 [k]:0	5 years relative survival: 5.3	NA
Wong Y.N. [65] (2009, England)	1970–2004	NA	55 (NA)	NA	NA	NA	NA	NA	55 [k]:0	NA	NA
You D.D. [227] (2009, Korea)	1995–2007	7.3	7 (4:3)	(33–71)	(2–11.5)	NA	3	2 (NA)	NA	2 years survival: 19% (8–29 mo)	1: 29
Catena L. [57] (2010, Italy)	1984–2009	NA	12 (NA)	NA	NA	NA	NA	NA	NA	NA	NA
Franko J. [243] (2010, USA)	1973–2004	38.6 [d]	237 (NA)	NA	NA	NA	NA	NA	NA	M[2]: 7	NA
Iwasa S. [246] (2010, Japan)	1995–2009	NA	10 (NA)	NA	NA	NA	NA	NA	NA	M[2]: 6.2	NA
Niederle M.B. [66] (2010, Austria) [a]	2004–2005	NA	5 (NA)	NA	NA	NA	NA	NA	NA	NA	NA

Table S7. *Cont.*

Author (Year, Country)	Time Period	% of PNENs Diagnosed as NECs	*n* Pancreatic NEC (M:W)	Age Year (Range)	Size cm (Range)	Location in Primary: *n* Caput: *n* Corpus:*n* Cauda	*n* Lymph. Met.	*n* Distant Met. at Presentation/Study Start (Sites)	*n* SC:*n* LC	Overall Survival Mo (Range)	Long-Term Survivors (>2 Years) *n*: Mo Survival (DOD, AWD or NED)
Scarpa A. [237] (2010, Italy)	1991–2005	10.2	28 (NA)	NA	NA	NA	NA	NA	NA	M^2: 1.3 years (CI 95% 0.8–1.8)	12% alive at 5 years
Turner N.C. [119] (2010, UK) [a]	1999–2008 [a]	NA	5 (NA)	NA	NA	NA	NA	NA	NA	NA	NA
Zerbi A. [232] (2010, Italy) [a]	2004–2008	7.7	23 (NA)	M^1: 63.2	M^2: 4	NA	NA	14 (NA)	NA	NA	NA
Arvold N.D. [209] (2011, USA)	1980–2010	25	6 (NA)	NA	NA	NA	NA	NA	NA	NA	NA
Amato E [240] (2011, Italy)	NA	5	6 (NA)	NA	NA	NA	NA	NA	NA	NA	NA
Bettini R. [288] (2011, Italy)	1990–2008	4.5	8 (NA)	NA	4 ≤2 cm, 1 >2–4 cm, 3 > 4 cm	NA	NA	NA	NA	NA	NA
Hentic O. [100] (2011, France)	1997–2006	NA	8 (NA)	NA	NA	NA	NA	8 [k] (NA)	NA	NA	NA
Liszka L [220] (2011, Poland)	2001–2010	14	7 (NA)	NA	NA	NA	NA	NA	NA	NA	NA
Panzuto F. [238] (2011, Italy)	1998–2008	14.9	36 [h] (NA)	NA	NA	NA	NA	NA	NA	NA	NA
Martin R.C.G. [222] (2011, USA)	1977–2006	14	903 (NA)	NA	NA	NA	NA	NA	NA	NA	NA

Table S7. *Cont.*

Author (Year, Country)	Time Period	% of PNENs Diagnosed as NECs	*n* Pancreatic NEC (M:W)	Age Year (Range)	Size cm (Range)	Location in Primary: *n* Caput: *n* Corpus:*n* Cauda	*n* Lymph. Met.	*n* Distant Met. at Presentation/Study Start (Sites)	*n* SC:*n* LC	Overall Survival Mo (Range)	Long-Term Survivors (>2 Years) *n*: Mo Survival (DOD, AWD or NED)
Roland C.L. [289] (2011, SEER, USA)	1980–2004	63.7 [e]	247 (NA)	NA	NA	NA	NA	NA	NA	1 year survival: 30% (ca)	NA
Sellner F. [225] (2011, Austria)	1985–2008	55	10 (NA)	NA	NA	NA	NA	NA	NA	1 year survival: 38%	0
Strosberg J.R. [226] (2011, USA)	1999–2010	7.9	32 (NA)	NA	NA	NA	NA	NA	NA	M^2: 21	NA
Welin S. [108] (2011, Sweden, Norway)	2004–2009	NA	10 (NA)	NA	NA	NA	NA	NA	NA	NA	NA
Winter J.M. [208] (2011, USA)	1970–2007	NA	6 (3:3)	M^2: 50 (27–60)	M^2: 3 (3–4.5)	6:0:0	5	NA	6 [k]:0	M^2: 20 (9–173)	3: 26, 73, 173
Faggiano A. [68] (2012, Italy)	1990–2007	NA	19 (NA)	NA	NA	NA	NA	NA	NA	NA	NA
Hamilton N.M. [217] (2012, USA)	1994–2009	11.4	19 (NA)	NA	NA	NA	NA	NA	NA	5 years survival: 57%	57% alive at 5 years
Hentic O. [85] (2012, France)	2000–2010	NA	10 (6:4)	(35–72)	NA	NA	NA	9 (NA)	3:5 (2 NA)	(11–28)	2: 28
Korse C.M [95] (2012, The Netherlands)	1994–2009	NA	9 (NA)	NA	NA	NA	NA	NA	0:9	NA	NA

Table S7. *Cont.*

Author (Year, Country)	Time Period	% of PNENs Diagnosed as NECs	*n* Pancreatic NEC (M:W)	Age Year (Range)	Size cm (Range)	Location in Primary: *n* Caput: *n* Corpus:*n* Cauda	*n* Lymph. Met.	*n* Distant Met. at Presentation/Study Start (Sites)	*n* SC:*n* LC	Overall Survival Mo (Range)	Long-Term Survivors (>2 Years) *n*: Mo Survival (DOD, AWD or NED)
Olsen [122] I.H. (2012, Denmark)	NA	NA	7 (NA)	NA	NA	NA	NA	7 (NA)	NA	M[2]: 7	NA
Rindi G. [236] (2012, Germany, Italy, U.K., the Netherlands, France)	1990–2007	5.88	63	17 < 48 years, 24: 48–61, 22: >61	NA	NA	NA	NA	NA	32.9 rate/ 100 person years	NA
Terashima T [115]. (2012, Japan)	2000–2008	NA	9 (NA)	M[2]: pancreas /biliary tract/ lliver: 46.5 (27–79)	NA	NA	NA	9 [k,l] (NA)	9 [k]:0	M[2]: 7.8 (pancreas/ bilary/liver)	NA
Yachida S. [230] (2012, USA)	1988–2010	NA	19 (9:10)	M[1]: SC: 56.6 (SD ± 10.1), M[1] LC: 55.8 (SD ± 20.6)	M[1] SC: 5.4 (SD ± 2.2), M[1] LC: 4.5 (SD ± 1.3)	10:3:5 (1 NA)	14 (1 NA)	NA	9:10	M[1] SC: 13.9 (1–25), M[1] LC: 17.9 (1–104)	4: 24, 25, 47, 107
Bertani E. [290] (2013, Italy)	1998–2008	23	10 (NA)	NA	NA	NA	NA	23 [k] (NA)	NA	NA	NA

Table S7. *Cont.*

Author (Year, Country)	Time Period	% of PNENs Diagnosed as NECs	*n* Pancreatic NEC (M:W)	Age Year (Range)	Size cm (Range)	Location in Primary: *n* Caput: *n* Corpus:*n* Cauda	*n* Lymph. Met.	*n* Distant Met. at Presentation/Study Start (Sites)	*n* SC:*n* LC	Overall Survival Mo (Range)	Long-Term Survivors (>2 Years) *n*: Mo Survival (DOD, AWD or NED)
Cherenfant J [212] (2013, USA)	1998–2011	14.1	18 (NA)	NA	2: 1–2 cm, 3: 2.1–3 cm, 2: 3.1–4 cm, 11: >4 cm	NA	NA	NA	NA	NA	NA
Delektorskaya V.V. [67] (2013, Russia)	1994–2011	11.3	8 (NA)	NA	M[1]: 12.5 (2–16)	NA	6	7 (NA)	NA	NA	NA
Henderson-Jackson, E.B. [291] (2013, USA)	1990–2007	10.2	6 (NA)	NA	NA	NA	NA	NA	NA	NA	NA
Korse C.M. [3] (2013, Netherlands Cancer Registry)	1990–2010	NA	433 [n] (NA)	NA	NA	NA	NA	NA	NA	5 years relative survival: LC 1990–2000/ 2001–2010: 44/39%. SC 1990–2000/ 2001–2010: 0/6%	NA
Kuo E.J. [292] (2013, USA)	1988–2009	14.2	110 (NA)	NA	≤2 cm: 11, >2 cm: 99	NA	NA	NA	NA	NA	NA
Liu T.C. [239] (2013, USA)	1993–2009	NA	8 (NA)	NA	NA	NA	NA	NA	NA	M[2]: 25.5	NA

Table S7. *Cont.*

Author (Year, Country)	Time Period	% of PNENs Diagnosed as NECs	*n* Pancreatic NEC (M:W)	Age Year (Range)	Size cm (Range)	Location in Primary: *n* Caput: *n* Corpus:*n* Cauda	*n* Lymph. Met.	*n* Distant Met. at Presentation/Study Start (Sites)	*n* SC:*n* LC	Overall Survival Mo (Range)	Long-Term Survivors (>2 Years) *n*: Mo Survival (DOD, AWD or NED)
Martin-Perez [210] (2013, Spain)	2001–2010	11.2 [f]	21 (NA)	NA	NA	NA	NA	NA	NA	5 years survival: 17.4%	NA
Sorbye H. [4] (2013, 12 Nordic hospitals)	2000–2009	NA	71 (NA)	NA	NA	NA	NA	NA	NA	M^2: 15 (CI 95%: 10.3–19.7)	NA
Toste P.A. [228] (2013, USA)	1989–2012	6	7 (NA)	NA	NA	NA	NA	NA	NA	NA	NA
Van der Zwan J.M. [284] (2013 RARECARE, Europe)	1995–2002	NA	128	NA	NA	NA	NA	NA	NA	NA	NA
Vélayoudom-Céphise F.L. [90] (2013, France)	2000–2005	NA	9 (NA)	NA	NA	NA	NA	NA (9 lymph and or distant metastases [l])	0:9 [k]	NA	NA
Basturk O. [125] (2014, USA)	1988–2012	NA	44 (26:18)	M^1: 59 (21–82)	M^2: 4 (2–18)	27:3:11 (3 NA)	34	11 (11 liver, 8 lymph nodes)	17:27	M^2: 11 (0–104) (of those who died)	NA: 8 alive: median follow-up 19.5 mo (0–71)
Crippa S. [224] (2014, Italy)	1990–2009	10.4	37 (NA)	NA	NA	NA	NA	NA	NA	NA	NA
Ellison, T.A. [214] (2014, USA)	1984–2011	6.5	18 (NA)	NA	NA	NA	NA	NA	NA	M^2: 20 (13–31)	9% alive at 5 years

Table S7. *Cont.*

Author (Year, Country)	Time Period	% of PNENs Diagnosed as NECs	*n* Pancreatic NEC (M:W)	Age Year (Range)	Size cm (Range)	Location in Primary: *n* Caput: *n* Corpus:*n* Cauda	*n* Lymph. Met.	*n* Distant Met. at Presentation/Study Start (Sites)	*n* SC:*n* LC	Overall Survival Mo (Range)	Long-Term Survivors (>2 Years) *n*: Mo Survival (DOD, AWD or NED)
Hashim Y.M. [293] (2014, USA)	1994–2012	11	15 (NA)	NA	NA	NA	10	0 [k]	NA	M^2: 7.1 years	NA
Liu B. [294] (2014, USA.	1988–2012	3.5	9 (NA)	NA	NA	NA	NA	NA	NA	NA	NA
Yamaguchi T. [92] (2014, Japan)	2000–2011	NA	35 [i] (NA)	NA	NA	NA	NA	NA	NA	M^2: 8.5	NA
TOTAL	1951–2012	14.6 average (24 studies included) [j] (3.9–55)	2149 [j] (M: 69/108 F: 39/108) [j]	(13–90)	(1–18)	43 (+1 isthmus): 6: 13 (+3 corpus/cauda) [j]	75/102 (74%) [j]	82/142 (58%) [j] (met. as inclusion criteria not included) (sites: 21 liver, 9 distant lymph; mentioned in 22 cases)	-	-	-

[a] Prospective study, all others are retrospective or NA; [b] only 12.3% of the total 9821 patients had a histological diagnosis of differentiation; [c] only 311/1483 PNEN cases had a histological grade; [d] only 614/2158 PNENs had a histological classification; [e] only 635/2350 PNEN cases had a histological grade; [f] only 188/483 PNEN cases had a ki-67 index; [g] only information for 16 cases; [h] only 30 included due to inclusion criteria; [i] in larger cohort 21/258 cases were diagnosed as MANEC; unknown distribution); [j] after removal of possible overlapping cohorts with lowest case numbers; [k] part of inclusion criteria; [l] described as “extensive disease”: disease beyond the organ of origin and locoregional lymph nodes; [n] might not be exact numbers: extrapolated from incidence rates; PNEN: Pancreatic Neuroendocrine Neoplasm; NEC: Neuroendocrine Carcinoma: includes WHO 2000/2004/2010 definition (poorly differentiated endocrine carcinomas, G3 tumours, small cell and large cell carcinomas); NA: Not Available; M: Men; W: Women; M^1: Mean; M^2: Median; Year: Year/s; SC: Small Cell; LC: Large Cell; Met.: Metastases; CI: Confidence Intervall; DOD: Dead of disease; AWD: Alive with disease; NED: No evidence of disease.

Table S8. Overview of Studies Including Gallbladder Neuroendocrine Carcinomas.

Author (Year, Country) [a]	Time Period	% of NECs in Relation to Gallbladder Cancer	*n* Gallbladder NEC (M:W)	*n*+ Gallstones	Age (Range)	Size cm (Range)	Symptoms	*n* Lymph Met	*n* Distant Met	*n* SC: *n* LC	*N* + Other Histological Components	Overall Survival Mo (Range)	Long-Term Survivors (>2 Years) (DOD, AWD, NED) *n*: Time until Death/End of Follow-Up Mo
Albores-Saavedra J.A. [253] (1980, Mexico)	1954–1978	3.1 [b]	5 (2:3)	5	M[1]: 64 (55–74)	NA	5 right upper quadrant pain, 2 vomiting, 2 hepatomegaly/palpable mass, 3 jaundice, 3 loss of weight, 2 acholia, 1 pruritus, 1 lymphadenopathy,	5	5 (4 distant lymph nodes, 3 liver, 1 diaphragm, 1 peritoneum , 1 pleura)	5:0	1+ well-differentiated adenocarcinoma	NA	0 (1 NA)
Albores-Saavedra J. [248] (1984, Mexico)	NA [c]	4.2	19 (2:17)	18	M[1]: 62.7 (49–79)	All except one >7 cm	NA	18	18 (4 distant lymph mets, 18 liver, 4 lung, 2 pleura, 3 peritoneum, 1 ovaries, 1 diaphragma, 1 pancreas)	19 [d]:0	4 + well-differentiated adenocarcinoma	(NA–13)	0
Guo K.J. [296] (1987, Japan)	NA	2.8	8 (3:5)	3	(44–73)	NA	NA	NA	10 (10 liver, 8 regional lymph nodes, 4 bile ducts, 4 pancreas, 4 duodenum, 4 lung, 3 peritoneum, 2 colon, 2 ovary, 4 other)	8:0	NA	(2–9) (3 NA)	1: 56
Nishihara K [252] (1993, Japan)	NA	4.1	15 (6:9)	4	M[1]: 64.5 (43–83)	NA	11: abdominal pain, 2: abdominal mass, 1: jaundice, 1: ascites	10	9 (9 liver, 3 pancreas, 2 duodenum/omentu/colon, 3 common bile duct, 1 lung) 1 NA	15 [d]:0	2 + adenocarcinoma, 1 + squamous and adenocarincoma	M[1] surival: 5.8–28.1 (2 mo–5 years 11 mo)	1: 2 years 4 mo, 5 years 11 mo

Table S8. *Cont.*

Author (Year, Country) [a]	Time Period	% of NECs in Relation to Gallbladder Cancer	*n* Gallbladder NEC (M:W)	*n*+ Gallstones	Age (Range)	Size cm (Range)	Symptoms	*n* Lymph Met	*n* Distant Met	*n* SC: *n* LC	*N* + Other Histological Components	Overall Survival Mo (Range)	Long-Term Survivors (>2 Years) (DOD, AWD, NED) *n*: Time until Death/End of Follow-Up Mo
Moskal T.L. [251] (1999, USA)	1971–1997	3.5	5 (2:3)	4	M^2: 69 (40–71)	NA	4: abdominal pain, 1: fever, 1: weight loss	5	1–5 (3 liver, 3 lungs, 1 peritoneum, 1 bone)	5 [d]:0	3 + adenocarcinoma	M^2: 31 (13–189)	3: 31, 44, 189
Maitra A [250] (2001, USA)	NA	NA	12 (7:5)	12	M^1: 69 (37–85)	M^1: 3.0 (0–8)	12: Cholecystitis	5	7 (4 liver, 3 soft tissue, 1 pancreas)	12 [d]:0	7 + adenocarcinoma *in situ*, 4 + adenocarcinoma, 1 + squamous cell carcinoma, 1 + high grade sarcoma	M^2: 9 (3–25 mo)	1: 25
Brenner B. [7] (2004, USA)	1980–2002	NA	5 (NA)	NA	NA	NA	NA	NA	NA	5 [d]:0	NA	NA	NA
Lee S.S. [15] (2007, Korea)	1995–2004	NA	15 (NA) [e]	NA	NA	NA	NA	NA	86% liver mets, peritoneum mets 28%, bone mets 7%	15 [d]:0	NA	M^2: 4 (1.5–17.5)	0
Lepage C. [1] (2007, UK)	1986–1999	NA	10 (NA)	NA	NA	NA	NA	NA	NA	10 [d]:0	NA	Relative 1 year survival: 28.2	NA

Table S8. *Cont.*

**Author (Year, Country) [a]**	**Time Period**	**% of NECs in Relation to Gallbladder Cancer**	***n* Gallbladder NEC (M:W)**	***n*+ Gallstones**	**Age (Range)**	**Size cm (Range)**	**Symptoms**	***n* Lymph Met**	***n* Distant Met**	***n* SC: *n* LC**	***N* + Other Histological Components**	**Overall Survival Mo (Range)**	**Long-Term Survivors (>2 Years) (DOD, AWD, NED) *n*: Time until Death/End of Follow-Up Mo**
Albores-Saavedra [249] (2009, USA)	1973–2005	0.38: gallbladder. 0.19: EHBD	71 (25:46) [f]	NA	M[1]: gallbladder: 67.5, EHBD: 68.4	22: >2 cm, 2: ≤2cm, 27 NA	NA	NA	37 (5 NA)	71:0	NA	5 years survival gallbladder: 8%, 5 years survival EHBD: 0%	8% alive at 5 years
Lepage C. [79] (2009, EUROCARE)	1985–1994	NA	48 (NA)	NA	NA	NA	NA	NA	NA	123 [d]:0	NA	5 years relative survival: 10.3	NA
Wong Y.N. [65] (2009, UK)	1970–2004	NA	15 (NA)	NA	NA	NA	NA	NA	NA	15 [d]:0	NA	NA	NA
TOTAL	**(1954–2005)**	**(0.38–4.2)**	**190** [g]	**38/51 (74.5%)** [g]	**(37–85)** [g]			**38/51 (75%)** [g]	**72/122 (59%)** [g]				

NEC: Neuroendocrine Carcinoma: includes WHO 2000/2004/2010 definition (poorly differentiated endocrine carcinomas, G3 tumours, small cell and large cell carcinomas); NA: Not Available; M: Men; W: Women; M[1]: Mean; M[2]: Median; Mo: Month/s; Year: Year/s; SC: Small Cell; LC: Large Cell; Met.: Metastases; EHBD: Extra Hepatic Bile Duct; CI: Confidence Intervall; DOD: Dead of disease; AWD: Alive with disease; NED: No evidence of disease; [a] all studies are retrospective; [b] of all invasive gallbladder carcinomas; [c] over a 25-year period; [d] part of inclusion criteria; [e] 14 gallbladder, 1 common bile duct; [f] 54 gallbladder, 17 extrahepatic bile duct; [g] after removal of possible overlapping cohorts with lowest patient numbers.

Table S9. Overview of Studies Including Small Intestinal Neuroendocrine Carcinomas.

Author (Year, Country) [a]	Time Period	% of NECs Relative to Carcinomas of the Small Intestine	*n* Small Intestinal NECs (M:W)	Age Year (Range)	Size cm (Range)	Location in Primary	Lymph. Mets	Distant Mets. at Presentation	*n* SC:*n* LC	Other Histological Components	Overall Survival Mo (Range)	Long-Term Survivors (>2 Years) (DOD, AWD, NED) *n*: Time until Death/End of Follow-Up Mo)
Nassar H. [255] (2005, USA)	1965–2004	2%–3% of ampullary tumours	14 (10:4)	M[1]: 70	M[1]: 2.5 (0.8–4)	14 Ampullar of Vater	10 (1 NA)	3 (2 brain, 1 liver)	6:8	7 + adenoma, 1 + squamous	M[1]: 14.5 (4–30) [b] 4 alive: 6,10,17,48	1: 48
Lepage C. [1] (2007, UK)	1986–1999	NA	8 (NA)	NA	NA	NA	NA	NA	8 [c]:0	NA	1 years relative survival: 27.1%	NA
Ploeckinger U. [73] (2007, Germany)	1999–2007	NA	13 (NA)	NA	NA	NA	NA	NA	NA	NA	NA	NA
Landry C.S. [260] (2008, USA)	1977–2004	NA	48 (NA)	NA	NA	NA	NA	NA	NA	NA	NA	NA
Albores-Saavedra J. [258] (2010, USA)	1973–2006	0.25	15 (NA)	NA	NA	15 Ampulla of Vater	NA	NA	9:6	NA	5 years survival: 15.7%	NA
Grossman R.A. [11] (2011, USA)	1973–2006	NA	13 (NA)	NA	NA	NA	NA	NA	13 [c]:0	NA	NA	NA
Dumitrascu T. [256] (2012, Romania)	NA	NA	9 [d] (NA)	NA	(1–3.5)	9 Ampulla of Vater	5.00	NA	NA	NA	(7–52)	1: 52
Korse C.M. [3] (2013, the Netherlands) [e]	1990–2010	NA	97 (NA)	NA	NA	NA	NA	NA	NA	NA	5 years relative survival rate: LC 1990–2000/2001–2010: 25/27%, Small cell 1990–2000/2001–2010: 7/3%	NA

Table S9. *Cont.*

Author (Year, Country) [a]	Time Period	% of NECs Relative to Carcinomas of the Small Intestine	*n* Small Intestinal NECs (M:W)	Age Year (Range)	Size cm (Range)	Location in Primary	Lymph. Mets	Distant Mets. at Presentation	*n* SC:*n* LC	Other Histological Components	Overall Survival Mo (Range)	Long-Term Survivors (>2 Years) (DOD, AWD, NED) *n*: Time until Death/End of Follow-Up Mo)
Randle R.W. [257] (2014, USA)	1988–2009	NA	58 (NA)	NA	NA	38 duodenum, 20 Ampulla of Vater	NA	NA	NA	NA	NA	NA
Untch B.R. [297] (2014, USA)	1983–2011	NA	10 (NA)	65 ± 11 years	2.6 ± 1	8 Ampulla of Vater, 2 other duodenal	7	0 [f]	NA	NA	M^2: 15	NA
Yamaguchi T. [92] (2014, Japan)	2000–2011	NA	6 [g] (NA)	NA	NA	NA	NA	NA	NA	NA [g]	M^2: 29.7 [g]	NA
TOTAL	(1965–2011)	-	205.00	-	-	-	-	-	-	-	-	-

NEC: Neuroendocrine Carcinoma: includes WHO 2000/2004/2010 definition (poorly differentiated endocrine carcinomas, G3 tumours, small cell and large cell carcinomas); NA: Not Available; M: Men; W: Women; M^1: Mean; M^2: Median; Mo: Month/s; Year: Year/s; SC: Small Cell; LC: Large Cell; Met.: Metastases; CI: Confidence Intervall; DOD: Dead of disease; AWD: Alive with disease; NED: No evidence of disease; [a] all studies are retrospective; [b] only those who died; [c] part of inclusion criteria; [d] total from a review of the literature after removal of studies already presented; [e] not all numbers may be exact due to extrapolation from incidence rates; [f] all tumours with metastases were excluded; [g] original cohort of 258 NECs includes 21 MANECs, unknown distribution.

Table S10. Overview of Studies Including Colorectal Neuroendocrine Carcinomas.

Author (Year, Country)	Time Period	% of Colorectal Cancers	*n* Colorectal NEC (M:W)	*n* Colonic NEC: *n* Rectal NEC (*n* Specified Location)	Age Year (Range)	Size cm (Range)	*n* Lymph. Met.	*n* Distant Met. at Presentation/Study Start (Specified Sites)	*n* SC:*n* LC	Other Histological Components	Overall Survival Mo (Range)	Long-Term Survivors (>2 Years, DOD, AWD, NED), *n*: Time Til Death/Follow-Up Mo
Mills S.E. [276] (1983, USA)	NA	NA	5 (2:3)	2:3 (2 cecum, 3 rectum)	(53–74)	(0.3–7)	5	4 (4 liver, 1 peritoneum, 1 lung, 1 bone marrow, 1 paraaortic lymph nodes)	5:0	4 + adenomatous neoplasms	(1–3)	0
Wick M.R. [275] (1987, USA)	NA	NA	10 (5:5)	NA	Average: 63 (40–75)	(3–13)	6	7 (7 liver, other NA)	10:0	1 + adenocarcinoma	Average: 5 (NA–11)	0
Staren, E.D. [263] (1988, USA)	1980–1987	1.9	13 (7:6)	9:4 (5 cecum, 2 sigmoid colon, 1 transverse colon, 1 descending colon, 4 rectum)	M[2]: 72 (28–89)	NA	8	6 (5 liver, 1 bone, unknown if distant lymph)	7:5	8 + glandular/squamous differentiation, 3 glandular/squamous differentiation + adenoma	M[2]: 7 (2–68)	2: 30, 68
Burke A.B. [266] (1990, USA) [a]	1986–1988	NA	38 (22:16)	15:22 (7 left colon, 8 right colon, 22 rectum) (1 NA)	Mean: 63	NA	89%	71%	38 [b]:0	17 + adenoma, 10 + adenocarcinoma, 9 + squamous change	36% alive after 5 mo follow-up	NA
Gaffey M.J. [267] (1990, USA)	NA	NA	22 (12:10)	15:7 (9 cecum, 5 sigmoideum, 1 transversum, 7 rectum)	SC M[1]: 64, intermediate cell M[1]: 66 (34–87)	(0.3–15)	18	15 (13 liver, 1 bone, 1 peritoneum, 1 diaphragm, 1 duodenum)	6:0 (16 intermediate/small)	15 + adenomatous component, 6 + squamous component	Average 8.2 (1–72) 7 NA	1: 72

Table S10. *Cont.*

Author (Year, Country)	Time Period	% of Colorectal Cancers	*n* Colorectal NEC (M:W)	*n* Colonic NEC: *n* Rectal NEC (*n* Specified Location)	Age Year (Range)	Size cm (Range)	*n* Lymph. Met.	*n* Distant Met. at Presentation/Study Start (Specified Sites)	*n* SC:*n* LC	Other Histological Components	Overall Survival Mo (Range)	Long-Term Survivors (>2 Years, DOD, AWD, NED), *n*: Time Til Death/Follow-Up Mo
Spread C. [271] (1994, Canada)	1964–1988	NA	16 (NA)	16:0 (NA)	NA	NA	22% Dukes' stage C	64% Dukes' stage D (48% liver, 30% liver + other site, 22% other site)	0:16 [b]	NA	M^2: 4.5 [c]	NA
Galanis E. [9] (1997, USA)	1974–1994	NA	13 (NA)	NA	NA	NA	NA	NA (6 LD)	13 [b]: 0	NA	NA	NA
Vortmeyer A.O. [272] (1997, USA)	NA	NA	9 (5:4)	7:2 (2 cecum, 4 ascending colon, 1 transverse, 2 rectum)	Average: 49 (36–60)	NA	5	3 (3 liver)	NA	4 + adenocarcinoma, 1 + adenoma, 2 + adenocarcinoma and adenoma	NA	NA
Ordónez N.G. [36] (2000, USA)	NA	NA	11 (NA)	11:0 (NA)	NA	NA	NA	NA	11 [b]:0	NA	NA	NA
Akintola-Ogunremi O. [274] (2003, USA)	NA	NA	66 (35:31)	NA	M^1: 62 (30–91)	NA	92% + lymph and/or distant metastases	92% + lymph and/or distant metastases (mostly liver)	36:30	32 + adenoma and/or adenocarcinoma	M^2: 8–9	2: >3 years
Pizzi S. [38] (2003, Italy)	NA	NA	9 (6:3)	9:0 (NA)	(63–81)	NA	6	3 (3 liver, 1 lung) unknown if lymph are distant mets	NA	NA	NA	NA

Table S10. *Cont.*

Author (Year, Country)	Time Period	% of Colorectal Cancers	*n* Colorectal NEC (M:W)	*n* Colonic NEC: *n* Rectal NEC (*n* Specified Location)	Age Year (Range)	Size cm (Range)	*n* Lymph. Met.	*n* Distant Met. at Presentation/Study Start (Specified Sites)	*n* SC:*n* LC	Other Histological Components	Overall Survival Mo (Range)	Long-Term Survivors (>2 Years, DOD, AWD, NED), *n*: Time Til Death/Follow-Up Mo
Stelow, E.B. [282] (2003, USA)	NA	NA	15 (9:6)	10:3 (6 right colon, 4 sigmoid colon, 3 rectum) (2NA)	M[1]: 73 (44–87)	M[1]: 6.6 (0.4–15)	NA	NA	15 [b]:0	11 + adenoma	NA	NA
Bernick P.E. [264] (2004, USA)	1977–1998	<1%	38 (17:21) [d]	17:20 (8 cecum, 2 right colon, 1 hepatic flexure, 3 transverse colon, 3 sigmoid colon), 14 rectum, 6 anal canal, 1 appendix)	M[1]: 57 (29–86) [d]	NA	NA	25 (2 NA) [d]	22:16 [d]	NA	M[2]: 10.5 (95% CI: 6.7–18.9) [d]	26% alive at 2 years [d]
Brenner B. [7] (2004, USA)	1980–2002	NA	25 (NA)	13:12 (13 colon, 10 rectum, 2 anal canal)	NA	NA	NA	NA	25 [b]:0	11 + adenoma, 1 + ulcerative colitis	NA	NA
Furlan D. [39] (2004, Italy)	NA	NA	7 (4:3)	NA	(50–77)	(2.5–7)	5	2 (2 liver) NA if lymph were also mets, 2 NA	NA	NA	Average: 15 (1–72)	1: 72
La Rosa S. [58] (2004, Italy)	NA	NA	10 (6:4)	6:4 (6 right colon, 4 rectum)	Colon: M[1]: 67.5 (50–92), Rectum: m M[1]: 61 (56–67)	M[1] colon: 4 (2.5–7), Rectum: M[1] 3.7 (1.3–7)	NA	9	NA	NA	NA	NA

Table S10. *Cont.*

Author (Year, Country)	Time Period	% of Colorectal Cancers	*n* Colorectal NEC (M:W)	*n* Colonic NEC: *n* Rectal NEC (*n* Specified Location)	Age Year (Range)	Size cm (Range)	*n* Lymph. Met.	*n* Distant Met. at Presentation/Study Start (Specified Sites)	*n* SC:*n* LC	Other Histological Components	Overall Survival Mo (Range)	Long-Term Survivors (>2 Years, DOD, AWD, NED), *n*: Time Til Death/Follow-Up Mo
Furlan D. [43] (2005, Italy)	NA	NA	10 (6:4)	NA	(50–77)	All intermediate -large or intermediate	7	4 (4 liver), NA if lymph mets were distant	NA	NA	(1–72), 2 NA,	1: 72
Grabrowski P. [45] (2005, Germany)	1981–2001	NA	8 (NA)	4:4 (1 cecum/ascending colon, 3 colon, 4 rectum)	NA	All >2 cm	NA	8	NA	NA	NA	NA
Shida T. [50] (2005, Japan)	1999–2004	NA	6 (3:3)	3:3 (NA)	(45–78)	NA	NA	3	3:3 (moderate)	NA	Average 7 (1–14)	NA
Haider K. [12] (2006, Canada)	1971–2002	NA	8 (4:4)	NA	NA	NA	NA	4 ED	8 [b]:0	NA	NA	NA
Hainsworth J.D. [84] (2006, USA) [a]	1999–2005	NA	9 (NA)	NA	NA	NA	NA (9 lymph nodes or distant metastases)	9 (lymph nodes or distant metastases)	NA	NA	NA	NA
Li A.F.Y. [42] (2006, Taiwan)	1985–2004	NA	6 (3:3)	6:0 (NA)	(32–79)	NA	6	5	NA	NA	Average: 14 (0–35)	2: 34, 35

Table 10. *Cont.*

Author (Year, Country)	Time Period	% of Colorectal Cancers	*n* Colorectal NEC (M:W)	*n* Colonic NEC: *n* Rectal NEC (*n* Specified Location)	Age Year (Range)	Size cm (Range)	*n* Lymph. Met.	*n* Distant Met. at Presentation/Study Start (Specified Sites)	*n* SC:*n* LC	Other Histological Components	Overall Survival Mo (Range)	Long-Term Survivors (>2 Years, DOD, AWD, NED), *n*: Time Til Death/Follow-Up Mo
Uccella S. [37] (2006, Italy)	1982–2004	NA	11 (8:3)	NA	(50–85)	(1.3–7)	NA	(11 "malignant")	NA	NA	NA	NA
Kang H. [5] (2007, USA)	1991–2000	0.3%	455 (222: 233)	65.7%:29.6% (44.9% right colon, 6.9% transverse colon, 13.9% left colon, 29.6% rectum) (4.6% NA)	M[1]: 67.9 (SD ± 14.4)	NA	NA	62.1% distant, 27.1% regional, 10.8% localized	NA	NA	5 years relative survival: 21.4%	21.4%: alive at 5-year
Lee S.S. [15] (2007, Korea)	1995–2004	NA	5 (NA)	2:3 (NA)	NA	NA	NA	NA	5 [b]:0	NA	NA	NA
Lepage C. [1] (2007, UK)	1986–1999	NA	95 (NA)	NA	NA	NA	NA	NA	95 [b]:0	NA	1 year relative survival: 27%	NA
Ploeckinger U. [73] (2007, Germany)	1999–2007	NA	14 (NA)	NA	NA	NA	NA	NA	NA	NA	NA	NA
Arnold C.N. [265] (2008, Germany)	NA	NA	28 (13:6) (9NA)	25:3 (NA)	(29–92)	NA	NA	(28 "malignant")	NA	NA	(0–57 weeks) (10 NA)	6: 18–99 weeks

Table S10. *Cont.*

Author (Year, Country)	Time Period	% of Colorectal Cancers	*n* Colorectal NEC (M:W)	*n* Colonic NEC: *n* Rectal NEC (*n* Specified Location)	Age Year (Range)	Size cm (Range)	*n* Lymph. Met.	*n* Distant Met. at Presentation/Study Start (Specified Sites)	*n* SC:*n* LC	Other Histological Components	Overall Survival Mo (Range)	Long-Term Survivors (>2 Years, DOD, AWD, NED), *n*: Time Til Death/Follow-Up Mo
Grabowski P. [46] (2008, Germany)	1981–2001	NA	8 (NA)	4:4 (1 cecum/ascending colon, 3 colon, 4 rectum)	NA	All >2 cm	NA	8	NA	NA	NA	NA
Landry C.S. [279] (2008, USA)	1973–2004	NA	115 (NA)	115:0 (NA)	NA	NA	NA	NA	NA	NA	NA	NA
Landry C.S. [280] (2008, USA)	1973–2004	NA	25 (NA)	0:25 (NA)	NA	NA	NA	NA	NA	NA	NA	NA
Shia, J. [29] (2008, USA)	NA	NA	40 (NA)	NA	NA	NA	NA	NA	11:20 (+9 mixed)	NA	NA	NA
Shida T. [51] (2008, Japan)	1999–2007	NA	6 (3:3)	3:3 (NA)	(47–78)	NA	NA	4	3:3	NA	Average: 12 (1–48)	1: 48
Lepage C. [79] (2009, EUROCARE)	1985–1994	NA	114	NA	NA	NA	NA	NA	109 [b]:0	NA	5 years relative survival: 18	NA
Wong Y.N. [65] (2009, UK)	1970–2004	NA	60 (NA)	NA	NA	NA	NA	NA	60 [d]:0	NA	NA	NA
Catena L. [57] (2010, Italy)	1984–2009	NA	6 (NA)	6:0 (NA)	NA	NA	NA	NA	NA	NA	NA	NA

Table S10. *Cont.*

Author (Year, Country)	Time Period	% of Colorectal Cancers	*n* Colorectal NEC (M:W)	*n* Colonic NEC: *n* Rectal NEC (*n* Specified Location)	Age Year (Range)	Size cm (Range)	*n* Lymph. Met.	*n* Distant Met. at Presentation/Study Start (Specified Sites)	*n* SC:*n* LC	Other Histological Components	Overall Survival Mo (Range)	Long-Term Survivors (>2 Years, DOD, AWD, NED), *n*: Time Til Death/Follow-Up Mo
Li A.F.Y. [16] (2010, Taiwan)	1989–2008	NA	13 (10:3)	NA	M[1]: 61.9 ± 18.5	NA	NA	12 ED	13 [b]:0	NA	M[2]: 6 ± 1.59 (SD)	0
Li A.F.Y. [34] (2010, Taiwan)	1989–2009	NA	15 (11:4)	NA	M[1]: 54 ± 19	NA	NA	15 ED	15 [b]:0	0	M[2]: 5.1 ± 3.1	NA
Niederle M.B. [66] (2010, Austria) [a]	2004–2005	NA	8 (NA)	4:4 (NA)	NA	NA	NA	NA	8:0	NA	NA	NA
Shida T. (2010, Japan) [56]	1999–2007	NA	6 (2:4)	2:4 (NA)	(47–84)	NA	NA	3	2:4	NA	Average: 27 (1–65)	3: 65, 50, 36
Grossman R.A. [11] (2011, USA)	1973–2006	NA	342 (NA)	NA	NA	NA	NA	NA	342 [b]:0	NA	NA	NA
Patta A. [269] (2011, USA)	2003–2010	NA	8 (5:3)	3:5 (NA)	M[2]: 64 (31–83)	NA	5	8 (8 liver, 2 lung, 1 peritoneum, 1 bone, 3 distant lymph)	NA	NA	M[2]: 9.5 (3.5–17)	0
Welin S. [108] (2011, Sweden, Norway)	2004–2009	NA	5 (NA)	NA	NA	NA	NA	5	NA	NA	NA	NA

Table S10. *Cont.*

Author (Year, Country)	Time Period	% of Colorectal Cancers	*n* Colorectal NEC (M:W)	*n* Colonic NEC: *n* Rectal NEC (*n* Specified Location)	Age Year (Range)	Size cm (Range)	*n* Lymph. Met.	*n* Distant Met. at Presentation/Study Start (Specified Sites)	*n* SC:*n* LC	Other Histological Components	Overall Survival Mo (Range)	Long-Term Survivors (>2 Years, DOD, AWD, NED), *n*: Time Til Death/Follow-Up Mo
Faggiano A. [68] (2012, Italy)	1990–2007	NA	7 (NA)	NA	NA	NA	NA	NA	NA	NA	NA	NA
Korse C.M [95] (2012, The Netherlands)	1994–2009	NA	5 (NA)	5:0 (NA)	NA	NA	NA	NA	2:3	NA	NA	NA
La Rosa [268] (2012, Italy)	1980–2010	NA	27 (15:12)	26: 0 (17 right colon, 9 left colon) (1 NA)	Average: 67 (34–85)	NA	19	8	12:11 (4 mixed)	11 + non-neuroendocrine component	1 year survival: *ca.* 20%	4: 11–257 (M[1]: 190)
Olsen I.H. [117] (2012, Denmark)	2007–2009	NA	6 (NA)	NA	NA	NA	NA	6	NA	NA	NA	NA
Olsen I.H. [122] (2012, Denmark)	NA	NA	5 (NA)	NA	NA	NA	NA	5	NA	NA	NA	NA
Chagpar R. [273] (2013, USA)	NA	NA	528 (NA)	NA	NA	NA	NA	NA	NA	NA	NA	NA
Delektorskaya V.V. [67] (2013, Russia)	1994–2011	NA	8 (NA)	NA	NA	M[1]: 4.3 (2–9)	6	4	NA	NA	NA	NA

Table S10. *Cont.*

**Author (Year, Country)**	**Time Period**	**% of Colorectal Cancers**	***n* Colorectal NEC (M:W)**	***n* Colonic NEC: *n* Rectal NEC (*n* Specified Location)**	**Age Year (Range)**	**Size cm (Range)**	***n* Lymph. Met.**	***n* Distant Met. at Presentation/Study Start (Specified Sites)**	***n* SC:*n* LC**	**Other Histological Components**	**Overall Survival Mo (Range)**	**Long-Term Survivors (>2 Years, DOD, AWD, NED), *n*: Time Til Death/Follow-Up Mo**
Feng S.T. [82] (2013, China)	2009–2013	NA	5 (NA)	0:5 (NA)	NA	NA	NA	NA	NA	NA	NA	NA
Furlan D. [281] (2013, Italy)	1989–2009	NA	21 (13:8)	21:0 (10 right colon, 11 left colon)	M^2: 69.5 (44–85)	NA	NA	16 stage III–IV (AJCC)	NA	NA	NA	2: 93, 215
Korse C.M [3]. (2013, Netherlands Cancer Registry) [e]	1990–2010	NA	372 (NA)	NA	NA	NA	NA	NA	NA	NA	5 years relative survival rate: LC 1990–2000/ 2001–2010: 24/22%. SC 1990–2000/ 2001–2010 12/9%.	NA
Sorbye H. [4] (2013, 12 Nordic hospitals)	2000–2009	NA	82 (NA)	61:21 (NA)	NA	NA	NA	NA	65% rectal tumours: SC, 30% of colon tumours:SC	NA	M^2: colon: 8 (CI 95%: 6.0–9.9), rectum: 10 (CI 95%: 8.4–13.6)	NA

Table S10. *Cont.*

Author (Year, Country)	Time Period	% of Colorectal Cancers	*n* Colorectal NEC (M:W)	*n* Colonic NEC: *n* Rectal NEC (*n* Specified Location)	Age Year (Range)	Size cm (Range)	*n* Lymph. Met.	*n* Distant Met. at Presentation/Study Start (Specified Sites)	*n* SC:*n* LC	Other Histological Components	Overall Survival Mo (Range)	Long-Term Survivors (>2 Years, DOD, AWD, NED), *n*: Time Til Death/Follow-Up Mo
Van der Zwan J.M. [284] (2013 RARECARE, Europe)	1995–2002	NA	172	86	NA	NA	NA	NA	NA	NA	NA	NA
Aytac E. [262] (2014, USA)	1993–2011	0.33%	25 (8:17)	9:16 (4 right colon, 3 transverse colon, 2 sigmoid colon, 7 rectum, 9 anal canal)	M[1]: 56.4 ± 2.7	NA	NA	9 (7 liver, 3 lung, 2 brain)	NA	6+ adenocarcinoma, 1+ adenosquamous component	NA	NA
Lee J.L. [25] (2014, Korea)	2000–2010	0.16%	18 (15:5) [f]	12:8 (2 cecum, 4 ascending colon, 1 flexure, 1 transverse colon, 4 left colon, 8 rectum) [f]	M[1]: 64 years ± 11 [f]	M[1]: 5.1 ± 2.5 [f]	NA	10 (6 liver, 2 distant lymph, 2 multi liver + perineal seeding) [f]	8: 6 (4 mixed, 2 MANEC)	NA	M[2]: 16 [f]	NA
Smith J.D. [270] (2014, USA)	1991–2010	NA	126 (62:64)	70: 58 (38 colon, 23 cecum, 9 sigmoid, 53 rectum, 5 anal canal)	M[2]: 56 (30–91)	M[2]: 4 (0.1–11)	NA	85 (64 liver, 11 lung, 10 bone, 8 retroperitoneal lymph nodes, 7 peritoneum, 5 inguinal lymph node, 2 brain)	49:23 (18 collision tumour + adenocarcinoma)	4 + adenocarcinoma	M[2]: 13.2	3: M[2] 37 mo follow-up

Table S10. *Cont.*

**Author (Year, Country)**	**Time Period**	**% of Colorectal Cancers**	***n* Colorectal NEC (M:W)**	***n* Colonic NEC: *n* Rectal NEC (*n* Specified Location)**	**Age Year (Range)**	**Size cm (Range)**	***n* Lymph. Met.**	***n* Distant Met. at Presentation/Study Start (Specified Sites)**	***n* SC:*n* LC**	**Other Histological Components**	**Overall Survival Mo (Range)**	**Long-Term Survivors (>2 Years, DOD, AWD, NED), *n*: Time Til Death/Follow-Up Mo**
Yamaguchi T. [92] (2014, Japan)	2000–2011	NA	31 [g]	NA	NA	NA	NA	NA	NA	NA	M[2]: 7.6 [g]	NA
TOTAL	**1964–2010**	**0.3–1.9 [h]**	**2026 [h] (M: 436/841 (52%), W: 405/841 (48%))**	**COLON: 410/594 [h], RECTUM: 170/594 [h]**	**(29–92)**	**(0.1–11)**	**79/112 (71%) [h,i]**	**199/307 (65%) (sites when mentioned: 105 liver, 19 peritoneum, 16 distant lymph metastases, 14 lung, 12 bone, 2 brain, 1 diaphragma, 1 duodenum) [h,i]**	-	-	-	-

NEC: Neuroendocrine Carcinoma: includes WHO 2000/2004/2010 definition (poorly differentiated endocrine carcinomas, G3 tumours, small cell and large cell carcinomas); NA: Not Available; M: Men; W: Women; M[1]: Mean; M[2]: Median; Mo: Month/s; Year: Year/s; SC: Small Cell; LC: Large Cell; Met.: Metastases; CI: Confidence Intervall; DOD: Dead of disease; AWD: Alive with disease; NED: No evidence of disease; LD: tumour confined to a localized anatomic region (the oesophagus/perioesophageal tissue/adjacent organs) with/without locoregional lymph nodes metastases (encompassable within a tolerable radiation therapy treatment portal), ED: outside LD; AJCC: American Joint Committee on Cancer; [a] Prospective study, all others NA or retrospective; [b] Part of inclusion criteria; [c] Includes moderately differentiated and well differentiated tumours; [d] Includes 1 appendix NEC; [e] Not all numbers are exact- from incidence rates.; [f] Includes 2 MANEC (unknown distribution); [g] Orignal cohort (mixed NEC primaries) includes 21 MANECs, unknown distribution; [h] after removal of possible overlapping cohorts with lowest case numbers; [i] procent values and LD/ED not included.

© 2015 by the authors; licensee MDPI, Basel, Switzerland. This article is an open access article distributed under the terms and conditions of the Creative Commons Attribution license (http://creativecommons.org/licenses/by/4.0/).
